# Goblet cells mechanically breach the epithelial barrier in gut homeostasis

**DOI:** 10.1038/s41467-026-76034-0

**Published:** 2026-07-25

**Authors:** Justine Creff, Yuan He, Sandra Bernat-Fabre, Etienne Buscail, Salomé Neuvendel, Vishnu Krishnakumar, Dhriti Saumya, Laurent Malaquin, Thomas Mangeat, Shi-Lei Xue, Denis Krndija

**Affiliations:** 1 Centre de Biologie Intégrative (CBI), University of Toulouse, CNRS, Toulouse, France; 2 Department of Materials Science and Engineering, School of Engineering, Westlake University, Hangzhou, Zhejiang PR China; 3 Department of Digestive Surgery, Colorectal Surgery Unit, Toulouse University Hospital, Toulouse, France; 4 LAAS-CNRS, University of Toulouse, CNRS, Toulouse, France; 5 LITC Core Facility, Centre de Biologie Intégrative (CBI), University of Toulouse, CNRS, Toulouse, France

**Keywords:** Tight junctions, Biological physics, Myosin

## Abstract

The intestinal epithelium forms a tight barrier against the harsh luminal environment. Absorptive enterocytes have a polygonal, columnar morphology, while mucus-producing goblet cells exhibit a rounded apical shape and a voluminous cell body, raising the question of how epithelial integrity is preserved in tissues with such morphological heterogeneity. Here, we show that, under homeostatic conditions in vivo, goblet cells mechanically induce tight-junction fractures between neighboring enterocytes. This effect is exacerbated by goblet cell hypertrophy and is associated with increased gut permeability. Using in vivo and organoid models, combined with pharmacological, genetic and mechanical perturbations and theoretical modeling, we demonstrate that these fractures arise from a force imbalance at cell interfaces: goblet cells exert pressure on adjacent enterocytes, whose junctional rupture depends on tissue rheology controlled by myosin II. Our findings uncover a mechanical role for goblet cells in epithelial cohesion and barrier regulation, revealing how cellular heterogeneity shapes tissue integrity.

## Abstract (web summary)

This study shows that goblet cells physically compress neighboring intestinal cells, creating fractures in cell–cell junctions that weaken epithelial cohesion, increase gut permeability and reveal how tissue mechanics shape the gut barrier.

## Introduction

Epithelia are specialized tissue barriers, with a critical role in separating the internal and external environment and thus maintaining normal tissue function and homeostasis^1^. The intestinal epithelium functions as a protective barrier throughout adult life, shielding the organism from continuous environmental insults^2^, including extrinsic mechanical stresses from luminal contents and muscle-driven peristalsis^3,4^ as well as intrinsic mechanical stresses due to rapid epithelial turnover^5^. Adhesive cell junctions – including tight junctions (TJs), adherens junctions (AJs), and desmosomes – enable epithelial barrier, mechanical integrity, and collective cell migration^5–7^. TJs are continuous belt-like structures that encircle and seal the paracellular space and regulate the barrier permeability^8^. AJs, which lie just beneath the TJs, couple the cytoskeletons of neighboring cells by linking transmembrane E-cadherin belts to the cortical actomyosin network, which stabilizes E-cadherin complexes^9–11^. As a consequence, AJs are under tension and transmit subcellular forces exerted by actomyosin networks to the cortex^12^. Junctional complexes are mechanosensitive and mechanoadaptive, integrating subcellular and tissue-level forces, and modulating cell behavior – e.g., junctional reinforcement upon mechanical load to maintain barrier integrity and tissue cohesion^10,11,13,14^. Finally, desmosomal plaques are located basolaterally beneath the AJs and are associated with intermediate filaments. They were recently shown to passively respond to extrinsic forces by strain-stiffening, contributing to the mechanical resilience of epithelial tissues^15,16^.

The intestinal epithelium consists of at least six different cell types, each with specialized functions and morphologies. Enterocytes, the main cell type, are absorptive cells with a columnar shape and a polygonal apical surface. Mucus-producing goblet cells (GCs), interspersed among enterocytes, are the second most abundant cell type and are characterized by their bulky, round-shaped bodies, due to the presence of large secretory granules^17–19^. While numerous studies have focused on the secretory function of GCs, the impact of their unique geometry and mechanical properties on their integration into the epithelial tissue and the intestinal barrier remains entirely unknown.

Here, we report that under homeostatic conditions in vivo, GCs are physically associated with junctional fractures that occur between neighboring enterocytes in the small intestine. Using a combination of 3D tissue imaging, in vivo (mouse models), ex vivo (explants), and in vitro (organoids) force perturbations and theoretical modeling, we found that fracturing arises from the intrinsic mechanical properties and interplay between GCs and adjacent enterocytes. On the one hand, GCs compress and deform adjacent enterocytes, straining their intercellular junctions and promoting fracturing; on the other hand, fracturing depends on global tissue mechanics (rheology), which is controlled by myosin II activity.

## Results

### Goblet cells are associated with tight-junction fractures

**Fig. 1:**
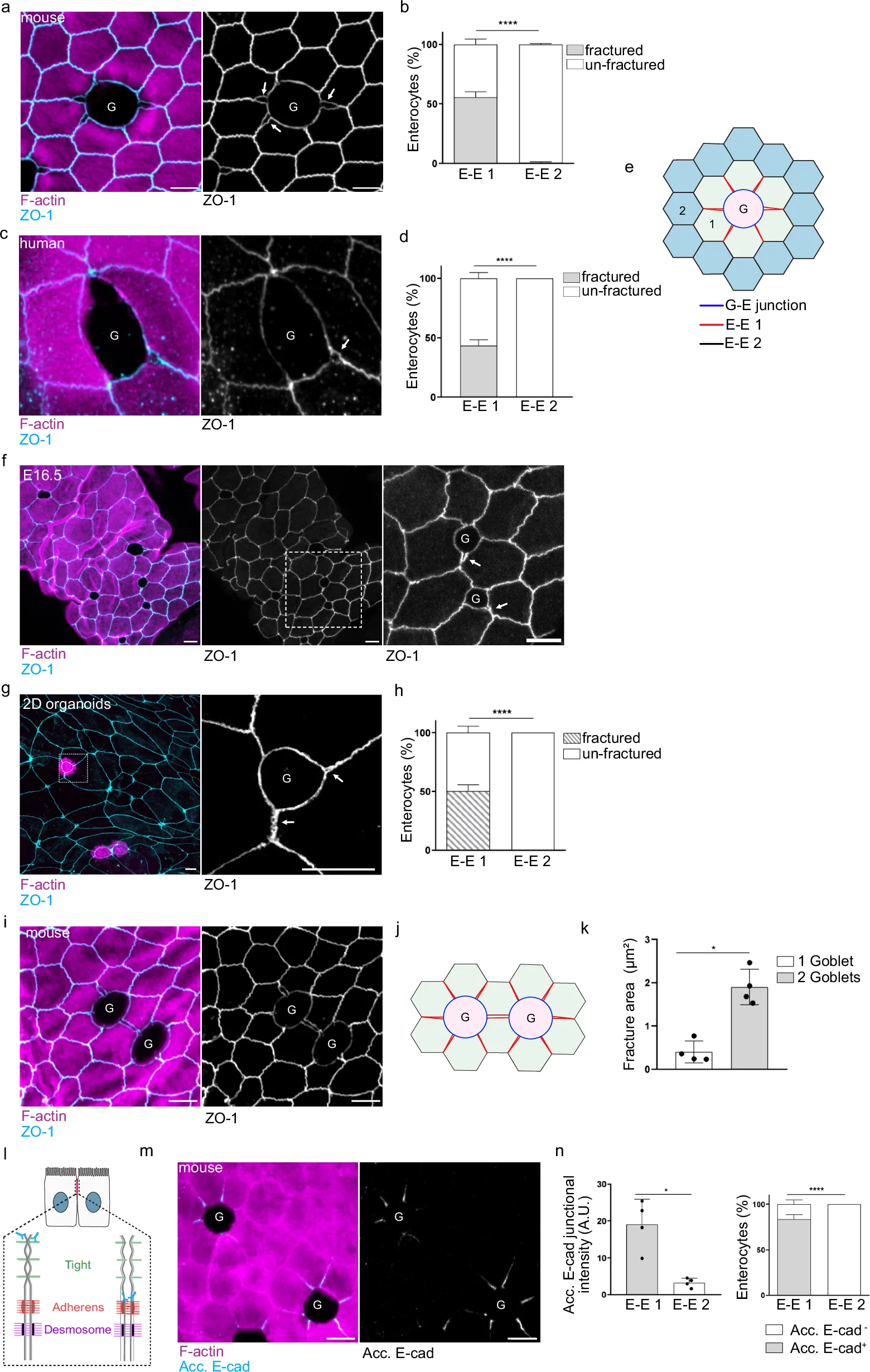
Goblet cells are associated with TJ fractures between adjacent enterocytes under homeostatic conditions. **a** Representative *en face* image of murine small intestinal tissue; maximum intensity projection (MIP) (Z range, 2 µm). White arrows indicate fractures in enterocyte–enterocyte 1^st^ neighbor TJs (E–E1). Scale bars, 5 µm. **b** Stacked bar graph; average percentage ± SD of enterocytes associated with fractures (GAFs). Two-sided Multiple *t*-test with FDR correction, ****p < 0.000001; *n* = 4 biological replicates. **c** Representative *en face* image of human small intestinal tissue; MIP (Z range, 2.2 µm). The white arrow indicates fractures in the E–E1 junctions. Scale bars, 5 µm. **d** Stacked bar graph; average percentage ± SD of GAFs. Two-sided multiple *t*-test with FDR correction, ****p < 0.000001; *n* = 4 biological replicates. **e** Schematic representation; fractures are present in E–E1 (red) and absent in G–E (blue) and 2^nd^ neighbor E–E TJs (E–E2, black). **f** Representative images of E16.5 murine intestine. White arrows indicate GAFs; *n* = 4 embryos. Scale bars, 5 µm. **g** Representative images of 2D organoids; WGA (magenta), ZO-1 (cyan). White arrows indicate GAFs; MIP (Z range, 3.8 µm). Scale bar, 10 µm. The boxed region is shown in higher magnification. Scale bar, 5 µm. **h** Stacked bar graph; average percentage ± SD of GAFs. Two-sided multiple *t*-test with FDR correction, ****p = 0.000081; *n* = 4 biological replicates. **i** Representative image of two GCs in close proximity; MIP (Z range, 2.8 µm). ZO-1 (cyan); F-actin (magenta). Scale bars, 5 µm. **j** Schematic representation showing the cumulative effect of 2 GCs on fracturing. **k** Bar chart; average fracture area ± SD between 2 GCs. Two-tailed Mann–Whitney test, *p = 0.0286; *n* = 4 biological replicates. **l** Accessible E-cadherin staining; schematic representation. **m** Representative image of murine tissue; accessible E-cadherin (cyan) and F-actin (magenta); MIP (Z range, 3.18 µm). Scale bars, 5 µm. **n** Left: Bar chart; average accessible E-cadherin junctional intensity ± SD; Two-tailed Mann–Whitney test, *p = 0.0286; *n* = 4 biological replicates. Right: stacked bar graph; the average percentage of accessible E-cadherin-positive cells. Two-sided multiple *t*-test with FDR correction, ****p = 0.000067; *n* = 3 biological replicates. Acc. E-cad, accessible E-cadherin.

To investigate how GCs are integrated in the small intestinal epithelium, we first assessed TJs by immunofluorescence (ZO-1, occludin) on thick sections of adult murine intestine (jejunum), enabling high-resolution 3D imaging of the villus apical surface *en face* (Fig. 1a; Supplementary Fig. 1a). GCs are easily identified due to their round apical shape and absence of an F-actin-rich brush border, in contrast to polygonal enterocytes; their identity was validated by staining for mucins (Supplementary Fig. 1b). We observed that GCs are frequently (>50%) and significantly associated with fractures (Fig. 1a, b; Supplementary Fig. 1a, arrows) in adjacent (1^st^ neighbor) enterocyte–enterocyte (E–E1) TJs. In contrast, we rarely observed (0.7%) fractures in E–E TJs non-adjacent to GCs (2^nd^ neighbors or farther) (Fig. 1a, b, e). The fractures are wider proximal to the GCs, at the goblet–enterocyte–enterocyte (G–E–E) tricellular junctions and become gradually narrower away from the GC along the E–E bicellular TJs (Supplementary Fig. 1c). In addition, we assessed the presence of fractures at other junctional complexes – at AJ level, fractures were present but ≈3 times less frequent (18%) than at TJs, and no fractures were detected at the level of desmosomes (Supplementary Fig. 1d, e). Importantly, we also observed fractures in healthy regions of human small intestinal biopsies (ileum) with the same frequency (Fig. 1c, d; Supplementary Fig. 1f), suggesting that this phenomenon is conserved between mouse and human.

We asked whether fractures could be the result of extrinsic forces (e.g., luminal shear stress due to digesta flow) or interaction with gut microbiota or immune cells. Remarkably, we found GC-associated fractures in murine embryonic villi (E16.5 embryos, early upon the emergence of villi) (Fig. 1f), where digesta, immune system, and microbiota are not yet present. To further investigate the influence of extrinsic cues, we turned to the murine small intestinal 2D organoids model, which comprises only epithelial cells with functional GCs and mucin granules, and whose accessible apical surface enables high-resolution imaging of the TJs^20^. Similar to what we observed in vivo, in the villus-like regions, 50% of goblet cells were associated with TJ fractures (Fig. 1g, h). Together, these findings suggest that GC-associated fracturing is epithelium-autonomous, arising from intrinsic properties of the epithelial monolayer. We termed this phenomenon “Goblet cell-Associated Fractures” (GAFs).

Interestingly, we observed an increase in GAF area (five-fold increase) when GCs were in close proximity to each other, i.e., separated by only one row of neighboring cells (Fig. 1i–k); this suggests a cumulative effect. To determine whether GAFs represent a breach in the epithelial barrier, we assessed the luminal accessibility of E-cadherin, which is normally inaccessible to antibodies without prior permeabilization. Using a sequential staining approach, tissues were first incubated with an antibody targeting the extracellular domain of E-cadherin before permeabilization, followed by F-actin staining after permeabilization (Fig. 1l)^21^. The “accessible” E-cadherin signal was detected only in GC-adjacent E–E junctions, with over 80% of GCs associated with accessible E-cadherin staining (Fig. 1m, n), indicating that TJs around GCs are indeed breached. Taken together, these data suggest that GCs locally disrupt junctional integrity in the gut epithelium under homeostatic conditions.

### Goblet cells mechanically deform the neighboring enterocytes

**Fig. 2:**
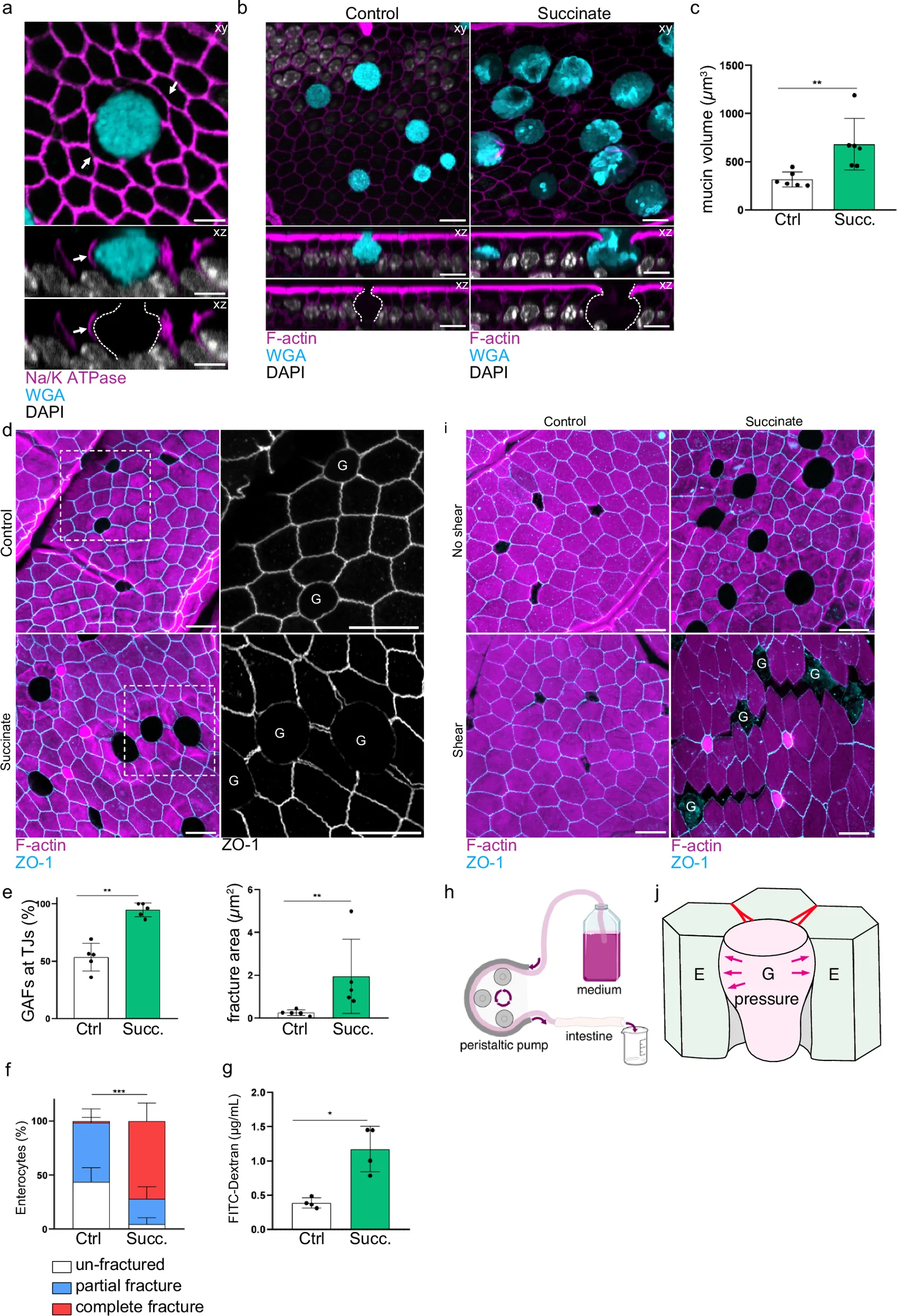
Goblet cells mechanically compress the neighboring enterocytes, leading to their deformation and junctional fractures. **a** Representative orthogonal (xy and xz) confocal images from thick intestinal sections. White arrows are showing lateral deformation of GC-adjacent enterocytes. Scale bars, 5 µm. Similar observations were made in at least five independent experiments. **b** Representative xy and xz images of control and succinate tissue showing increased mucin volume and deformation of goblet-adjacent enterocytes. Scale bars, 10 µm. **c** Bar chart displaying average mucin volume ± SD. Two-tailed Mann–Whitney test, **p = 0.0022; *n* = 6 biological replicates. **d** Representative images from control and succinate tissues. Boxed regions are shown in higher magnification. MIP (Z range, 4.8 µm, 2.7 µm). Scale bars, 10 µm. **e** Left: Bar charts displaying average percentage of GAFs at TJs level ± SD. Two-tailed Mann–Whitney test, **p = 0.0079. Right: Bar chart showing average fracture area ± SD. Two-tailed Mann–Whitney test, **p = 0.0079; *n* = 5 biological replicates. **f** Stacked bar graph showing the average percentage of unfractured, partially fractured, and fully fractured TJs ± SD at E–E1 junctions. Two-sided Multiple *t*-test with FDR correction, ***p = 0.0004; *n* = 4 experiments. **g** Bar charts displaying average intestinal permeability ± SD. Two-tailed Mann–Whitney test, *p = 0.0286; *n* = 4 biological replicates. **h** Schematic representation of the experimental procedure to apply shear stress ex vivo on the intestinal epithelium. **i** Representative images of control and succinate tissues submitted to luminal shear stress; MIP (Z range, 3.2–4.8 µm). Scale bars, 10 µm; Similar results were observed in at least four independent biological replicates. **j** Schematic representation showing hypothetical pushing forces (pink arrows) emanating from the GC, straining E–E junctions, leading to fracturing. Ctrl control, Succ. succinate.

Unlike their slender enterocyte neighbors, GCs have a bulky body filled with mucin granules, which deforms the lateral walls of adjacent enterocytes (Fig. 2a, Supplementary Movie 1). Furthermore, super-resolution imaging of desmoplakin, a marker of desmosomal outer dense plaques, revealed a significant and consistent two-fold decrease in intra-desmosomal space at the G–E junctions compared to E–E junctions (Supplementary Fig. 2a), suggesting mechanical compression exerted by goblet cells. Given this, we then explored whether GAF formation is due to the mechanical interactions between GCs and their neighboring enterocytes.

From a physical perspective, the stereotypical outward curvature of the G–E junction must arise from GCs having a higher pressure than enterocytes, and such pressure difference should be balanced by an interfacial tensile force at the G–E boundary (see Supplementary Method - Theoretical Modeling for details). The tensile state of the G–E boundary is also evident from its smoothness, which is notably different from the wiggliness displayed by E–E TJs (Supplementary Fig. 2b). Previous studies indicate that the G–E interfacial tension (i.e., the force at the G–E boundary) could act as a “peeling force” on the E–E interface, causing debonding^22,23^. Minimal physical modeling of the E–E interface mechanics indeed predicts junctional fracture above a critical G–E interfacial tension (see Supplementary Fig. 2c–f and Supplementary Method - Theoretical Modeling - Section 1 for details). Overall, this led us to hypothesize that GCs exert mechanical forces on their neighbors: the inner pressure within GCs induces a tensile force at the G–E boundary, peeling E–E junctions, and causing junctional fracture.

To test this hypothesis directly, we manipulated GC pressure and the corresponding G–E interfacial force by treating mice with succinate, a microbial metabolite which has been proposed to induce GC hypertrophy (an increase in intracellular GC mucin volume) and hyperplasia (an increase in the number of GCs)^24^. We confirmed that the succinate treatment led to a significant increase in GC mucin volume (control: 317.04 µm^3^; succinate: 681.57 µm^3^) (Fig. 2b, c, Supplementary Movie 2, 3), but not GC hyperplasia, as GC numbers remained similar (Supplementary Fig. 2g, h). Hypertrophic GCs were associated with significantly increased fracturing in adjacent E–E tight junctions, showing a three-fold increase in GAF area (control: 0.42 µm^2^; succinate: 1.29 µm^2^) and a nearly two-fold increase in GAF frequency (control: 53%; succinate: 95%) (Fig. 2d, e). Despite the increase in fracturing, ZO-1 junctional intensity remained unchanged, suggesting that fractures arise from mechanical perturbations imposed by GC pressure rather than from an underlying structural fragility of the junctions due to decreased ZO-1 (Supplementary Fig. 2i). These results were recapitulated in organoid monolayers, where we induced GC hyperplasia and hypertrophy by inhibiting the Notch and Wnt pathways^25^ (Supplementary Fig. 3a–f).

Additionally, GC hypertrophy upon succinate treatment resulted in an increase in complete (vertex-to-vertex) GAFs (Fig. 2f), consistent with the model’s prediction of complete junctional fracture once the GC pressure threshold is exceeded. Fractures also appeared to extend deeper, as indicated by the increased number of fractures at the level of the AJs and the appearance of fractures at the desmosomes (Supplementary Fig. 3g–j). Importantly, this larger and deeper fracturing was accompanied by a significant increase in intestinal permeability in vivo (Fig. 2g), suggesting that GC hypertrophy severely weakens epithelial barrier integrity. At the same time, no increase in inflammatory markers or weight loss was observed (Supplementary Fig. 4a, b), suggesting that succinate treatment does not induce immune-mediated tissue damage. The observed increase in fracturing and permeability is therefore likely to be driven by epithelial mechanics rather than disease. To test whether increased fracturing observed with GC hypertrophy further compromises tissue integrity under extrinsic mechanical stress, we applied a defined flow through the intestinal lumen ex vivo using a peristaltic pump system (shear stress estimated at 1 Pa) (Fig. 2h). This shear stress challenge resulted in a striking increase in fractures emanating from GCs in tissues from succinate-treated mice, with fractures extending further (≥3 neighboring cells) compared to control tissue, which remained unaffected (Fig. 2i; Supplementary Fig. 4c). In addition to shear stress, the applied flow also generates a modest normal (luminal) pressure component (~154 Pa), which did not alter enterocyte apical surface area (Supplementary Fig. 4d). Taken together, these data suggest that GCs exert pressure on neighboring enterocytes, leading to junctional ruptures (Fig. 2j), which can be further exacerbated by increased GC volume (goblet hypertrophy). Importantly, we found that this leads to functional consequences, notably an increase in intestinal permeability and heightened susceptibility to mechanical stress from the environment.

### Active response of the tissue to goblet cell pressure

**Fig. 3:**
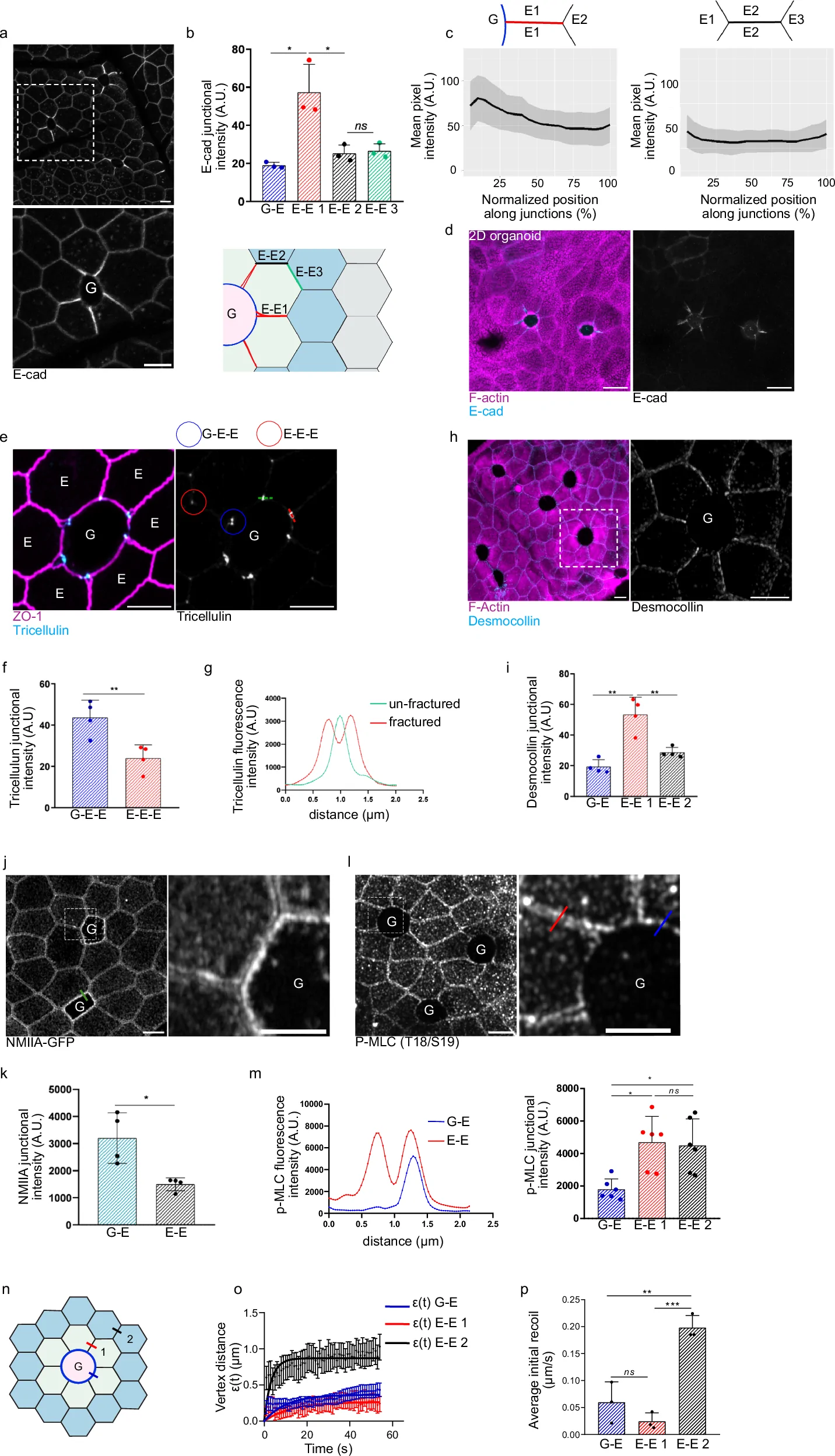
Active mechanoadaptation of the tissue to goblet cell pressure. **a** Representative *en face* image; boxed region shows higher magnification; MIP (Z range, 1.8 µm). Scale bars, 5 µm. **b** Bar chart showing average junctional intensity ± SD. Two-tailed Multiple *t*-test with FDR correction, ns non-significant, *p = 0.0108; *n* = 3 biological replicates. **c** Average line intensity profiles for junctional E-cadherin E–E1 (left) and E–E2 (right) along rescaled E–E junctions. **d** Representative images of 2D organoid monolayers; MIP (Z range, 2.7 µm). Scale bars, 10 µm. **e** Representative *en face* image; G–E–E are encircled in blue and E–E–E in red; MIP (Z range, 1.4 µm). Scale bars, 5 µm. **f** Bar charts showing average intensity in G–E–E or E–E–E TCJs ± SD. Two-tailed *t*-test. *p = 0.0100; *n* = 4 biological replicates. **g** Line intensity profiles for tricellulin at fractured (red) and unfractured (green) tricellular junctions, corresponding to **e**. **h** Representative *en face* image; MIP (Z range, 1.5 µm). Boxed region shows higher magnification. Scale bars, 5 µm. **i** Bar chart displaying average junctional intensity ± SD. Two-tailed Multiple *t*-test with FDR correction, **p = 0.0013; *n* = 4 biological replicates. **j** Representative confocal image of live tissue showing apical NMIIA-GFP signal. MIP (Z range, 1.2 µm). Scale bar, 5 µm. The boxed region shows higher magnification. Scale bar, 2 µm. **k** Bar chart displaying average junctional intensity ± SD. Two-tailed Mann–Whitney test, *p = 0.0286; *n *= 4 biological replicates. **l** Representative image showing apical pMLC signal. Scale bar, 5 µm. The boxed region shows higher magnification. Scale bar, 2 µm. **m** Left: pMLC line-intensity profiles corresponding to the blue G–E and red E–E lines shown in **I**. Right: Bar chart displaying average pMLC junctional intensity ± SD. Kruskal–Wallis test, ns non-significant; *p = 0.0175, *n* = 6 biological replicates. **n** Laser cut locations. **o** Vertex-vertex distance ε(*t*) plot in function of time for G–E (red), E–E1 (green), and E–E2 (blue) AJs; *n* = 3 biological replicates. **p** Bar chart showing average initial recoil speed ± SD for G–E, E–E1, and E–E2 AJs. Two-sided Multiple *t*-test with FDR correction, ns non-significant; **p = 0.0023; ***p = 0.0007; *n* = 3 biological replicates.

Cellular junctions are mechanoresponsive. This response involves different junctional components at different levels^6,26^. E-cadherin, a key marker of AJs, is known to respond to mechanical stress by clustering^11^. Interestingly, we found a striking increase in E-cadherin junctional levels in GC-adjacent E–E AJs compared to non-adjacent AJs (Fig. 3a, b). Moreover, E-cadherin signal levels were heterogeneous along the junctions, progressively increasing toward the goblet cell (Fig. 3c), a pattern also observed in 2D organoids (Fig. 3d). This accumulation prompted us to investigate whether it indicated higher strain near GCs. To test this, we analyzed the distribution of tricellulin, a marker of tricellular junctions (TCJs), which are associated with tension hotspots and mechanical failure points^27,28^. Notably, tricellulin levels were elevated at G–E–E TCJs compared to E–E–E TCJs (Fig. 3e, f). Moreover, the tricellulin signal at the GAFs was disorganized, exhibiting multiple puncta, in contrast to the single puncta observed at unfractured TCJs (Fig. 3e, g), supporting the hypothesis that fractures initiate at the G–E–E TCJ and propagate toward the E–E bicellular junction (Supplementary Fig. 1c). At the level of desmosomes, we found a 2.8-fold increase in desmocollin – a desmosomal cadherin^29^ – in GC-adjacent E–E desmosomes (Fig. 3h, i), suggesting local mechanoadaptation of desmosomes.

Because junctional fracturing often correlates with locally increased active stresses generated by actomyosin contractility^30^, and given the increased E-cadherin signal we observed (Fig. 3a), indicative of local mechanical strain, we next investigated whether and how actomyosin contractility contributes to GAFs.

To determine whether there are differences in contractility between GCs and their neighboring enterocytes, we first assessed the levels of non-muscle myosin IIA (NMIIA) in the apical actomyosin belts using live imaging of gut explants derived from mice expressing GFP-tagged endogenous NMIIA^31^. We found that GCs exhibited higher NMIIA levels in their apical belts compared to the myosin belts of enterocytes (Fig. 3j, k). However, phospho-myosin light chain (T18/S19) (pMLC) staining, a marker of active myosin, was not detectable in the apical belts of GCs compared to the junctions of neighboring enterocytes (Fig. 3l, m), suggesting lower contractility. To functionally assess tensile forces around goblet cells, we performed spatially resolved laser ablation of individual G–E junctions and surrounding first- and second-neighbor enterocyte–enterocyte junctions (E–E1 and E–E2) ex vivo in gut explants^5^ (Fig. 3n). Junctional recoil velocity was approximately 10-fold higher at E–E2 junctions than at either G–E or E–E1 junctions, while G–E and E–E1 did not differ significantly (Fig. 3o, p; Supplementary Movies 4–6). Because laser ablation preferentially reports the release of pre-existing tensile stress along junctions, these results indicate that G–E interfaces are not under elevated junctional tension, even though GCs exert local pressure on their neighbors. Consistent with this, pMLC levels were markedly lower at G–E junctions than at E–E1 and E–E2 junctions, supporting reduced myosin activity at G–E interfaces. Notably, E–E1 and E–E2 junctions exhibited comparable pMLC levels despite their strikingly different recoil velocities, indicating that differences in recoil cannot be fully explained by gradients in actomyosin-generated tension alone. Together, these observations suggest that tensile contractility is not the primary driver of GAF formation.

Altogether, our results show that junctional protein accumulation at TCJs, AJs, and desmosomes in enterocytes correlates with their proximity to GCs and is not driven by increased tensile stress. We therefore propose that the tissue actively undergoes mechanoadaptation in response to compressive forces, mitigating the stresses generated by goblet cell–induced compression and junctional fracturing.

### Fracturing depends on the mechanical properties of neighboring cells

Beyond its role in contractility, myosin II crosslinking of cortical actin filaments is critical for the mechanical properties of cells and junctions (e.g., rigidity), which influence cell shape and junctional strength^32–34^. Recent theoretical studies incorporating fracture into vertex models further support the idea that changes in tissue deformability modulate susceptibility to local fractures^35,36^.

**Fig. 4:**
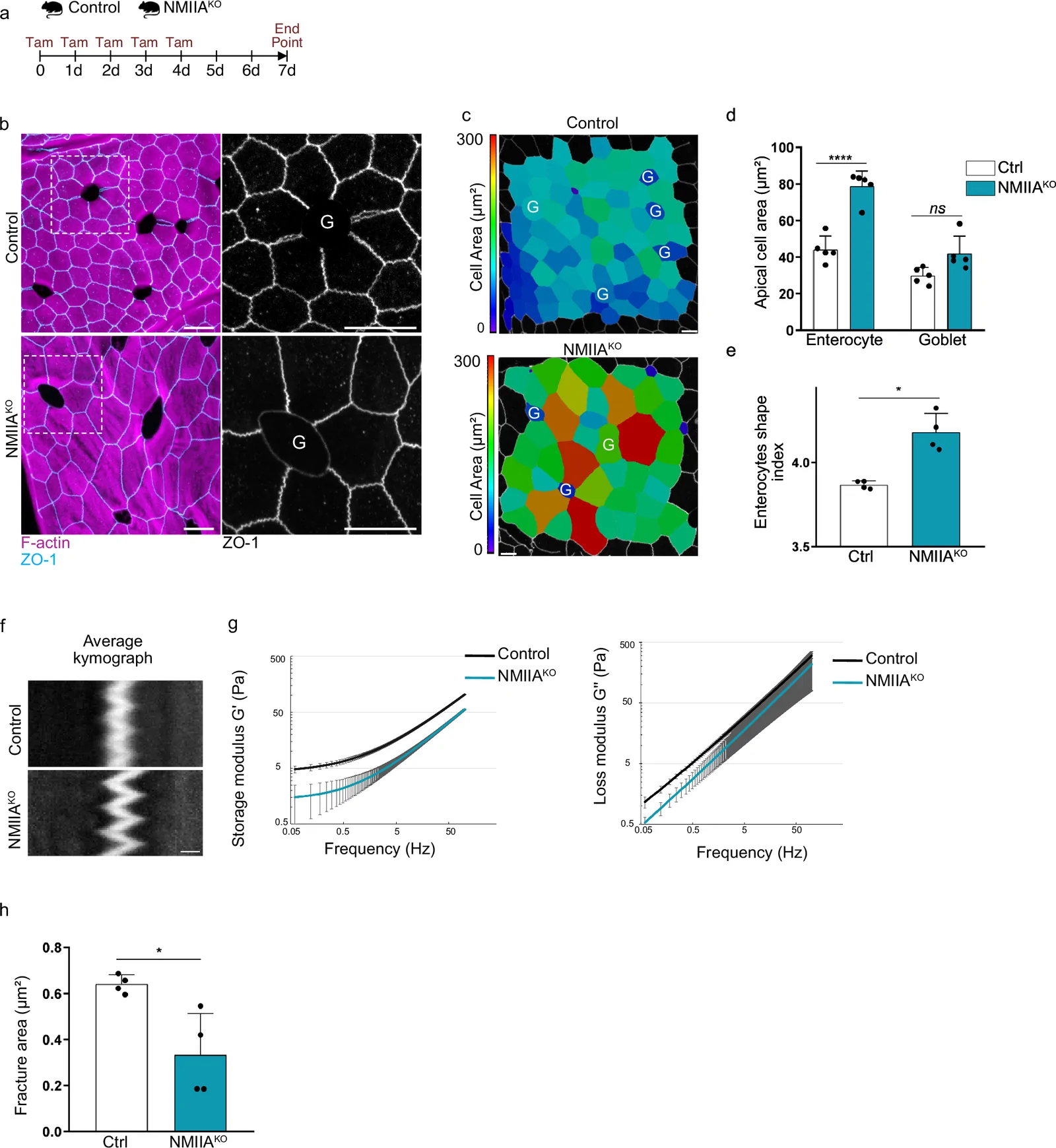
Goblet cell-associated fracturing depends on global tissue mechanical properties. **a** Schematic representation of the experimental procedure for NMIIA-KO mice; Tam tamoxifen. **b** Representative images from control and NMIIA-KO mice; MIP (Z range, 4.5 µm and 3.8 µm). Boxed regions are shown in higher magnification. Scale bars, 10 µm. **c** Representative heatmap images displaying apical cell area in control and NMIIA^KO^ mice. Scale bars, 5 µm. **d** Bar chart displaying average apical cell area ± SD. Two-tailed Multiple *t*-test with FDR correction, ns: non-significant; ****p < 0.00001; *n* = 5 biological replicates. **e** Bar chart displaying average enterocyte shape index ± SD. Two-tailed Mann–Whitney test. *p = 0.0286; *n* = 4 biological replicates. **f** Average kymograph of lipid droplet displacement; scale bar, 1 µm. Each kymograph was generated using a 15 s movie. **g** Frequency-dependent storage modulus (G′, left) and loss modulus (G″, right) ± SD derived from optical tweezers measurements in control and NMIIA-KO tissues. *n* = 3 biological replicates. **h** Bar chart showing average fracture area ± SD. Two-tailed Mann–Whitney test. *p = 0.0286; *n* = 4 biological replicates.

We therefore hypothesized that epithelial myosin II could significantly influence the rigidity/deformability of the tissue and thus the formation of GAFs. We tested this using an inducible, intestinal epithelium-specific NMIIA knockout mouse model (Villin-CreERT2; *Myh9*^fl/fl^, hereafter referred to as NMIIA-KO) (Fig. 4a; Supplementary Fig. 5a).

After NMIIA depletion, enterocytes no longer formed regular pentagonal or hexagonal arrays but became floppy and disorganized (Fig. 4b). Given previous work showing that cell shape metrics tightly correlate with tissue rheology^37–39^, we reasoned that junctional fracture may not be solely a local mechanical event, but may instead depend on tissue-scale mechanical properties. Consistent with theoretical studies^35,36^, if GC-adjacent enterocytes are floppy, GC-induced forces may be dissipated more effectively, thereby alleviating GAFs.

To test this hypothesis, we first segmented control and NMIIA-KO tissues and quantified apical cell geometry. NMIIA-KO tissue exhibited increased apical cell area and a higher cell shape index compared to controls (control: 3.86; NMIIA-KO: 4.18) (Fig. 4b–e), consistent with a transition toward a more deformable epithelial state. We further corroborated this interpretation by directly measuring cytoplasmic viscoelastic properties using optical tweezers^40^, which revealed that NMIIA-KO tissue is mechanically softer than control tissue at the intracellular level (Fig. 4f, g). Optical tweezers–based tracking of endogenous intracellular lipid droplet displacement shows larger oscillatory amplitudes in NMIIA-KO tissue compared to control, as illustrated in the representative kymographs (Fig. 4f), consistent with reduced cytoplasmic mechanical confinement. Active microrheology using optical tweezers enabled reconstruction of the frequency-dependent storage (G′) and loss (G″) moduli (Fig. 4g), both of which were reduced in NMIIA-KO tissue, indicating a decrease in intracellular viscoelastic stiffness. These findings demonstrate altered cytoplasmic mechanical properties and increased deformability in NMIIA-KO tissue. Consistent with this mechanical shift, NMIIA depletion resulted in a significant reduction in GAF area (control: 0.64 µm^2^; NMIIA-KO: 0.33 µm^2^) (Fig. 4h), and decreased E-cadherin enrichment (Supplementary Fig. 5b, c), while GC mucin volume remained unchanged (Supplementary Fig. 5d).

To directly disentangle the effect of NMIIA loss in GCs versus enterocytes, we assessed fracture formation in Muc2-CreERT2; *Myh9*^fl/fl^ mice, which enable inducible, GC-specific depletion of NMIIA while preserving NMIIA expression in enterocytes (Supplementary Fig. 6a). GC-specific NMIIA depletion did not alter the number or area of GAFs (Supplementary Fig. 6b, c), indicating that NMIIA activity within GCs is not required for fracture formation. These results support the conclusion that fracture formation primarily depends on the rheological properties of the surrounding enterocyte network, regulated by enterocyte myosin II activity, rather than on contractility within goblet cells themselves.

Altogether, our results show that enterocyte rheology governed by myosin II is a key determinant of GAF formation.

This result, however, contradicted the peeling model for E–E interfaces (Supplementary Fig. 2c), which predicted that a decrease in junction rigidity would facilitate fracture as junctions become more susceptible to GC-induced deformation (see Supplementary Fig. 2e, f and Supplementary Method - Theoretical Modeling - Section 1 for discussion). A possible explanation for this discrepancy is that the peeling model considers only a single E–E junction, whereas tissue-level models emphasize how global cell shape and packing influence epithelial mechanics.

**Fig. 5:**
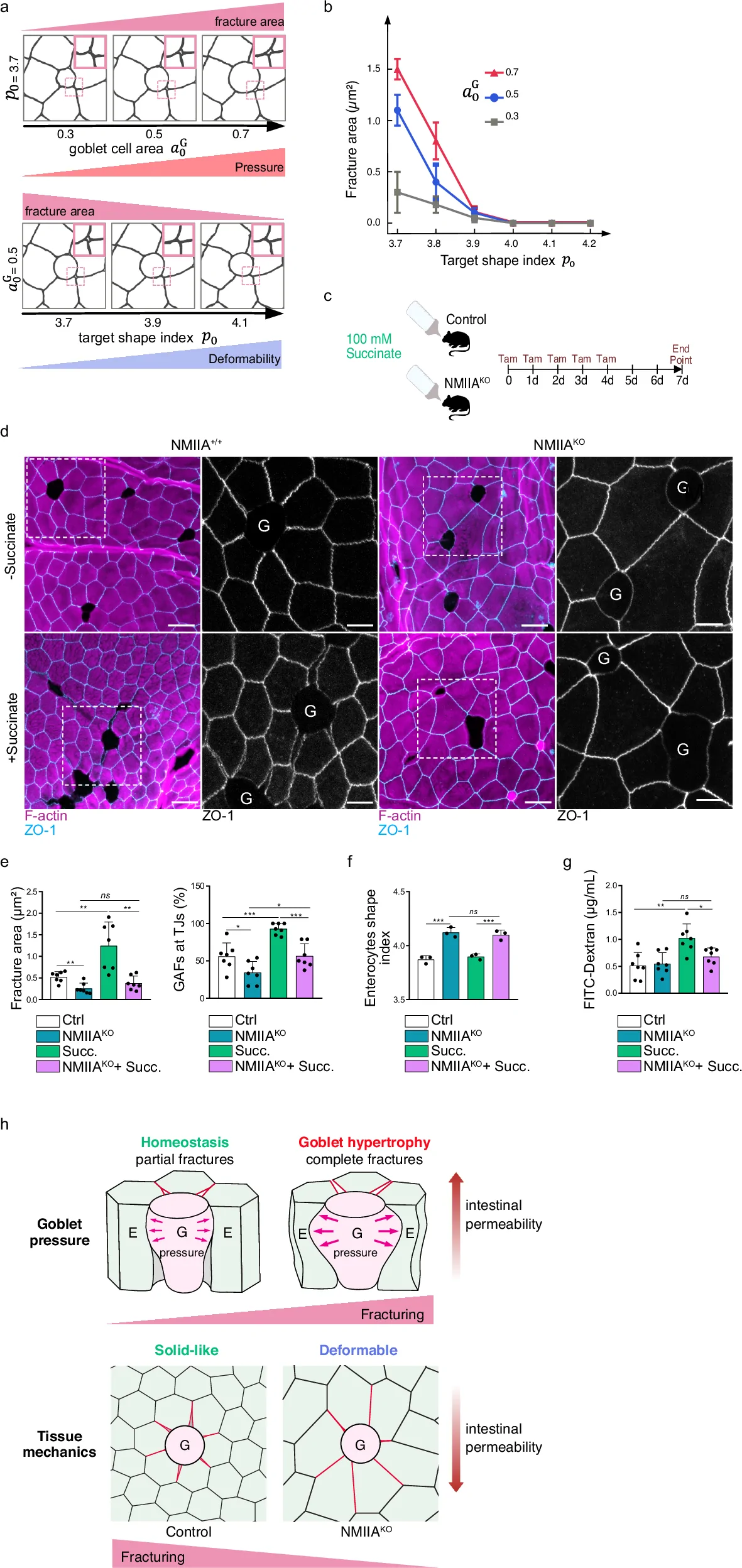
Increased enterocyte deformability prevents hypertrophy-induced fracturing. **a** Simulations of the tissue mechanics model with two main parameters: the goblet cell area ${a}_{0}^{{{{\rm{G}}}}}$ and the shape index of enterocytes ${p}_{0}$ (relevant to tissue rheology). The model predicts that GAFs increase with the goblet cell area ${a}_{0}^{{{{\rm{G}}}}}$ (cell shape index *p*_0_ = 3.7, top panel), but decrease with the shape index ${p}_{0}$ (goblet cell area ${a}_{0}^{{{{\rm{G}}}}}=0.5$, bottom panel). **b** Predicted fracture area as a function of goblet cell area $\left({a}_{0}^{{{{\rm{G}}}}}\right)$ and shape index of enterocytes $\left({p}_{0}\right)$; mean ± SD from 3 independent simulations. **c** Schematic representation of succinate treatments in control (NMIIA^+/+^) or NMIIA-KO mice. **d** Representative *en face* images from control (NMIIA^+/+^) and NMIIA-KO mice, with and without succinate treatment. Scale bars, 10 µm. Boxed regions are shown in higher magnification. MIP (Z range, 3.0–4.5 µm). Scale bars, 5 µm. **e** Bar charts displaying average fracture area ± SD (left). Two-tailed Multiple *t*-test with FDR correction, ns non-significant; **p = 0.0031; and average percentage of GAFs ± SD (right), according to the conditions. Two-tailed Multiple *t*-test with FDR correction, ns non-significant, *p = 0.0477; ***p = 0.0008. *n* = 7 biological replicates. **f** Bar chart displaying average enterocyte shape index ± SD. Two-sided Multiple *t*-test with FDR correction. ***p = 0.0002; *n* = 3 biological replicates. **g** Bar chart showing average intestinal permeability ± SD. Two-sided Multiple *t*-test with FDR correction; ns non-significant, *p = 0.0477; **p = 0.0014; *n* = 7 biological replicates. Succ. succinate. **h** Goblet cells disrupt the intestinal barrier by inducing fractures in the tight junctions of neighboring enterocytes through a two-tiered mechanical process. Goblet cells exert compressive forces on adjacent enterocytes, leading to junctional fracturing that increases with goblet cell hypertrophy and results in elevated intestinal permeability. The susceptibility of enterocytes to fracturing depends on their rheological properties, which are controlled by myosin IIA: increased tissue deformability protects against junctional fracture and prevents hypertrophy-induced hyperpermeability.

To explore this hypothesis, we developed an extended vertex model that retained the key physical components of the classical vertex model^35,36,38,39,41,42^ – namely, the existence of a preferred cell shape and area – while allowing for complex junctional deformations and ruptures, which were crucial for Modeling GC and GAF shapes (for more details, see Supplementary Method - Theoretical Modeling Section 2 and Supplementary Fig. 7a). We found that the presence of GAFs was governed by two key parameters (Fig. 5a): GC size/pressure ${a}_{0}^{{{{\rm{G}}}}}$, as in the previous model, and tissue rheology, which can be inferred from the “cell shape index” ${p}_{0}$ (i.e., the cellular perimeter-to-area ratio)^37–39^. Consistent with our experimental data, as the shape index increases, the tissue becomes more deformable^41^, which prevents the formation of GAFs (Fig. 5a, b).

Together, these findings indicate that epithelial fracturing depends not only on goblet cell–induced pressure but also on tissue-scale cell shape and mechanical properties regulated by myosin II activity.

To further test model predictions and determine whether myosin II depletion could prevent the increased fracturing and barrier breaching observed with GC volume increase (Fig. 2), we induced GC hypertrophy in vivo in NMIIA-KO mice (Fig. 5c) and in blebbistatin-treated 2D organoids (Supplementary Fig. 8a). Hypertrophy-induced GAFs were significantly reduced both in vivo in NMIIA-KO and in vitro (Fig. 5d, e; Supplementary Fig. 8a–d), including deeper fractures at AJs and desmosomes (Supplementary Fig. 8e–h). Consistent with our previous findings, NMIIA-KO enterocytes, treated or not with succinate, displayed an increased cell shape index compared to control or succinate-treated epithelium (Fig. 5f), further emphasizing the importance of enterocyte rheology in the fracturing process. Importantly, the GC hypertrophy-associated increase in intestinal permeability observed in vivo was significantly reduced in NMIIA-KO mice (Fig. 5g). Overall, our results indicate that GAF formation depends on both goblet cells and their immediate neighbors – a two-component system in which the goblet cell body pushes and deforms neighboring enterocytes, straining and fracturing their junctions, which depends on tissue rheology controlled by myosin II (Fig. 5h).

## Discussion

Here, we show that under homeostatic conditions in vivo, intestinal goblet cells (GCs) exert local mechanical compression on adjacent enterocytes due to their bulky shape and volumetric expansion, resulting in fracture of junctions between enterocytes, as a function of enterocyte mechanics controlled by myosin II.

The integrity of the intestinal epithelial barrier is critical for maintaining homeostasis, and its disruption can trigger pathologies such as inflammatory diseases (IBD) and tumorigenesis^43^. The barrier function of the intestinal epithelium is maintained by adhesive junctional complexes, particularly TJs, which are the primary physical determinant of the epithelial barrier^8^, assumed to form a continuous, uninterrupted seal between adjacent cells, blocking the passage of bacterial toxins and pathogens into the underlying tissues^43^. However, most studies of epithelial cell junctions have focused predominantly on cell lines or single “main” cell types, such as enterocytes, using thin histology sections. In contrast, employing thick tissue sections and high-resolution 3D confocal microscopy, we found that approximately 50% of GCs are associated with fractures at the TJ level. This GC-associated junctional breaching is even more prevalent than what we can detect by imaging TJ markers, as revealed by the E-cadherin accessibility assay, which showed 80% of GC-adjacent enterocytes with breached TJs. Junctional fracturing in epithelial tissues has previously been observed in cultured monolayers subjected to extrinsic stretch^15^, in placozoans under high motile stress^36^, and during hydraulic fracturing of blastocysts^44^. However, goblet cell-associated fractures represent the first example of an adult epithelium undergoing self-induced rupture under homeostatic conditions.

While the epithelial integrity and mechano-responsiveness of individual junction types have been relatively well studied, their comparative adhesive strength and resistance to mechanical stress in vivo remain unclear. Interestingly, we found that TJs fracture more frequently (50%) than AJs (18%), while no fractures were observed at the desmosome level under homeostatic conditions. This suggests that TJs are more fragile and less resistant to mechanical stress than the underlying junctions. Notably, fractures predominantly originate at tricellular junctions (TCJs) at the G–E–E interface, before propagating along the E–E bicellular junction. These results are consistent with observations that TCJs are points of high tension in the tissue and may represent “weak spots” in the epithelium that are more prone to fracturing^27^. Remarkably, we found that enterocytes accumulate transmembrane proteins E-cadherin and desmocollin in response to the presence of GCs, suggesting junctional reinforcement, a concept extensively reviewed previously^11^. This accumulation of junctional proteins may serve to increase junctional strength and prevent further rupture.

Junctional reinforcement has been described as a response to increased tension generated by intrinsic cellular contractility in developing embryos and cultured epithelial monolayers – as previously reviewed^14^, suggesting that GC contractility may play a role. However, the prominent apical myosin ring in GCs does not appear to be contractile, leading us to speculate that it has a more passive, scaffolding role in containing mucin granules within the cell. In addition, we found that GAFs depend on global tissue mechanical properties, which we manipulated by perturbing myosin II. Reduced myosin expression or activity resulted in a more deformable tissue that was less prone to rupture. Such mechanical softening could act by preventing stress accumulation in the tissue by allowing the cells to adjust their shape/size and position, and thus adapt to abnormal mechanical forces, protecting the tissue from physical damage. These findings are in line with recent in vitro experimental work^15^ and theoretical studies of rupture in epithelia^35^, which show that myosin and the associated mechanical state of the tissue play a critical role in fracturing processes.

The presence of GCs is necessary for fractures to occur, as no fractures were detected in regions within the epithelium without GCs. Our results indicate that fractures are promoted by the volume of GCs and the pressure they exert on neighboring enterocytes. In our study, succinate treatment was used to mimic a physiological response to parasitic infection, a condition previously described as inducing GC hyperplasia and hypertrophy, leading to an increase in GC numbers and mucus hypersecretion. GC hyperplasia and hypertrophy were shown to promote parasite clearance through the “weep and sweep” response and thus “protect” the tissue^24,45^. While we did not observe GC hyperplasia in our experiments, possibly due to technical reasons, we observed prominent GC hypertrophy, characterized by a marked increase in intracellular mucin volume. Interestingly, GC hypertrophy was closely associated with a significant increase in fracture size (both area and depth) and number, ultimately leading to increased intestinal permeability, challenging the widely held view that GC hypertrophy is solely protective.

Although increased permeability was observed following succinate treatment, no evidence of inflammation was detected. This is somewhat unexpected, as elevated paracellular permeability is generally associated with enhanced bacterial translocation and inflammatory responses. This suggests that, under homeostatic conditions, the occurrence of GC-associated fractures alone is not sufficient to trigger overt pathology. We hypothesize that in pathological contexts of active infection, pathogen-induced GC hypertrophy may facilitate the translocation of microbes and luminal toxins, ultimately triggering inflammation. Conversely, under homeostatic conditions, compensatory mechanisms such as mucus hypersecretion may counterbalance GC-induced junctional disruption by enhancing microbial entrapment and thereby limiting exposure of the underlying epithelium. However, when luminal shear stress was applied ex vivo, GC hypertrophy severely compromised tissue integrity, creating large intercellular gaps that spanned multiple rows of adjacent cells. Shear stress is physiologically relevant, as it can increase in conditions such as partial intestinal obstruction or luminal narrowing caused by inflammation, fibrosis, strictures, or tumors, where fluid velocity increases. These findings suggest that an increase in GC volume (hypertrophy) is necessary and sufficient to increase fracturing and weaken the intestinal epithelium, making it more vulnerable to mechanical insults, which could further exacerbate damage and trigger inflammation.

GCs have been identified as entry points for *Listeria monocytogenes* invasion and infection^21^, a process that requires luminally accessible E-cadherin. This is consistent with our findings of GAFs and the associated barrier breach, which exposes E-cadherin and could thus facilitate bacterial adhesion and tissue infection. GCs have also been identified as mediators of interactions between the immune system and luminal content through transcytosis-based pathways – the goblet cell-associated antigen passages (GAPs) – that enable the sampling of luminal antigens and their delivery to underlying immune cells^46^. The junctional fractures (GAFs) described here may have a similar function, physically facilitating antigen sampling by immune cells. However, we have also observed GAFs in the embryonic mouse intestine, where the lumen is empty, and neither the immune system nor the microbiota is present. Remarkably, GAFs were observed in both mouse and human intestinal tissue, suggesting they may be conserved across species.

What could be the biological significance of goblet cell-associated fractures under physiological conditions? Fracturing near the goblet–enterocyte interface may serve as a local mechanical dissipation mechanism – a controlled failure to mitigate the risk of larger-scale epithelial disruption. This concept parallels developmental processes, where local deformation and cellular rearrangement dissipate mechanical forces during morphogenesis^7,9,39,47,48^. Consistently, we observed an active tissue response to GC compression, characterized by mechanosensitive reinforcement of junctional adhesion in GC neighbors. Analogous stress-relief phenomena have been observed in a few other biological systems, such as the migratory fracturing in Trichoplax adhaerens^36^, where epithelial rupture is thought to prevent tissue failure under motile stress. At present, this interpretation remains speculative, and further work will be required to determine whether GAFs represent adaptive, repairable events or unavoidable mechanical trade-offs associated with goblet cell function. However, this compensatory response appears insufficient under conditions of GC hypertrophy, where elevated compressive forces exacerbate fracturing and compromise barrier integrity – highlighting the limits of epithelial plasticity under sustained mechanical stress.

Our unexpected results demonstrate that goblet cells challenge the intestinal epithelial barrier by creating structural vulnerabilities that may contribute to intestinal pathologies, including infection, inflammation, and disease progression. In conclusion, we show that the mechanical interplay between goblet cells and neighboring enterocytes promotes junctional fractures, compromising the epithelial barrier. We propose that the mechanical properties of individual cells – both goblet cells and enterocytes – are key factors in maintaining junctional integrity and preserving the intestinal epithelial barrier.

## Methods

### Mice

All mouse husbandry and handling procedures were approved and conducted in accordance with European and national regulations (Protocol Authorization APAFIS #38994-2023062718345638). All mice were kept in the Specific and Opportunistic Pathogen-Free (SOPF) animal facility at the CBI. Comparisons were made between age-matched littermates (8–12 weeks old). As no phenotypic differences were observed between males and females, mice of both sexes were used in all experiments. Animals were monitored daily according to institutional animal welfare guidelines. Wild-type mice and transgenic mouse lines were maintained on a C57BL/6N background.Villin-CreERT2 mice^49^ were crossed with *Myh9*^fl/fl^ mice^50^, obtained from the Mutant Mouse Resource and Research Center (MMRRC; stock 032096-UNC), to generate intestinal epithelial-specific NMIIA knockout mice. *Myh9*^fl/fl^ were also crossed with Muc2-Cre-ERT2 mice^51^ (kindly provided by Dr. C. Lengner (University of Pennsylvania)) to generate Muc2-Cre-ERT2; *Myh9*^fl/fl^ mice. Villin-CreERT2 and NMIIA-GFP^31^ mice were kindly provided by Dr. D. Matić Vignjević (Institut Curie). Mice were euthanized by cervical dislocation under isoflurane anesthesia in accordance with approved ethical protocols.

### Tamoxifen and drug treatments

To induce intestinal epithelium-specific *Myh9* (NMIIA) knockout, Villin-CreERT2; *Myh9*^fl/fl^ and *Myh9*^fl/fl^ (control) mice were intraperitoneally injected with 100 µL of tamoxifen (50 mg/kg; Euromedex SE-S1238) once a day for 5 consecutive days and culled 3 days after the last injection, as indicated. To induce goblet cell-specific *Myh9* knockout, Muc2-Cre-ERT2; *Myh9*^fl/fl^ mice were injected with 100 µL of tamoxifen once a day for 5 consecutive days and culled on the fifth day. For succinate treatment, mice received 100 mM succinate (Sigma, S9637) in drinking water for 7 days^24^, as indicated. For DSS treatment, mice received 3% DSS (Sigma, 42867) in drinking water for 7 days, after which they were culled.

### Antibodies

For immunofluorescence, we used the following antibodies: rat anti-ZO-1 (Millipore, MABT11) at 1/200, rat anti-E-cadherin (Invitrogen, 131900) at 1/200, rabbit anti-occludin (Proteintech, 27260-1-AP) at 1/50, sheep anti-desmocollin-2 (Biotechne, AF7490) at 1/100, Rabbit anti-tricellulin (Invitrogen, 48–8400) at 1/50, mouse anti-desmoplakin (Progen, 651109) at 1/50, rabbit anti-phospho-Myosin Light Chain (P-MLC) (T18/S19) (Cell Signaling, 3674S) at 1/50, rabbit anti-Na/K ATPase (Abcam, ab-76020) at 1/400, rabbit NMIIA (Biolegend, 909801) at 1/100. For human tissue staining, mouse anti-ZO-1 (Invitrogen, 1A12) at 1/100 dilution was used. All secondary antibodies were purchased from Molecular Probes and used at a dilution of 1/200. F-actin was stained using rhodamine- or Alexa-647-conjugated phalloidin (Invitrogen R415, A30107) at 1/200 dilution. Intracellular mucin was stained using WGA-rhodamine (VectorLabs, RL-1022) at 1/100 dilution. DNA was visualized using DAPI (Sigma, D9542) at 5 µg/mL. For western blotting, we used: rabbit anti-NMIIA (Biolegend, 909801) at 1/5000 and rabbit anti-GAPDH (Sigma, G9545) at 1/10,000, followed by horseradish peroxidase-conjugated anti-rabbit (Invitrogen, G21234) as a secondary antibody, at 1/10,000 dilution.

### 3D immunofluorescence staining

Mice were culled as indicated, and the small intestine (jejunum) was isolated, flushed with PBS, cut into 3–5 cm fragments, and fixed in 4% paraformaldehyde (Electron Microscopy Sciences) in PBS for at least 1 h at 37 °C, followed by three washes in PBS. Fixed tissue was hand-cut with a scalpel and permeabilized with 1% Triton X-100 (Sigma) in PBS for 1 h at RT. Slices were then incubated with the primary antibody in PBS overnight at RT under agitation. After three 1-h washes in PBS, secondary antibody was added along with DAPI, and, optionally, WGA and phalloidin, and incubated overnight at RT. Samples were washed three times for 1 h each and mounted on slides using mounting medium (Aqua-Poly/Mount, Polysciences), according to the manufacturer’s instructions.

### Human tissue staining

Human intestinal biopsies were obtained from the CHU Rangueil in Toulouse with informed consent from each patient (MTA 24 134C). The study was conducted in accordance with institutional guidelines and approved by the relevant human research ethics committee. Patients were diagnosed with inflammatory pathologies, and biopsies of the ileum were taken from non-inflamed regions. Tissues were fixed in 4% paraformaldehyde O/N at RT, and washed three times with PBS. Sections were hand-cut with a scalpel, and slices were then permeabilized with 1% Triton X-100 in PBS for 2 h at RT and stained as described above.

### Luminal shear stress

#### Ex vivo perfusion

Mice were sacrificed, and the jejunum portion of the small intestine was collected and cut into ≈5 cm segments. Each intestinal segment was mounted onto a peristaltic pump system via a gavage needle, allowing controlled luminal perfusion. Segments were perfused with Leibovitz’s L-15 medium (1145056, Gibco) at a constant flow rate of 23 mL/min for 5 min. Tissues were then fixed and stained as described above.

#### Simulation of luminal shear stress

Numerical simulations of luminal shear stress were performed using COMSOL Multiphysics and finite element analysis (FEA). The Laminar Flow physics module was used to solve the incompressible Navier-Stokes equations under steady-state conditions. A 2D/3D geometry representative of a murine intestinal segment was constructed to model the internal topography of the jejunum. The model consisted of a 10-mm-long intestinal tube containing 260 villi (500 µm in height), homogeneously distributed along the luminal surface. The working fluid was defined with a density of 1 g/mL and a dynamic viscosity of 10 mPa·s, approximating the properties of the perfusion medium. A volumetric flow rate of 23 mL/min was imposed at the inlet, assuming a uniform velocity profile. Boundary conditions included no-slip at all solid surfaces and zero pressure at the outlet. Shear stress values were extracted from the computed stationary solution.

### Laser ablations of cell junctions

Laser cuts of cell-cell junctions were performed as previously described^5^. Briefly, NMIIA^GFP^ mice^31^, where non-muscle myosin heavy chain II-A is endogenously tagged with GFP, allowing visualization of adherens junction-associated actomyosin, were used to conduct junctional laser cuts. Living intestinal tissue slices were hand-cut using a scalpel and placed into a 35-mm glass-bottom culture dish (Fluorodish) containing 200 µL of Leibovitz medium. A slice anchor (SHD-26GH/10; Harvard Apparatus) was placed on top of the tissue to minimize sample drift. To ensure consistency and maintain tissue viability and tension, imaging and laser ablations were conducted within 45 min following the dissection. The intestinal tissue was imaged using a two-photon laser-scanning microscope (LSM710, Carl Zeiss) in single-photon mode (488 line), at a resolution of 512 × 512 pixels (pixel size = 0.09 µm) with a bidirectional scan lasting 1 s. Cell junctions were ablated using a Chameleon Vision II laser set at 800 nm (three iterations), with laser power of 1–2 mW. The iteration number was optimized to efficiently ablate cell junctions without causing cavitations or additional cell damage. As tissue recoil typically plateaued around 50 s, images were acquired at 1 s intervals (δt = 1 s) for a total of 60 s. Multiple junctions were ablated per mouse for each junction class. Analyses were performed using a custom ImageJ macro as previously described^52^. Briefly, images were denoised using a filtering process to improve the signal-to-noise ratio. Recoil speed was determined by measuring the vertex-vertex distance *d*_vv_ at each time frame, with each value subtracted from *d*_vv_ (*t*_0_) to obtain the amount of strain or recoil (ε(*t*)) at each time point. As junctional strain exhibits a single exponential growth with a defined plateau after ablation, it can be modeled as a Kelvin–Voigt fiber, by fitting to the following equation

1 $$\varepsilon \left(t\right)=\frac{F0}{E}{{{\rm{x}}}}\left(1-{e}^{-\left[\left(\frac{E}{{{{\rm{\mu }}}}}\right){{{\rm{x}}}}t\right]}\right)$$

where F0 is the tensile force at the junction before ablation, *E* is the elasticity of the junction, and µ is the viscosity coefficient. The initial recoil parameter $\frac{F0}{\mu }$ can be extracted after fitting. The fitting and plotting of ε(*t*) and initial recoil values were done in GraphPad Prism. Statistical analyses were performed using individual mice as biological replicates.

### Optical tweezers–based microrheology

#### Optical tweezers setup

A home-built setup was used for optical-trapping measurements^40^. The system combines a fluorescence microscope and an active optical-trapping system. This trapping system has two components: one to precisely control the position of the optical trap on the sample, and a back-focal-plane interferometric (BFPi) system to track the position of the trapped object relative to the center of the laser^53,54^. Furthermore, the use of back focal interferometry in tissue is also unaffected by the light’s scattering due to its position in the Fourier plane of the detector^55^. For calibration and the measurements described below, a steering mirror conjugated to the pupil plane of the microscope (Thorlabs FSM75-P01) is used to achieve nanometric laser deflection. A 3-axis piezoelectric stage (Piezo Concept BIO3) is also used to precisely focus on the trapped object. The optical-trapping system is implemented in a conventional inverted fluorescence microscope (Leica DMI6000 B). The objective lens used is a 100× magnification lens with a numerical aperture of 1.4 (Leica HCX PL APO). To optimize the fill factor of the infrared laser to 85% of the objective lens pupil plane, an X4 afocal telescope consisting of two relay lenses is used (Thorlabs ACA254-050-1064 and ACA254-200-1064). The focused diffraction spot at the objective exit has a width of 600 nm at mid-height, allowing for an efficient optical trap. Imaging and optical tweezers are controlled by combining a National Instruments acquisition card with the Inscoper synchronization box. The Inscoper software manages all synchronization steps.

### Calibration and viscoelastic modulus measurements

Living intestinal tissue slices were hand-cut using a scalpel and placed in an 8-chamber Ibidi glass-bottom slide containing ~50 µL of Leibovitz medium. To ensure consistency and preserve tissue viability and rheological properties, imaging and optical tweezers measurements were performed within 30 min of dissection. For each measurement, an endogenous lipid droplet (refractive index ~1.5; average diameter ~300 nm) was optically trapped and used as a mechanical probe. Optical trapping was performed using a 1064 nm laser, delivering 5 A of power to the intestinal tissue. At this wavelength and power, no detectable effects on tissue behavior were observed. Immediately after trapping, the optical tweezers were calibrated for each measurement using the fluctuation–dissipation theorem (FDT) at a sampling frequency of 2.3 kHz, allowing estimation of the trap stiffness (*k*), detector sensitivity, and calibration parameters. A 360 nm square-wave laser displacement at 2.3 Hz was then applied and tracked using back-focal-plane interferometry to further calibrate the system and probe the viscoelastic response of the cytoplasm. Following calibration, the relaxation force exerted on the trapped droplet was recorded until equilibrium was reached between cytoplasmic resistance and the restoring force of the optical trap.

Here, we briefly summarize our optical tweezers–based approach, recently described in detail by Merle et al.^40^. Using a home-built optical tweezers setup, the stiffness of the optical tweezers is estimated with an absolute error of 3% using the following expression derived from the fluctuation–dissipation theorem [9], which takes into account all harmonics of the square excitation:

2 $${k}_{t}\left(n{{{{\rm{\omega }}}}}_{d}\right)={\sum }_{n=1,3,5\ldots }^{\infty }-2{k}_{b}{TRe}\left[\frac{{P}_{{active}}}{{P}_{{passive}}}\right]$$

where ${P}_{{active}}$ is the active power spectrum and ${P}_{{passive}}$ is the passive power spectrum. The high-frequency analysis and the number of harmonics provide a highly robust estimation of ${k}_{t}$. The mechanical response of the medium can then be reconstructed independently at each harmonic frequency once ${k}_{t}$ is known.

The complex susceptibility of the trapped droplet is obtained from the passive fluctuations as follows, for each harmonic frequency ${{{\rm{\omega }}}}={{{\rm{n}}}}{{{{\rm{\omega }}}}}_{{{{\rm{d}}}}}$

3 $${{{\rm{\chi }}}}\left({{{\rm{\omega }}}}\right)=\frac{\left\langle {\left|{{{\rm{x}}}}\left({{{\rm{\omega }}}}\right)\right|}^{2}\right\rangle }{2{{{{\rm{k}}}}}_{{{{\rm{B}}}}}{{{\rm{T}}}}}$$

The inverse susceptibility can then be written as:

4 $${\chi }^{-1}\left({{{\rm{\omega }}}}\right)={{{{\rm{k}}}}}_{{{{\rm{t}}}}}+6{{{\rm{\pi }}}}{{{\rm{R}}}}{{{{\rm{G}}}}}^{*}\left({{{\rm{\omega }}}}\right)$$

where

5 $${{{{\rm{G}}}}}^{*}\left({{{\rm{\omega }}}}\right)={{{{\rm{G}}}}}^{{\prime} }\left({{{\rm{\omega }}}}\right)+{{{\rm{i}}}}{{{{\rm{G}}}}}^{{\prime} {\prime} }\left({{{\rm{\omega }}}}\right)$$

is the effective complex viscoelastic modulus of the surrounding medium probed at frequency ${{{\rm{\omega }}}}$, and *R* is the radius of the trapped droplet.

Therefore, the complex modulus is directly reconstructed for each odd harmonic of the square-wave drive.

6 $${G}^{*}\left(n{{{{\rm{\omega }}}}}_{d}\right)=\frac{1}{6{{{\rm{\pi }}}}R}\left[{{{{\rm{\chi }}}}}^{-1}\left(n{{{{\rm{\omega }}}}}_{d}\right)-{k}_{t}\right]$$

This multi-harmonic reconstruction method provides independent estimates of G′(ω) and G″(ω) over a broad frequency range. Furthermore, the dispersion of the reconstructed values across harmonics provides a direct estimate of experimental uncertainty and mechanical heterogeneity. A phenomenological viscoelastic model (e.g., power-law or Kelvin–Voigt behavior) can subsequently be fitted to *G*′(ω) and *G*″(ω) when needed.

The following equation, derived from 9, was used to fit a mixture of cytosol and actin medium, and has proven to be highly effective:

7 $${G}^{*}\left(\omega \right)=\frac{1}{6\pi R}\left[{k}_{m0}+\frac{{k}_{m1}{\left(i\omega \right)}^{\alpha }}{\Gamma \left(\alpha \right)}+i\omega \gamma \right]$$

### Intestinal permeability assay

Mice were orally administered 4 kDa FITC-Dextran (Sigma, 46944) at 8 mg per mouse in a randomized order. Two hours later, mice were anesthetized using isoflurane, and blood was collected by cardiac puncture and allowed to coagulate for 30 min at RT. Blood samples were centrifuged (2200 × *g*, 15 min), and serum was collected. Serum from non-gavaged mice was used as a blank. FITC-Dextran concentration in serum was measured in duplicates using a Fluoroskan FL with SkanIt software plate reader.

### Cytokine quantification by ELISA

Cytokine levels (TNF-α, IL-6, and IFN-γ) were measured using the ELLA automated ELISA system (Biotechne). Control, succinate, or DSS-treated mice (used as a positive control) were anesthetized using isoflurane, and blood was collected by cardiac puncture. Blood samples were centrifuged (1000 × *g*, 20 min) and serum was collected and diluted according to the manufacturer’s protocol.

### Organoids

#### Intestinal crypt isolation and 3D organoid culture

Wild-type mice (2 months old) were sacrificed, and the jejunum was isolated, cut longitudinally, washed in PBS, and minced with a scalpel. Tissue was dissociated in PBS-EDTA 2 mM solution for 30 min at 4 °C. After shaking, the fraction containing crypts was filtered through a 70 µm strainer (Corning). The solution was centrifuged at 200 × *g* at 4 °C for 4 min to separate crypts from single cells. The supernatant was removed, and the pellet was resuspended in 10 mL of resuspension solution (DMEM/F12 + 2% AA) and filtered through a 70 µm strainer to remove the villi. The solution was then centrifuged at 200 × *g* at 4 °C for 6 min. The pellet was resuspended in 1/1 Matrigel (Corning, 356231)/ENR medium and plated as 40 µL drops in 24-well plates. After incubation for 15 min at 37 °C and 5% CO_2_ in a humidified atmosphere, the wells were filled with 350 µL of ENR medium. Organoids were passaged every 3–4 days by mechanical dissociation.

##### ENR medium

Advanced DMEM F12 (Gibco, 12634010), supplemented with 2% AA (Gibco, 11570486), 2.5% Glutamax (Gibco, 13462629), 100 ng/mL Noggin (StemCell Technologies), 20 ng/mL EGF (Peprotech, 315-09), 500 ng/mL R-Spondin 1 (Peprotech, 315-32), 10 ng/mL mbFGF (Peprotech, 450-33), 1× N2 (Gibco, 11520536), and 1× B27 (Gibco, 11500446).

#### Generation of 2D organoids

Prior to 2D culture, 3D organoids were pre-treated to promote proliferation and multipotency by culturing them in ENR-CV medium – ENR medium supplemented with 3 µM CHIR 99021 (Peprotech, 2520691) and 1 µM valproic acid (Sigma, P4543) for at least 3 days.

For 2D seeding, glass coverslips (13 mm diameter) were coated with extracellular matrix proteins. A drop of rat tail Collagen I (250 µg/mL Sigma; C3867) and Laminin 1 (100 µg/mL; Sigma, L2020) diluted in PBS was applied to each coverslip and incubated for 1 h at 37 °C. Excess matrix was removed, and coverslips were washed twice with PBS prior to use.

3D organoids were mechanically dissociated, typically using 4–5 wells per coverslip. Cells were centrifuged at 200 × *g* for 3 min at 4 °C, and the pellet was resuspended in ENR-CV medium. A 50 µL drop of the cell suspension was added onto each coated coverslip. After 1 h incubation at 37 °C in a humidified incubator with 5% CO_2_, 350 µL of ENR-CV medium supplemented with 10 µM ROCK inhibitor Y27632 (Santa Cruz) was added. Medium was changed daily. 2D organoids were maintained in ENR-CV + ROCKi for one day, then switched to differentiation medium containing 10 µM ROCKi. All experiments were performed 3–5 days after seeding.

To promote goblet cell differentiation, 3 µM DAPT (Tebu-bio, 28T6202) and IWP-2 (Tebu-bio, 28T2702) were added to fresh medium^25^.

For blebbistatin treatment, 15 µM blebbistatin or vehicle (DMSO) was added to the 2D monolayers for 3 h. Samples were then fixed and processed for analysis.

#### Immunofluorescence in 2D organoids

The organoid monolayers were fixed in 4% PFA for 10 min and washed 3 times with PBS. Samples were permeabilized with 0.1% Triton X-100 (Sigma) for 10 min, then washed 3 times with PBS. After 1 h in the blocking solution (10% FBS/PBS), the primary antibody was added (diluted in 10% FBS/PBS) and incubated O/N at 4 °C. After three washes of 5 min each in PBS, secondary antibody was added with DAPI and with or without WGA and phalloidin, in 10% FBS/PBS, and incubated 1 h at RT. Samples were washed three times for 5 min each and mounted on slides using mounting agent (Aqua-Poly/Mount, Polysciences). To differentiate goblet and Paneth cells, lysozyme staining was used, and goblet cells were identified as lysozyme^-^ and WGA^+^ cells.

### Immunoblotting

For each sample, the small intestines were dissected and opened longitudinally. Tissue was incubated in solution containing 30 mM EDTA and 1 mM DTT in PBS for 20 min on ice, followed by 30 mM EDTA/PBS for 15 min at 37 °C. The epithelium was detached from the mucosa by vigorous shaking, and the epithelial fraction was collected by centrifugation (200 × *g* for 5 min) and lysed in RIPA buffer. Protein concentration was quantified using the DC protein assay (Bio-Rad) and normalized, then 4× Laemmli buffer was added. 25 µg of total protein was used for electrophoresis on 4–20% sodium dodecyl sulfate (SDS) polyacrylamide gels under reducing conditions (Ready Gel, Bio-Rad), and transferred to a nitrocellulose membrane (Bio-Rad) following standard procedures. The membranes were blocked with blocking solution (Everyblot, Bio-Rad) for 30 min. The membranes were incubated with primary antibodies O/N at 4 °C, followed by incubation with Horseradish peroxidase-conjugated secondary antibodies for 1 h at RT. The immunoreactive bands were visualized using chemiluminescence detection reagent (ECL Clarity, Bio-Rad) and ChemiDoc imaging system (Bio-Rad).

### Confocal 3D imaging

To ensure consistency across samples, all image stacks were acquired at the mid-region of the villi. Imaging was performed using an inverted, laser-scanning confocal microscope LSM880 (Zeiss) equipped with 405 nm, Argon, 561 nm, and 633 nm lasers. A 63× oil immersion objective was used to acquire images at a resolution of 1024 × 1024 pixels. Z-stacks were collected with a step size of 0.3 µm.

### High-resolution (Airyscan) 3D tissue imaging

Images were acquired on a laser-scanning confocal microscope LSM880 (Zeiss) equipped with an Airyscan module, using a 63× oil objective, at a resolution of 1024 × 1024 pixels. Z-stacks were collected with a step size of 0.2 µm. Airyscan Z-stacks were processed in Zen software (Zeiss) using the Airyscan processing module.

### RIM

The 3D images were acquired using an inverted microscope (TEi Nikon) equipped with a ×100 magnification, 1.49 NA objective (CFI SR APO 100XH ON 1.49 NIKON). A SCMOS camera (ORCA-Fusion, Hamamatsu) was used. Commercial acquisition software (INSCOPER SA) was used for 3D-RIM acquisition. A collimated beam with a diameter of 2.2 mm was generated using fast diode lasers (Oxxius) with wavelengths centered at 488 nm (LBX-488-200-CSB). The polarized beam was rotated at an angle of 5° before hitting an X10 beam expander (GBE04-A) and producing an 18 mm TEM00 beam through a spatial light modulator. A fast spatial light phase binary modulator (QXGA fourth dimension) was conjugated to the image plane to generate 200 random illuminations through each plane, as detailed in ref. ^56^.

Image reconstruction was performed using the RIM reconstruction workflow available on GitHub (https://github.com/teamRIM/tutoRIM) and described in ref. ^56^. Briefly, raw image stacks acquired under random illumination were processed to generate reconstructed 3D images with improved contrast and resolution. The following reconstruction parameters were used: prefiltering Wiener parameter, 0.08; inversion parameter, 0.08; equalization parameter, 0.1.

### Image analysis

All images were processed for brightness and contrast adjustments and analyzed using Fiji software. Apical cell area was measured by segmentation of ZO-1 staining using the Tissue Analyzer plugin. Fracture area and junctional intensity were manually measured using the Fiji Measurement Tool. Line profile intensity was also manually measured by tracing a line of consistent length and using the Plot Profile module. Average junctional E-cadherin signal intensity was plotted along the rescaled junctional length in R. The cell shape index was calculated as (perimeter/√area) and measured manually based on the ZO-1 signal. Intracellular mucin granule volume measurement was performed using Imaris software, with 3D segmentation based on the WGA signal using the Cell function.

### Statistics

In all figures, n represents the number of biological replicates (individual mice for in vivo or ex vivo experiments or independent organoid cultures for in vitro experiments). For each condition, at least three independent technical replicates were performed. Statistical analyses were performed using GraphPad Prism 8.0.1. For each experiment, normality was assessed using the Shapiro-Wilk test. For normally distributed values, differences between two groups were analyzed using an unpaired *t*-test, while non-normally distributed values were analyzed using the Mann–Whitney test. For comparisons across three or more groups, multiple *t*-tests with FDR correction were used. Data are presented as mean ± SD. Symbols used are: ns: *p* > 0.05; *p ≤ 0.05; **p ≤ 0.01; ***p ≤ 0.001; ****p ≤ 0.0001.

### Reporting summary

Further information on research design is available in the Nature Portfolio Reporting Summary linked to this article.

## Supplementary information

Supplementary information

Description Of Additional Supplementary File

Supplementary Movie 1

Supplementary Movie 2

Supplementary Movie 3

Supplementary Movie 4

Supplementary Movie 5

Supplementary Movie 6

Reporting Summary

Transparent Peer Review file

## Source data

Source data

## Data Availability

Data supporting the findings of this study are available within the paper and its Supplementary Information. Microscopy data reported in this paper will be shared by the lead contact on request, due to large file sizes. Source data are provided with this paper.

## Code availability

Custom codes are deposited on Code Ocean (10.24433/CO.7420253.v1).
