# Supplementary material for: Goblet cells mechanically breach the epithelial barrier in gut homeostasis
Source: Nat Commun. 2026 Jul 25;17:9078. doi: 10.1038/s41467-026-76034-0 (PMC13518819; doi:10.1038/s41467-026-76034-0)
Supplement: Supplementary file 1 [file 41467_2026_76034_MOESM10_ESM.pdf]

nature portfolio

Peer Review File

Goblet cells mechanically breach the epithelial barrier in gut homeostasis

Corresponding Author: Dr Denis Krndija

This manuscript has been previously reviewed at another journal. This document only contains information relating to versions considered at Nature Communications.

This file contains all reviewer reports in order by version, followed by all author rebuttals in order by version.

Version 0:

Reviewer comments:

Reviewer #1

(Remarks to the Author)
Epithelial tissues are composed of diverse cell types that differ in function, shape, and mechanical properties. As epithelia primarily serve as physical barriers, they must remain tightly sealed under homeostatic conditions. This raises important questions about how such tissues accommodate structural diversity and mechanical variability while preserving their integrity. Further, in recent years, the field has begun to recognise that living tissues frequently undergo controlled fracture as part of normal development and homeostasis. Rather than representing failures, these breaks are carefully regulated to allow tissues to change shape or adapt to mechanical deformation.

In this study, Creff et al. use a powerful combination of 3D tissue imaging, mouse models, explants, and organoids to explore how the gut epithelium tolerates the presence of bulky, mucus-secreting goblet cells. The authors show that goblet cells mechanically induce transient tight junction fractures between neighbouring enterocytes. Importantly, these fractures are not static defects; they are dynamically regulated through the reinforcement of desmosomes, adherens junctions, and myosin II–dependent contractility. Their findings enhance our understanding of how epithelial tissues maintain barrier integrity while accommodating mechanical stress and cellular diversity. The imaging is remarkable, the manuscript is clearly written, and the data are both rigorously quantified and well presented. This timely study contributes to a growing appreciation within the developmental mechanics field that tissue fractures are an inherent and functional aspect of morphogenesis. Congratulations to the authors!

Major Comments:

1. The accessible E-cad staining approach is a valid proof-of-concept of the accessibility of the AJ components in when tight junctions are breached. However, in extended Fig 3A, Fig 3B, we note that E-cad is already significantly enriched in E-E 1 junctions neighbouring the GC. While we understand that this accessible staining has been performed in other publications as well, how do the authors reliably distinguish between increased staining due to accessibility and increased staining due to increased levels of E-cad?

2. Further, given that there is 20% of fractures at adherens junctions, do the authors also see similar “accessible” patterns of any other junctional components like desmoglein?

3. An important and intriguing conclusion of the paper is the role of enterocyte rheology in the fracturing process. The authors have inferred tissue rheology through modelling and cell shape index. It would be valuable to directly validate these conclusions by measuring tissue rheology using techniques such as AFM or micropipette aspiration. The 2D organoid system used (Extended Data Fig. 12) might be particularly amenable to such measurements?

4. Many figures e.g Extended Figure 6C, Fig 2D, Fig 2H, show a population of cells that stain very strongly for F actin. Are these tuft cells? These cells are noticeably smaller but similarly do not have “wiggly” boundaries with neighbouring enterocytes. Do these cell types also induce fractures? Can the authors speculate on why this might or might not be the case

and provide more details on the role of these cells in maintaining the gut epithelium integrity?

5. The laser ablation experiments need more explanations. In Extended Fig 8, laser cuts of G-E and surrounding E-E junctions show that E-E junctions recoil 4 times faster than G-E junctions. We were under the impression that G-E junctions would be under more tension due to the increased pressure from the GC cells. In contrast, the E-E junctions are “wiggly”, which we thought would indicate that they are under less tension. Is this difference purely due to active myosin (as seen in increased P-MLC staining)? If myosin activity is inhibited, would the authors then expect the G-E junction to recoil faster due to the pressure exerted by GCs?

6. The authors argue that the stereotypical outward curvature of the G-E junction arises from GCs having higher pressure than enterocytes, leading them to hypothesise that GCs exert mechanical forces on their neighbours. From a physical perspective, this is intuitive and makes sense. However, when the authors treat mice with succinate to induce GC hypertrophy and increase pressure, the shape of the GC appears to change (Fig 2D), and G-E junctions no longer have the same distinct outward curvature, which might indicate that they may have lower pressure. This part is not very clear and a better explanation is needed. Can the authors give a reason why this might be the case? Is there some natural variation in this outward curvature, and does this correlate with GC size and fractures?

7. From our understanding, Villin:Cre will also drive knockout of NMIIA in Goblet cells, which may be more affected by this, given the higher levels of NMIIA-GFP in goblet cells. From Fig 4E, it appears that GCs also look slightly bigger in the KO (although we acknowledge it is non-significant in Extended Data Fig 9E). Can the authors disentangle the effect of a NMIIA KO in GCs from enterocytes? Would they expect a loss in GAFs as well in a GC-specific knockout e.g. Muc2-Cre line?

8. The paper generally references fractures at the level of TJs, AJs, and Desmosomes. Can the authors provide a high-resolution Apical-Basal or Basolateral cross-section image to show how these fractures appear at these sites? Further explanation on how the authors distinguish between fractures at the level of TJs and AJs would also be helpful.

9. The tissue-scale fractures that form in response to shear stress challenge (Fig. 2H; Extended Data Fig. 6C) are striking and are clear hallmarks of mechanical failures. Can the authors provide (if possible) some videos or images of the fracturing process?

Further, are NMIIA KO mice less susceptible to tissue-scale fracturing in response to shear, as GAFs are smaller?

Minor Comments:

1. Fig. 2A zoomed region is slightly different from the box

2. “unrecognized mechanical impact of goblet cells in destabilizing epithelial cohesion and compromising barrier function, highlighting the importance of cellular heterogeneity.” Line 33

Slightly Clunky writing, makes it sound like compromised barrier function is desirable

3. Typo: Extended Data Figure 1D labelled as 1C. As above, it would also be helpful to see some images of fractures at AJs and Desmosomes.

4. The Large intestine has a denser proportion of goblet cells. What do we expect to see in terms of TJ fractures? Can the authors speculate on how the large intestine deals with this mechanical challenge?

5. It would be helpful for the general audience if the terms in Extended Fig 3. D-F are briefly explained/defined in the legends (e.g. curvilinear coordinates s and θ, as defined in the supplementary methods)

6. The authors have rigorously quantified their data, which is commendable. While we appreciate potential space constraints, in some cases such as Extended Fig 1D, Extended Fig 3I, Fig 2F, it will be helpful for the reader to see the qualitative data as well.

7. For Fig 4L-N, statistical comparisons between NMIIAKO and NMIIAKO succinate should also be performed, and p values should be given; this should similarly be performed for Extended Fig 11.

8. Typo in line 616 (should be perimeter/area instead of perimeter/)

9. Typo in Extended Figure 9A legends (Right and Left swapped)

(Remarks on code availability)

Reviewer #2

(Remarks to the Author)
I co-reviewed this manuscript with one of the reviewers who provided the listed reports. This is part of the Nature Communications initiative to facilitate training in peer review and to provide appropriate recognition for Early Career Researchers who co-review manuscripts.

(Remarks on code availability)
I co-reviewed this manuscript with one of the reviewers who provided the listed reports. This is part of the Nature Communications initiative to facilitate training in peer review and to provide appropriate recognition for Early Career Researchers who co-review manuscripts.

Reviewer #3

(Remarks to the Author)
I have reviewed the paper by Creff et al. The paper studies the impact of goblet cells in the stability of the intestinal barrier. They combined in vivo and ex vivo experimental data together with modeling approaches to understand the mechanical role of goblet cells in the intestine. They found that the mechanical pressure induced by goblet cells leads to the local fracture of tight junctions at the interface with the neighboring enterocytes. I found the paper of interest for the readers of Nature Communications. However, there are a few points that the authors need to address before publication.

1. An easy one. The paper includes only four main figures and 12 extended (supplementary) figures. To my knowledge, Nature Communications does not impose strict limits on the number of main figures. I therefore recommend that the authors reorganize the figures to incorporate some of the key data currently placed in the supplementary section into the main figures. This will reduce the need for readers to constantly switch between the main text and the supplementary material. Important elements, such as data on the model, laser ablation, and others, should be highlighted more prominently in the main text.
2. The maintenance of epithelial integrity is crucial. So, under physiological conditions, the functional role of the fractures induced by goblet cells remains unclear. I believe the authors should further discuss this aspect to clarify its biological significance.
3. In Figure 2D, the rupture or discontinuities in ZO-1 staining (as described in Figure 1) are not clearly visible. The ZO-1 signal appears continuous, including at the tricellular junctions, under both control and succinate-treated conditions. The authors should clarify this apparent discrepancy and explain how it aligns with their earlier observations.
4. Figure 2F (dextran incorporation) appears to be the only experiment directly assessing barrier function. However, it is not well described in the text. The authors should provide a clearer explanation of the experimental setup and include both quantitative measurements and representative images to support their conclusions.
5. The authors use a peristaltic pump to increase the pressure inside the tissue and report an increase in shear stress of 1 Pa. However, there may also be a contribution from normal stress (i.e., luminal pressure). It appears that the projected area of the cells increases following flow application. Could this change be driven by normal stress rather than shear stress? Would it be possible for the authors to estimate the magnitude of the normal stress in their system?
6. In the laser ablation experiments comparing tension at G–E and E–E junctions, it would be valuable to also include E–E junctions that are in contact with goblet cells. It is currently unclear which E–E junctions are being probed. Are they located near goblet cells or not? One would expect G–E junctions to be under tension, but the E–E junctions shown to fracture exhibit a wavy morphology (see ZO1 staining fig 1), which may suggest compression rather than tension. Additionally, the example shown in Extended Fig. 8 for a G–E junction does not appear to feature a clearly bulging goblet cell, which could influence the measurements. These points require clarification.
7. The authors frequently refer to tissue fluidity (e.g., line 238), but it remains unclear whether actual cell–cell rearrangements were observed. This point needs to be clarified and, ideally, directly measured. Under the current culture conditions, I do not believe that the shape index alone is sufficient to infer fluid-like behavior in the tissue.
8. In the model, it is unclear how the distinct properties of enterocytes and goblet cells are incorporated. Are both cell types assumed to have the same mechanical properties? How is their spatial distribution determined or imposed? It is also not evident how these differences are reflected in the evolution equations, for example, in equation (S7). Further clarification on how cell-type-specific features are modeled would be helpful.

(Remarks on code availability)

Reviewer #4

(Remarks to the Author)
The study presented by Creff et al provides novel and relevant information about how the heterogeneous cell population

within the intestinal epithelium contributes to maintenance or breaching of the intestinal barrier function. In fact, the demonstrated that the presence of goblet cells, with their specific cell shape, alters the physical properties and cohesion within the epithelial layer.

I believe these findings are sound and important for the scientific community, including biophysics aspects, in order to better understand epithelial barrier fucntion. The methodology, the data, figures and the draft meet the quality requirements for publication. In addition, drawn conclusions are supported by the collected data.

Nevertheless, there are some aspects which are not entirely clear to me, and I think might require revision from the authors. The authors took advantage of succinate to induce goble cell hyperplasia/hypertrophia, but succinate mediates other effects in the intestinal epithelium, such as the activation of tuft cells and the secretion of IL-25. It would be important to demosntrated that the succiante-mediated effect uniquely depend on the impact on goblet cells, while the effect on other IECs is not relevant. It is also intriguing the fact that the auhtors used two different strategies to promote the goblet cell hyperplasia in vivo (succinate) and in vitro (DAPT; IWPT2). How comparable are these two situations? actually, succiante did not increase the number of goblet cells, but only the amount of mucus production, while the in vitro conditions affected both number and function of goblet cells.

Another important aspect is the description of biological/technical replicates. In most figure legends, it is mentioned n = x number of experiments, but there is no explanation about the biological replicate number within each experiment. In contrast, in the methods section, it is mentioned that the n describes the number of mice (in vivo) or the number if experiments (in vitro). I think the information is not entirely clear, and it is therefore hard to judge the signifcance of the presented data, in some cases.

Minor comments:
- it would be helpful revising the order of the figures, and adapt them to their appearance in the text. Sometimes, the reader has to go back and forth through the figures, because they do not appear in order.
- ED Fig. 1: it contains twice the subfigure C.
- ED Fig. 8a: pictures not really representative of the quantification in ED Fig. 8B
- ED Fig. 1B: why the mucus is going beyond the GC?

(Remarks on code availability)

Version 1:

Reviewer comments:

Reviewer #1

(Remarks to the Author)
I am very satisfied with the revised version—kudos to the authors. It’s a rigorous and beautiful study, and many congratulations!

(Remarks on code availability)

Reviewer #2

(Remarks to the Author)
I co-reviewed this manuscript with one of the reviewers who provided the listed reports. This is part of the Nature Communications initiative to facilitate training in peer review and to provide appropriate recognition for Early Career Researchers who co-review manuscripts.

(Remarks on code availability)

Reviewer #3

(Remarks to the Author)
I would like to thank the authors for the revised version of the manuscript. They have clearly addressed my questions, and I can recommend publication of the article.

have only one final comment, but I do not need to re-review the paper. It should be made clear that the rheological measurements with optical tweezers were performed intracellularly. Indeed, probing cell rheology from the outside or from the inside can lead to different, and sometimes unexpected, results. For example, disruption of the actin cytoskeleton does not necessarily appear to affect intracellular mechanical properties, as reported by Muenker et al. in eLife 2024.

I would therefore be slightly more cautious with conclusions stating that the cells have lower compressive resistance, since

this interpretation is more directly related to cortical activity than to intracellular mechanical properties. However, it would make sense that NMIIA-KO cells may also exhibit reduced cortical stiffness. This distinction may also explain why the authors obtain relatively low values for the storage modulus.

(Remarks on code availability)

Reviewer #4

(Remarks to the Author)
the authors addressed or clarified all my comments. I have no furhter comments.

(Remarks on code availability)

Open Access This Peer Review File is licensed under a Creative Commons Attribution 4.0 International License, which permits use, sharing, adaptation, distribution and reproduction in any medium or format, as long as you give appropriate credit to the original author(s) and the source, provide a link to the Creative Commons license, and indicate if changes were made.
In cases where reviewers are anonymous, credit should be given to 'Anonymous Referee' and the source.
The images or other third party material in this Peer Review File are included in the article's Creative Commons license, unless indicated otherwise in a credit line to the material. If material is not included in the article's Creative Commons license and your intended use is not permitted by statutory regulation or exceeds the permitted use, you will need to obtain permission directly from the copyright holder.
To view a copy of this license, visit https://creativecommons.org/licenses/by/4.0/

Response to Reviewers

We thank the Reviewers for their constructive comments and valuable suggestions. Below, we provide a detailed, point-by-point response to each comment.

Reviewers' comments are reproduced below in blue, and our responses are provided in black.

Reviewer #1 (Remarks to the Author):

Epithelial tissues are composed of diverse cell types that differ in function, shape, and mechanical properties. As epithelia primarily serve as physical barriers, they must remain tightly sealed under homeostatic conditions. This raises important questions about how such tissues accommodate structural diversity and mechanical variability while preserving their integrity. Further, in recent years, the field has begun to recognize that living tissues frequently undergo controlled fracture as part of normal development and homeostasis. Rather than representing failures, these breaks are carefully regulated to allow tissues to change shape or adapt to mechanical deformation.

In this study, Creff et al. use a powerful combination of 3D tissue imaging, mouse models, explants, and organoids to explore how the gut epithelium tolerates the presence of bulky, mucus-secreting goblet cells. The authors show that goblet cells mechanically induce transient tight junction fractures between neighbouring enterocytes. Importantly, these fractures are not static defects; they are dynamically regulated through the reinforcement of desmosomes, adherens junctions, and myosin II–dependent contractility. Their findings enhance our understanding of how epithelial tissues maintain barrier integrity while accommodating mechanical stress and cellular diversity. The imaging is remarkable, the manuscript is clearly written, and the data are both rigorously quantified and well presented. This timely study contributes to a growing appreciation within the developmental mechanics field that tissue fractures are an inherent and functional aspect of morphogenesis. Congratulations to the authors!

Major Comments:

1. The accessible E-cad staining approach is a valid proof-of-concept of the accessibility of the AJ components in when tight junctions are breached. However, in extended Fig 3A, Fig 3B, we note that E-cad is already significantly enriched in E-E 1 junctions neighbouring the GC. While we understand that this accessible staining has been performed in other publications as well, how do the authors reliably distinguish between increased staining due to accessibility and increased staining due to increased levels of E-cad?

We thank the reviewer for this important comment. Indeed, as shown in Fig. 3A, B, E-cadherin is significantly enriched in E-E 1 junctions. However, it is critical to note that in the accessible E-cadherin assay (Fig. 1I, J), the luminally accessible E-cadherin staining is performed **prior to**

tissue permeabilization. Therefore, the detected signal reports only the fraction of E-cadherin exposed to the luminal side, not the total cellular levels.

The key observation supporting our interpretation is that accessible E-cadherin signal is largely restricted to E-E1 junctions, adjacent to the GCs, with a markedly weaker signal at more distant, E-E2 junctions (quantified in Fig. 1N).

This approach has been previously used in the context of *Listeria monocytogenes* infection [1], where GCs were also shown to be associated with luminally accessible E-cadherin. In that study, the authors speculated that mucus secretion might transiently remodel junctions, but they did not identify the underlying structural fractures. Our study not only confirms the presence of accessible E-cadherin at GC-adjacent junctions, but also provides a plausible mechanistic explanation, linking this accessibility to the occurrence of the GAFs.

To further validate this approach in our system, we performed accessible E-cadherin staining on tissue treated with EGTA, which chelates extracellular Ca^{2+} and is widely used to acutely disrupt Ca^{2+}-dependent adhesion and induce junction opening, providing a well-established positive control for junction disruption in epithelial tissues **(Fig. R1)**. In control samples, accessible E-cadherin is detected only at E-E 1 junctions. After 5 min of EGTA treatment, the signal increases at E-E 2 junctions, and after 10 min, it extends further across the tissue. Importantly, at 10 min, the accessible E-cadherin junctional intensity no longer correlates with proximity to GCs, as no difference is observed between E-E 1 and E-E 2 junctions **(Fig. R1B, C)**. At the same time, no change in **total E-cadherin** junctional intensity is observed across different EGTA time points **(Fig. R1D, E)**, demonstrating that the accessible E-cadherin signal at E-E 1 reflects luminal exposure due to tight junction fractures, rather than differences in total E-cadherin levels. Under homeostatic conditions, where accessibility is confined to GC-adjacent junctions, the accessible E-cadherin assay therefore serves as a reliable readout of tight-junction breaches.

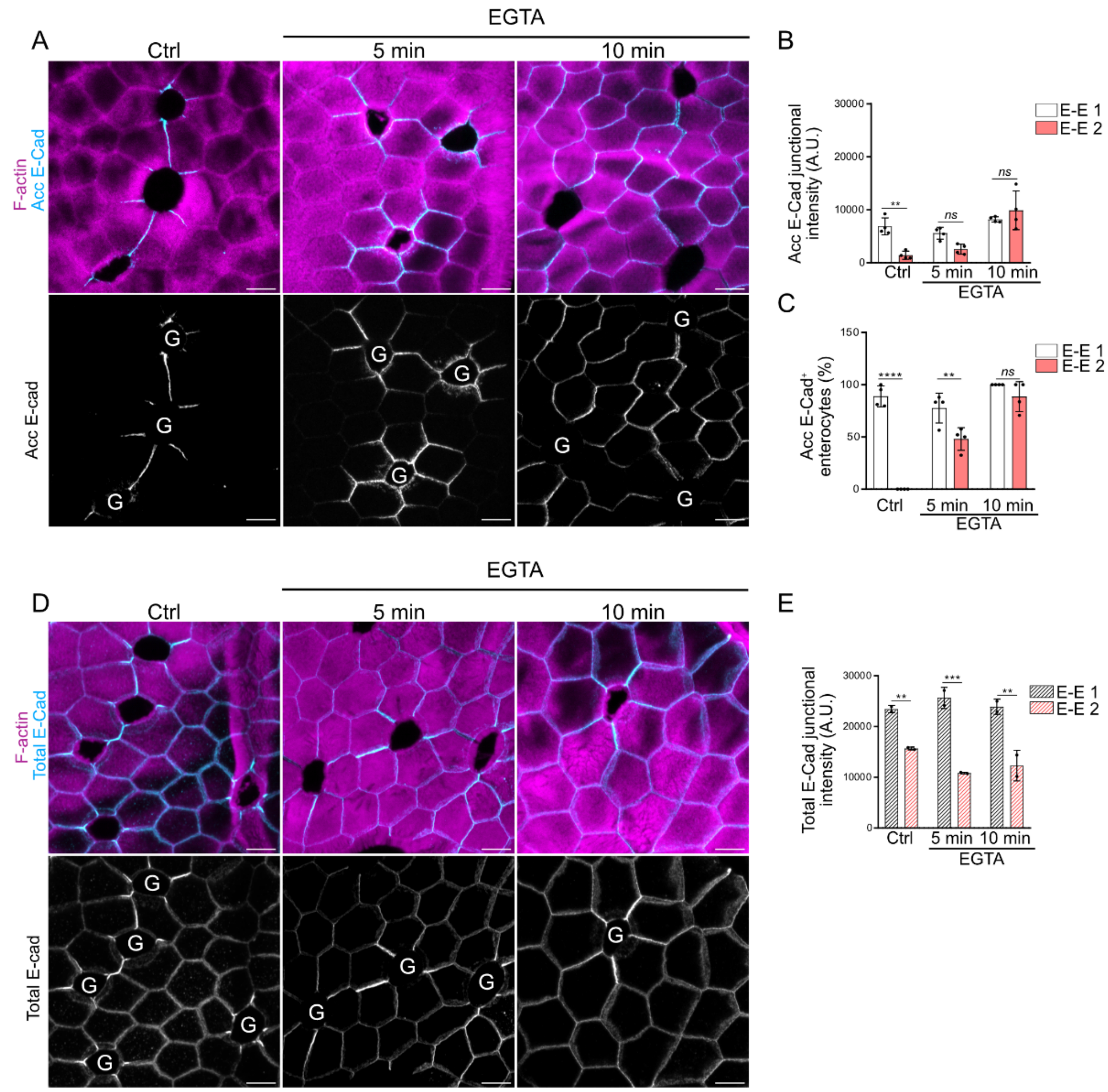

Fig. R1. Validation of accessible E-cadherin staining assay. (A) representative *en face* images of tissue stained for accessible E-cadherin in control and after 5 and 10 min of EGTA treatment. (B) Bar chart displaying average accessible E-cadherin junctional intensity. (C) Bar chart displaying average percentage of accessible E-cadherin positive cells. N=4. (D) representative *en face* images of tissue stained for total E-cadherin in control and after EGTA treatment. (E) Bar chart displaying average total E-cadherin junctional intensity. Multiple t-test, **: $p < 0.01$, ***: $p<0.001$. N=2. Scale bars, 5µm.

2. Further, given that there is 20% of fractures at adherens junctions, do the authors also see similar "accessible" patterns of any other junctional components like desmoglein?

We thank the reviewer for this interesting comment. We had not previously explored this aspect in detail. Our reasoning was based on the structural difference between tight junctions (TJs) and adherens junctions (AJs).

Unlike TJs, which form a tight seal and maintain the epithelial barrier, AJs do not completely restrict paracellular diffusion. Therefore, even when AJs are intact, they may allow penetration of large molecules such as antibodies, potentially allowing staining of desmosomal components.

Consistent with this, ultrastructural studies using electron microscopy show that TJs appear as membrane fusions or "kissing points," reflecting close contact between adjacent membranes. In contrast, at the level of AJs, the opposing plasma membranes are typically 15–20 nm apart [2], a distance sufficient to permit diffusion of antibodies (~150 kDa, ~10 nm) [3].

3. An important and intriguing conclusion of the paper is the role of enterocyte rheology in the fracturing process. The authors have inferred tissue rheology through modelling and cell shape index. It would be valuable to directly validate these conclusions by measuring tissue rheology using techniques such as AFM or micropipette aspiration. The 2D organoid system used (Extended Data Fig. 12) might be particularly amenable to such measurements?

We thank the reviewer for this valuable comment. We fully agree that directly assessing enterocyte rheology would provide an important and complementary validation of our conclusions.

In this study, we inferred tissue fluidity using the cell shape index, following a well-established and experimentally validated framework, which demonstrates that a critical threshold in the shape index corresponds to a transition between fluid-like and solid-like tissue states – commonly referred to as the "jamming–unjamming" transition [4–8]. While this approach provides robust insight into collective epithelial mechanics, we recognize the importance of direct mechanical measurements.

To this end, we attempted to perform AFM on 2D intestinal organoids. Unfortunately, this approach proved technically challenging in our system due to the presence of mucus layer secreted by GCs, covering the epithelium. As shown in **Fig. R2**, the mucus layer (visible as dark regions in AFM topography images) interfered with the ability to reliably probe the surface. Furthermore, the apical surface of enterocytes, densely packed with microvilli, introduces substantial artefacts and biases in AFM-based mechanical measurements, and would similarly prevent micropipette aspiration, which requires a smooth and accessible plasma membrane for accurate deformation analysis.

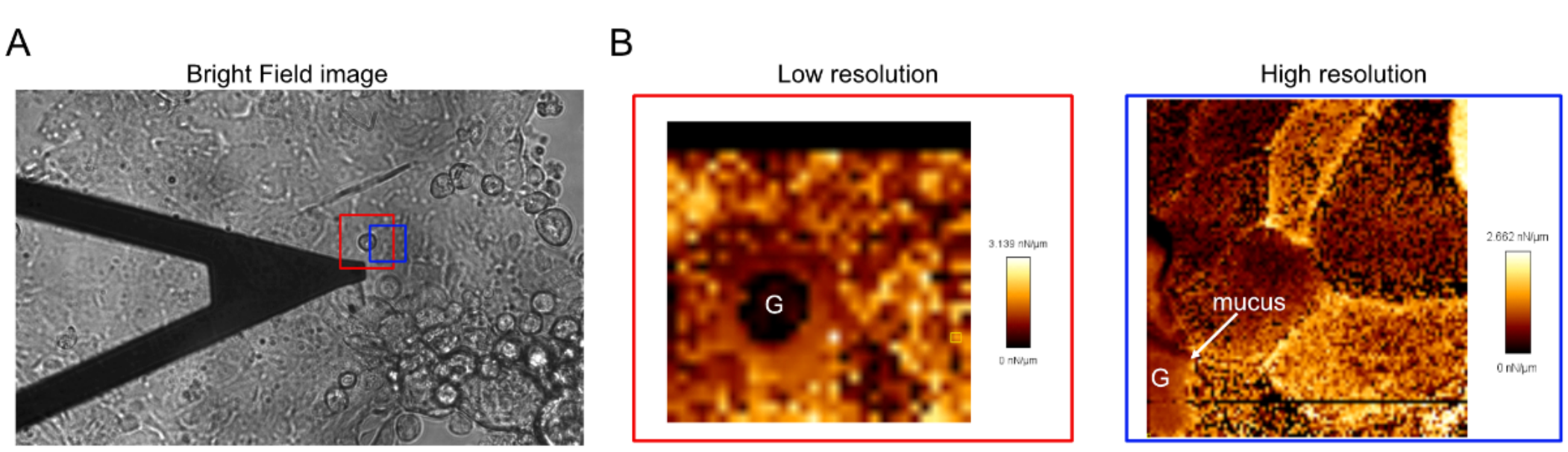

Fig. R2. AFM measurement on 2D intestinal organoids (A) Brightfield image of 2D intestinal organoids probe with AFM cantilever. (B) AFM imaging at low (left) and high (right) resolution showing that mucus is preventing AFM measurement.

To overcome these technical issues, we developed and implemented a new optical tweezers-based approach applied to gut explants, enabling localised intracellular force application and mechanical readout within native tissue geometry.

We use a home-built setup combining fluorescence microscopy with an active optical system. This system allows trapping of an intracellular lipid droplet in living tissue and using it as a probe to measure cytoplasmic viscoelastic properties. An interferometric detection method, insensitive to optical scattering and aberrations, is used to track square-wave laser displacements applied to the trapped droplet[9]. This enables calculation of the trap stiffness k (pN/µm), the conversion factors of the interferometric tracking system β (V/nm), and the viscoelastic response of the medium, even in heterogeneous environments [10].

From these measurements, the storage and loss moduli (G′ and G″, in Pa) can be estimated. The viscoelastic behavior of a material is characterized by these two moduli: the storage modulus G′ quantifies the elastic, solid-like component that stores mechanical energy, while the loss modulus G″ quantifies the viscous, liquid-like component that dissipates energy. When G″>G′, the material behaves predominantly like a fluid, whereas G′>G″ indicates a more solid-like response. Comparable values of G′ and G″ reflect mixed viscoelastic behavior typical of many biological systems.

Using this approach, we measured rheological properties in control and NMIIA-KO tissues. Consistent with our inference from the cell shape index, NMIIA-KO tissues exhibited reduced viscoelastic stiffness, indicative of increased deformability compared to control tissues (**Fig. 4f, g**).

4. Many figures e.g Extended Figure 6C, Fig 2D, Fig 2H, show a population of cells that stain very strongly for F actin. Are these tuft cells? These cells are noticeably smaller but similarly do not have "wiggly" boundaries with neighbouring enterocytes. Do these cell types also induce fractures? Can the authors speculate on why this might or might not be the case and provide more details on the role of these cells in maintaining the gut epithelium integrity?

We thank the reviewer for this interesting comment. Indeed, the cells displaying strong F-actin enrichment in Fig. 2D, 2H and previous Extended Data Fig. 6C correspond to tuft cells. Upon careful examination of the villus epithelium, we did not observe any fractures associated with tuft cells. Although tuft cells exhibit a rounded apical morphology like goblet cells, their apical surface area is notably smaller, and, more importantly, their cellular volume is significantly smaller. This difference in volume supports our hypothesis that fractures arise primarily from the mechanical pressure exerted by the large volume of goblet cells on their neighbouring enterocytes.

Tuft cells are rare chemosensory epithelial cells that play key roles in initiating type 2 immune responses, particularly during parasitic infections. They also contribute to epithelial homeostasis by sensing luminal signals and secreting signaling molecules like IL-25 and acetylcholine to coordinate immune and regenerative responses [11]. To our knowledge, no specific link has been

established between tufts cells and epithelial barrier integrity, which is consistent with our observation that they do not induce detectable junctional fractures under homeostatic conditions.

5. The laser ablation experiments need more explanations. In Extended Fig 8, laser cuts of G-E and surrounding E-E junctions show that E-E junctions recoil 4 times faster than G-E junctions. We were under the impression that G-E junctions would be under more tension due to the increased pressure from the GC cells. In contrast, the E-E junctions are "wiggly", which we thought would indicate that they are under less tension. Is this difference purely due to active myosin (as seen in increased P-MLC staining)? If myosin activity is inhibited, would the authors then expect the G-E junction to recoil faster due to the pressure exerted by GCs?

We thank the reviewer for this insightful comment and the opportunity to clarify the interpretation of our laser ablation experiments. Because Reviewer #3 also raised related concerns (see their comment #6), we choose to provide a common response to both comments below.

First, it is important to distinguish junctional morphology from junctional tension at the plane of ablation. While tight junctions can appear "wiggly", the laser cuts in previous **Extended Data Fig. 8** were performed in NMIIA-GFP explants and specifically targeted the AJ-associated NMIIA cable (see **Fig. 3j**), i.e., the peri-junctional actomyosin belt. At this apical AJ plane, junctions are typically straighter and mechanically governed by the contractile cortex, whereas the "bulky/rounded" appearance of goblet cells is most prominent below the AJs along the lateral membrane. This also explains the "non-bulky" appearance of the G–E junction in previous Extended Data Fig. 8: at the level of the adherens junctions, the goblet-cell perimeter is relatively narrow, whereas below the AJs goblet cells bulge and deform neighboring enterocytes (**Fig. 2a; Movie S1**). We propose that at the level of the AJs, the G–E interface is mechanically constrained by the polygonal actomyosin belts contributed by both the goblet cell and its neighboring enterocytes. Below the AJs, this constraint is reduced, allowing intracellular pressure and cell-shape forces to dominate and drive the rounded, bulky morphology (**Movie S1**).

Based on the rounded apical shape of GC cells and the enrichment of NMIIA at G–E junctions, we initially hypothesised that these junctions would be under higher tension than neighbouring E-E junctions. However, we observed that E–E junctions recoil approximately four-fold faster than G–E junctions following ablation (previous **Fig. 4e-g**). Importantly, this result is consistent with our phospho-MLC (pMLC) staining, which revealed low levels of active myosin at G–E junctions despite enrichment of total NMIIA (**Fig. 3j-k**), indicating that NMIIA accumulation at G–E does not necessarily translate into higher contractile tension at the AJ belt.

Line profile analyses further support this interpretation: G-E junctions exhibit minimal pMLC signal, particularly on the GC side, suggesting that the myosin network within GCs is largely inactive. In contrast, E-E junctions exhibit the characteristic "double-line" pMLC pattern associated with active contractile belts **(Fig. 3l, m)**. Thus, in general, junctional recoil correlates

more strongly with myosin activation state than with total myosin abundance or junctional morphology, in line with the reviewer's interpretation.

The E–E junctions originally analyzed in previous Extended Data Fig. 8 were assessed indiscriminately, without regard to their spatial relationship to goblet cells. We agree that spatial context is important and have now addressed this directly. We performed spatially resolved laser ablation experiments, distinguishing G–E junctions, first-neighbour E–E junctions (E–E1), and second-neighbour E–E junctions (E–E2) (new **Fig. 3n-p, Supplementary Movie S4-S6**). These experiments revealed that recoil velocity is ~10-fold higher at E–E2 junctions than at both G–E and E–E1 junctions, while G–E and E–E1 do not differ significantly. Consistent with this, pMLC levels are markedly lower at G–E junctions than at E–E1 and E–E2, indicating reduced myosin activity at G–E interfaces (new **Fig. 3m**). Notably, E–E1 and E–E2 junctions display comparable pMLC levels despite their strikingly different recoil velocities, indicating that gradients in actomyosin activation alone cannot fully explain the observed recoil behaviour.

Regarding the reviewer's question on myosin inhibition, it is important to emphasize that junctional laser ablation reports release of pre-existing junctional tension, not compressive or hydrostatic pressure exerted by neighbouring cells. Although goblet cells exert apical pressure on surrounding enterocytes, our data indicate that G–E junctions possess very low baseline tensile tension at the AJ plane (recoil velocity < 0.1 µm/s). Therefore, inhibiting myosin activity would not be expected to unmask faster recoil at G–E junctions, as there is little contractile tension to release in the first place. Instead, our data suggest that goblet-cell-induced pressure primarily shapes cell morphology below the AJs, whereas junctional mechanics at the AJ level are governed by the distribution of active junctional myosin (as reported by pMLC).

6. The authors argue that the stereotypical outward curvature of the G-E junction arises from GCs having higher pressure than enterocytes, leading them to hypothesize that GCs exert mechanical forces on their neighbours. From a physical perspective, this is intuitive and makes sense. However, when the authors treat mice with succinate to induce GC hypertrophy and increase pressure, the shape of the GC appears to change (Fig 2D), and G-E junctions no longer have the same distinct outward curvature, which might indicate that they may have lower pressure. This part is not very clear and a better explanation is needed. Can the authors give a reason why this might be the case? Is there some natural variation in this outward curvature, and does this correlate with GC size and fractures?

We thank the reviewer for this insightful observation. First, we would like to clarify that the apparent change in GC shape in Fig. 2D was due to image selection and is not representative of the overall GC population. Upon re-examining our data, we found that the cells shown in Fig. 2D represent a small minority of all GCs imaged (≈5%). We have now replaced this panel with a more representative image that better reflects the typical GC morphology.

We agree that there is some natural variability in the apical shape and curvature of G-E junctions, both in untreated and succinate-treated tissue (**Fig. R3**), which is also visible in Fig. 2D, I and Fig. 5D. However, we did not detect a systematic correlation between junctional curvature and the frequency or size of fractures. These apical shape variations could result from local mechanical heterogeneity, such as differences in cortical myosin contractility in neighbouring enterocytes or goblet cells, as well as from the presence and extent of junctional fractures themselves (which release the GC pressure)**.**

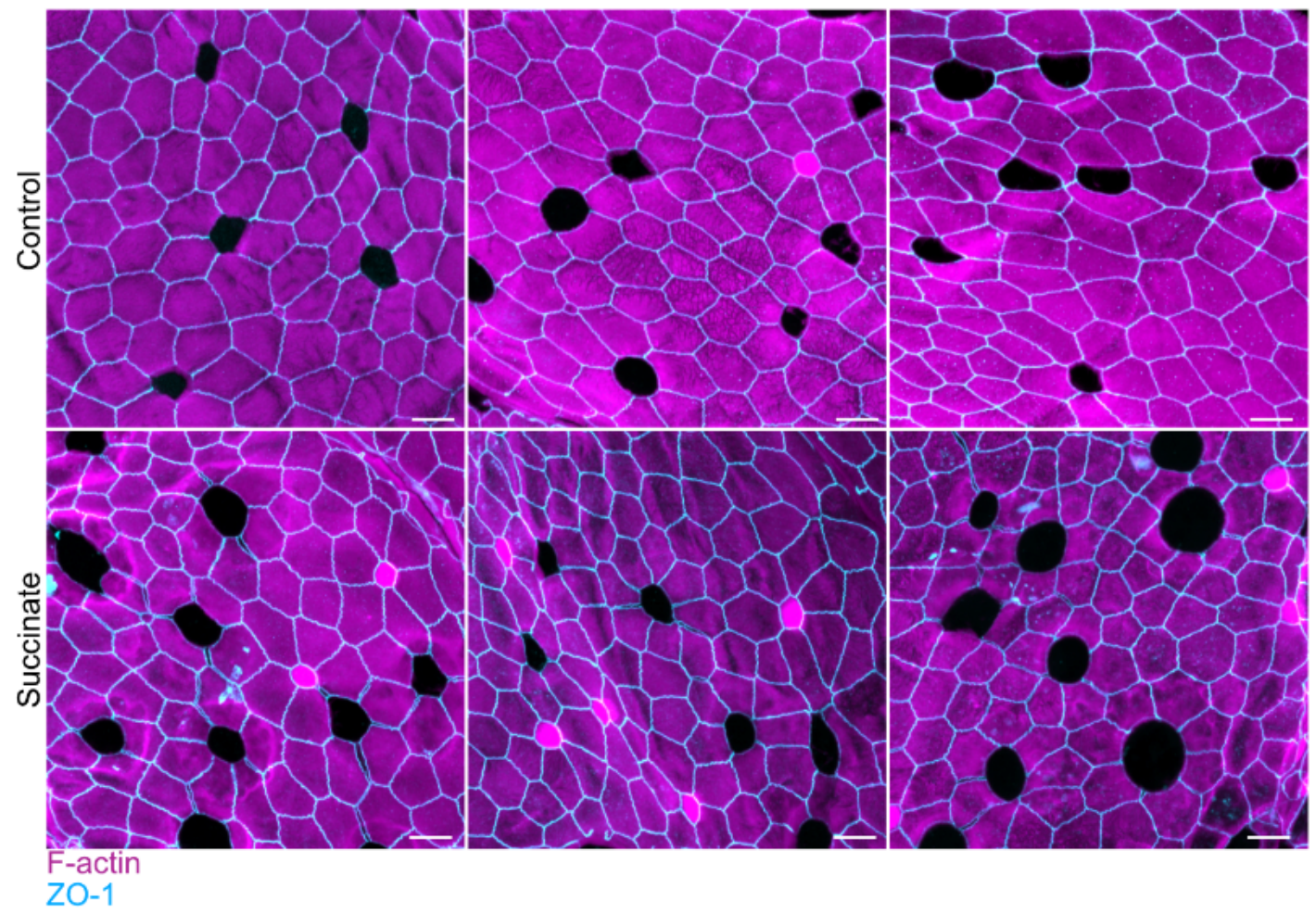

Fig. R3. Goblet cell exhibit variability in apical shape. *En face* images of control and succinate treated showing variability in the GC shape and apical curvature. Scale bar, 10 µm.

Importantly, the pressure exerted by goblet cells primarily originates from their large intracellular volume, which deforms the lateral walls of adjacent enterocytes along the apico–basal axis independently of the precise apical shape **(Fig. 2a)**. In other words, the outward curvature of the apical G–E boundary is not a direct readout of intracellular pressure.

Despite this variability in apical curvature, succinate-induced GC hypertrophy consistently increases GC volume and apical surface area, and this correlates robustly with an increase in epithelial fracturing (quantified in **Fig. 2e**). Together, these observations support our interpretation that increased GC volume and associated intracellular pressure, rather than apical curvature per se, drive junctional disruption.

7. From our understanding, Villin:Cre will also drive knockout of NMIIA in Goblet cells, which may be more affected by this, given the higher levels of NMIIA-GFP in goblet cells. From Fig 4E, it appears that GCs also look slightly bigger in the KO (although we acknowledge it is non-significant in Extended Data Fig 9E). Can the authors disentangle the effect of a NMIIA KO in GCs from enterocytes? Would they expect a loss in GAFs as well in a GC-specific knockout e.g. Muc2-Cre line?

We agree with the reviewer that dissecting the respective contributions of NMIIA in goblet cells and enterocytes is important for interpreting the phenotype.

First, as noted by the reviewer, in Villin^{CRE};NMIIA$^{Fl/Fl}$ the apical area of GCs is not significantly altered upon the loss of NMIIA (previous Extended Data Fig. 5E, Fig 4D). This is consistent with our earlier observation that, despite strong enrichment of total NMIIA at the G–E interface, goblet

cells show very low levels of active myosin (pMLC) at their apical ring, indicating limited intrinsic contractility at this site.

To directly disentangle the effect of NMIIA loss in GCs versus enterocytes, we generated during this revision a new mouse line, Muc2-CreERT2; *Myh9*$^{fl/fl}$, which enables inducible, goblet-cell-specific depletion of NMIIA while preserving NMIIA expression in enterocytes **(Fig. R4**). These data are now included in the revised manuscript as **Supplementary Fig. 6**.

NMIIA immunostaining reveals a strong apical ring in goblet cells of control mice and a complete loss of this signal in goblet cells of Muc2-CreERT2; *Myh9*$^{fl/fl}$ mice, while apical and basolateral NMIIA signal in neighbouring enterocytes is preserved. This validates both the efficiency and specificity of the GC-targeted knockout.

These results indicate that NMIIA activity within goblet cells is not required for fracture formation. Instead, they support our interpretation that junctional fractures primarily arise from the mechanical and rheological properties of the surrounding enterocyte network, regulated by myosin II–dependent contractility, rather than from actomyosin activity within goblet cells themselves.

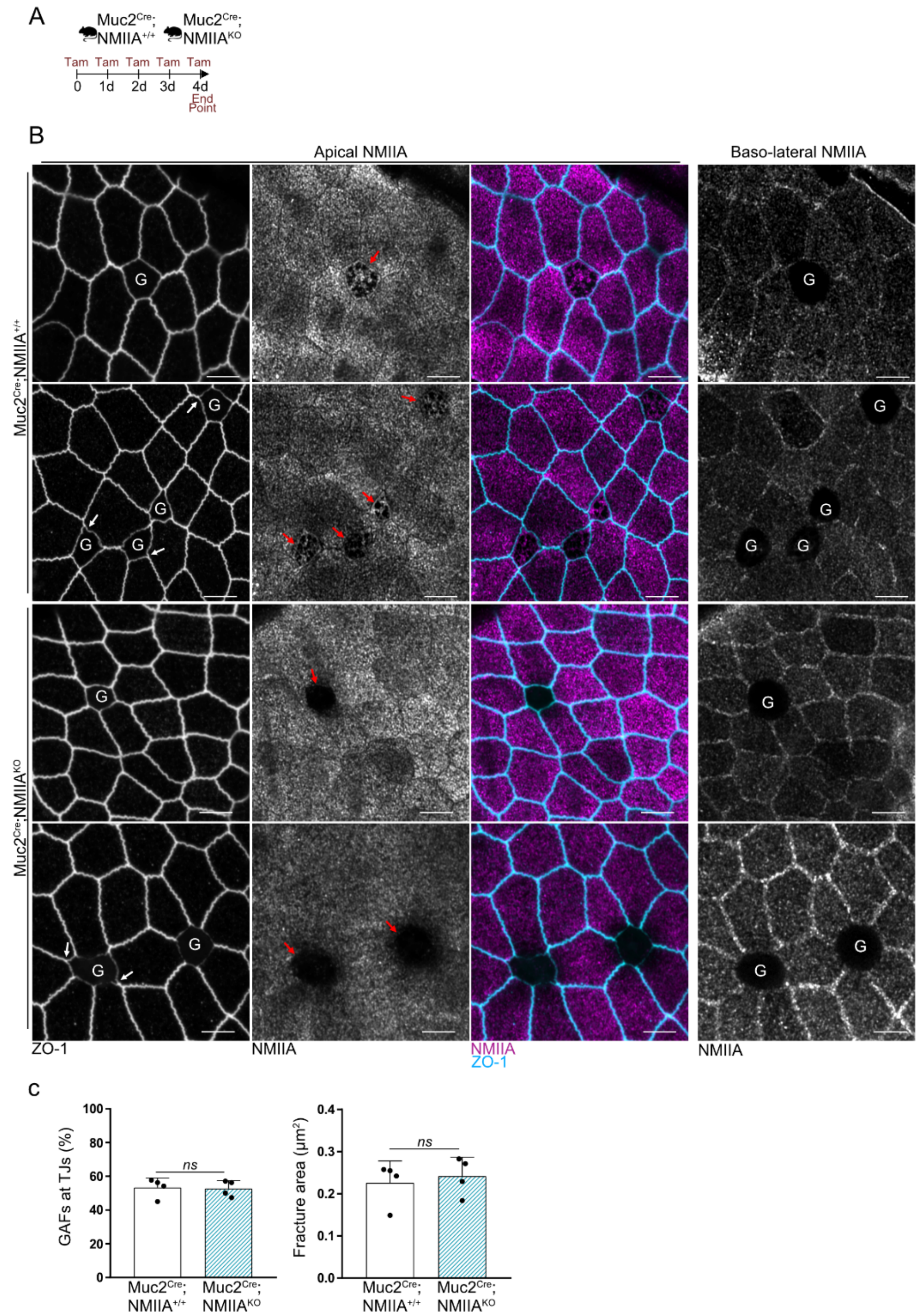

Figure R4. Specific loss of NMIIA in goblet cells does not affect GAFs formation. (A) schematic representation of experimental procedure for Muc2-CreERT2; NMIIA-KO. (B) Representative high resolution *en face* images from control and Muc2-CreERT2; NMIIA-KO mice. White arrows show GAFs and red arrows show NMIIA ring in goblet cells. Scale bars, 5µm. (C) Bar charts displaying the average percentage of GAFs at TJs (left) and average fracture area (right). Student t-test, ns=non-significant, $p>0.05$; N=4.

8. The paper generally references fractures at the level of TJs, AJs, and Desmosomes. Can the authors provide a high-resolution Apical-Basal or Basolateral cross-section image to show how these fractures appear at these sites? Further explanation on how the authors distinguish between fractures at the level of TJs and AJs would also be helpful.

We thank the reviewer for this helpful suggestion.

Fractures at different junctional levels were distinguished using immunostaining with junction-specific markers: ZO-1 and Occludin for TJs, E-cad for AJs and desmocollin for desmosomes.

Previously, due to technical limitations, we imaged each junction type separately. The most reliable antibodies for ZO-1 and E-cadherin are both rat-derived, which precluded co-staining, and our *en face* imaging approach – required for visualizing fracture patterns within the epithelial plane – does not allow simultaneous apico-basal visualization.

As requested by the reviewer, we have now performed high-resolution apico-basal imaging of TJs and AJs using Occludin/E-cadherin and ZO-1/E-cadherin co-staining (enabled by a newly available, mouse-raised ZO-1 antibody) and generated orthogonal projections to visualize both junctions along the z-axis **(Fig. R5)**.

This dual-staining approach confirms our earlier conclusions obtained from separate imaging: tight junctions fracture more frequently than adherens junctions. Notably, fractures detected using Occludin or ZO-1 are often not accompanied by corresponding fractures at the AJ level, supporting the view that epithelial fractures initiate at the apical TJ and may subsequently propagate basally toward AJs and desmosomes during goblet cell hypertrophy.

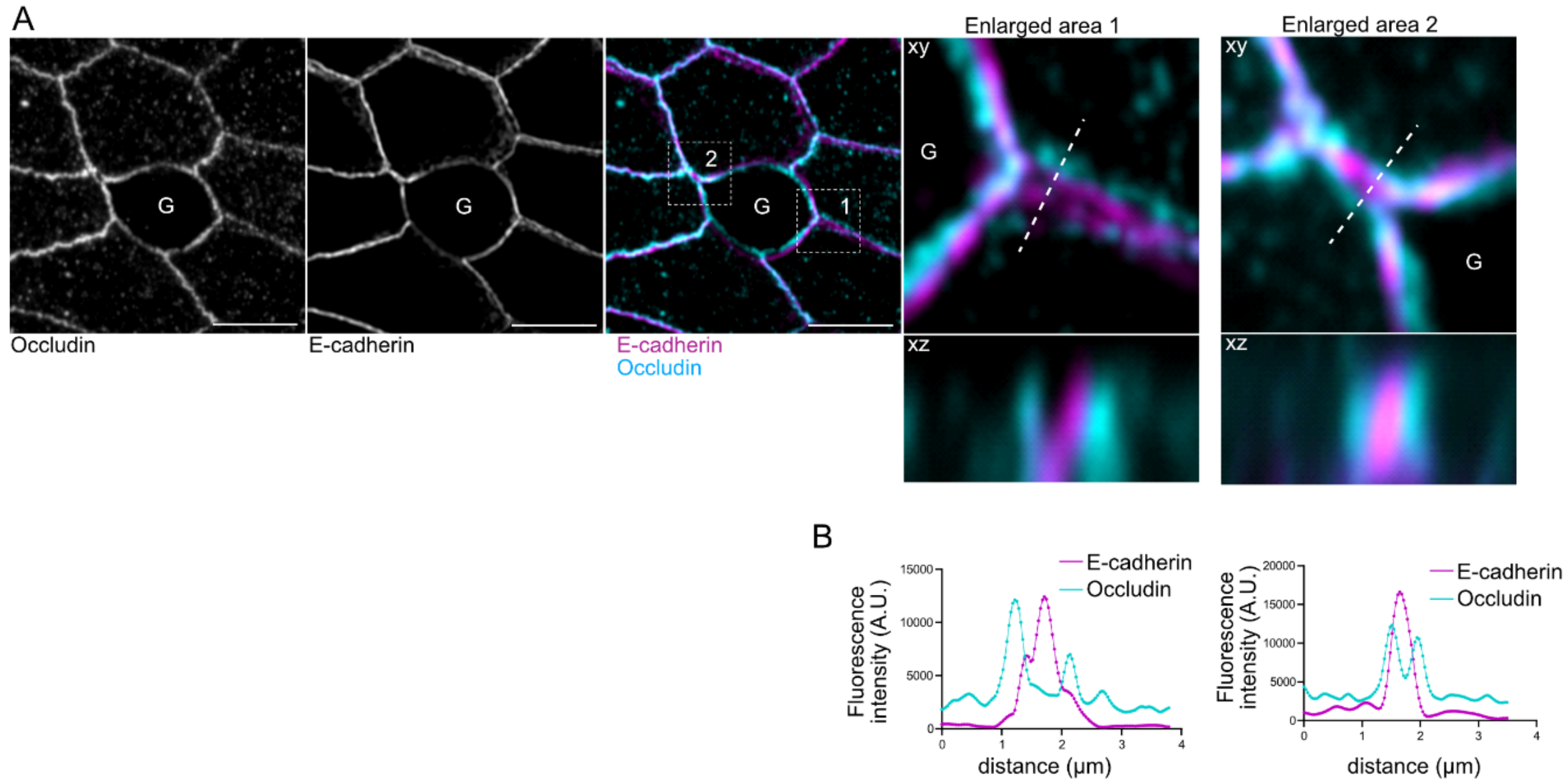

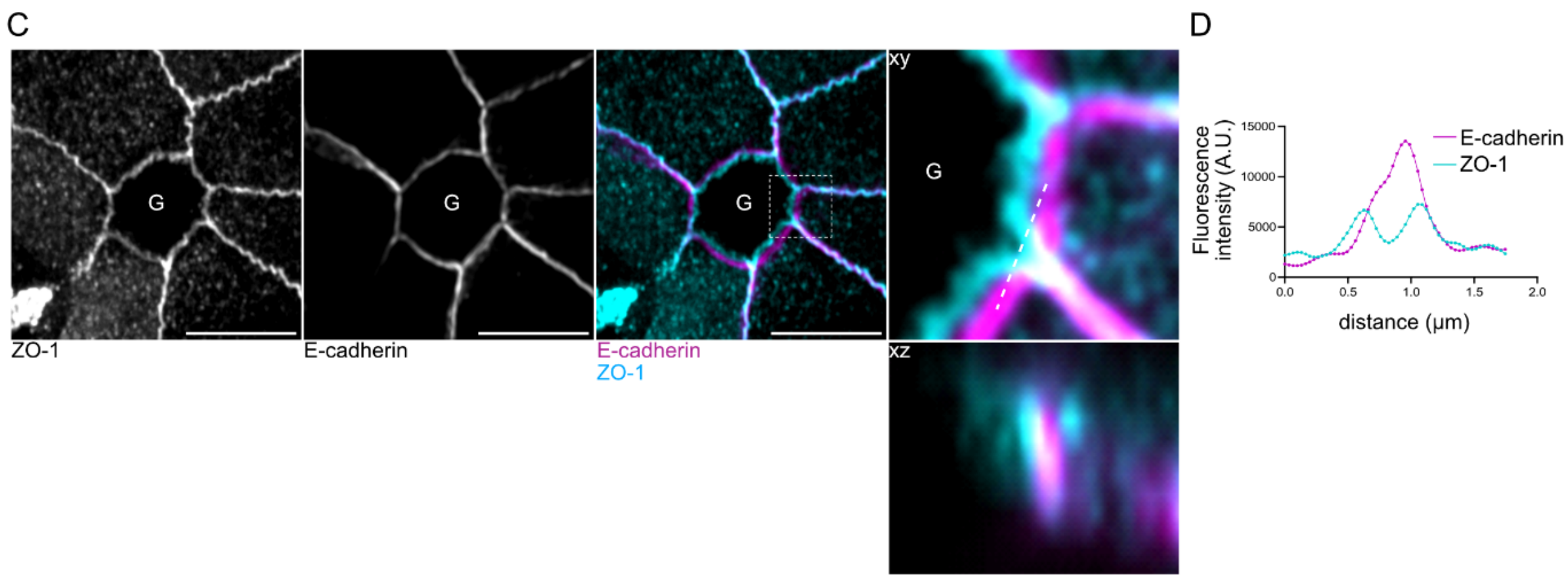

Fig. R5. Fractures originate at the TJ level. (A) Representative *en face* image of murine small intestinal tissue co-stained for TJs and AJs; maximum intensity projection (Z range, 0.8 µm) and orthogonal views (xy and xy) of boxed regions shown in higher magnification. (B) Corresponding line intensity profiles for Occludin (cyan) and E-cad (magenta) at tricellular junctions, along the line indicated in (A). (C) Representative *en face* image of murine small intestinal tissue co-stained for TJs and AJs; maximum intensity projection (Z range, 0.6 µm) and orthogonal views (xy and xy) of boxed region shown in higher magnification. (D) Corresponding line intensity profiles for Occludin (cyan) and E-cad (magenta) at tricellular junctions, along the line indicated in (C).

9. The tissue-scale fractures that form in response to shear stress challenge (Fig. 2H; Extended Data Fig. 6C) are striking and are clear hallmarks of mechanical failures. Can the authors provide (if possible) some videos or images of the fracturing process? Further, are NMIIA KO mice less susceptible to tissue-scale fracturing in response to shear, as GAFs are smaller?

We fully agree with the reviewer that direct visualization of the fracturing process would be highly informative, especially during the shear stress experiment.

Unfortunately, due to technical constraints, it is not currently feasible to perform live imaging of tissue fracturing during the luminal shear stress assay. In this setup, we apply fluid flow directly through the gut lumen *ex vivo*, which makes it technically challenging to image the villus epithelium in real time, as the epithelial surface is enclosed within the tubular intestinal structure.

To nevertheless visualize the fracturing process, we performed an alternative *ex vivo* assay in which intestinal explants were treated with EDTA to partially destabilise junctions. As shown in the accompanying video (**Video R1**) and **Fig. R6,** this approach allowed us to monitor the onset and propagation of epithelial fractures in real time under the microscope. Consistent with our conclusions, fractures reproducibly initiated at G–E–E tricellular junctions, confirming that goblet cells act as mechanical weak points within the epithelium.

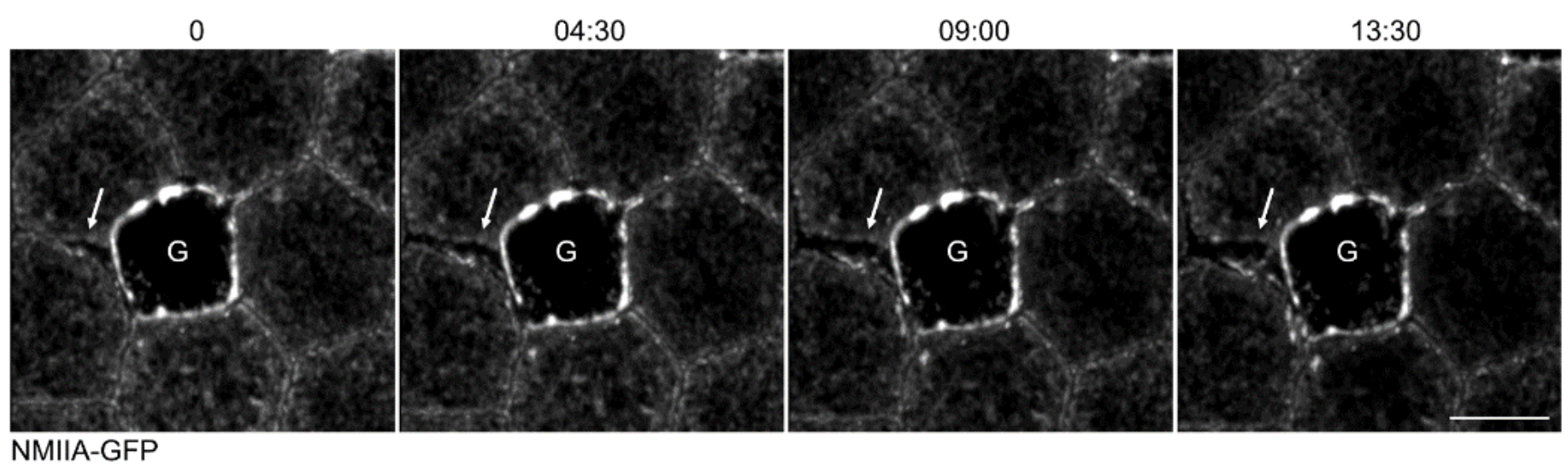

Fig. R6. **Live imaging of junctional fracture initiation at G–E–E tricellular junctions**. Representative time-lapse montage showing the onset and progression of junctional rupture in the presence of EDTA. Fracturing initiates at G–E–E tricellular junctions and propagates along neighboring junctions. Scale bar, 5 μm.

Regarding the role of NMIIA, we have now assessed, in the course of this revision, the response of NMIIA-KO tissue, with and without succinate treatment, to the *ex vivo* shear stress assay. Consistent with our previous findings **(Fig. 2h)**, control tissue remained largely unaffected by shear stress, whereas succinate-induced goblet cell hypertrophy led to pronounced tissue-scale fracturing. In contrast, NMIIA-KO tissue did not exhibit large-scale fractures in response to shear stress, and notably, the hypertrophy-induced susceptibility to shear stress was abolished in NMIIA-KO mice, with no major fracturing observed following shear challenge **(Fig. R7)**.

These results highlight the importance of tissue-scale mechanical properties in governing fracture formation under ex vivo mechanical challenge. Together, they support our conclusion that goblet cell hypertrophy – and the associated increase in intracellular volume and pressure – renders the epithelium more fragile and prone to mechanical failure, whereas reduced actomyosin contractility in NMIIA-KO tissue confers increased resistance to shear-induced tissue-scale fracturing.

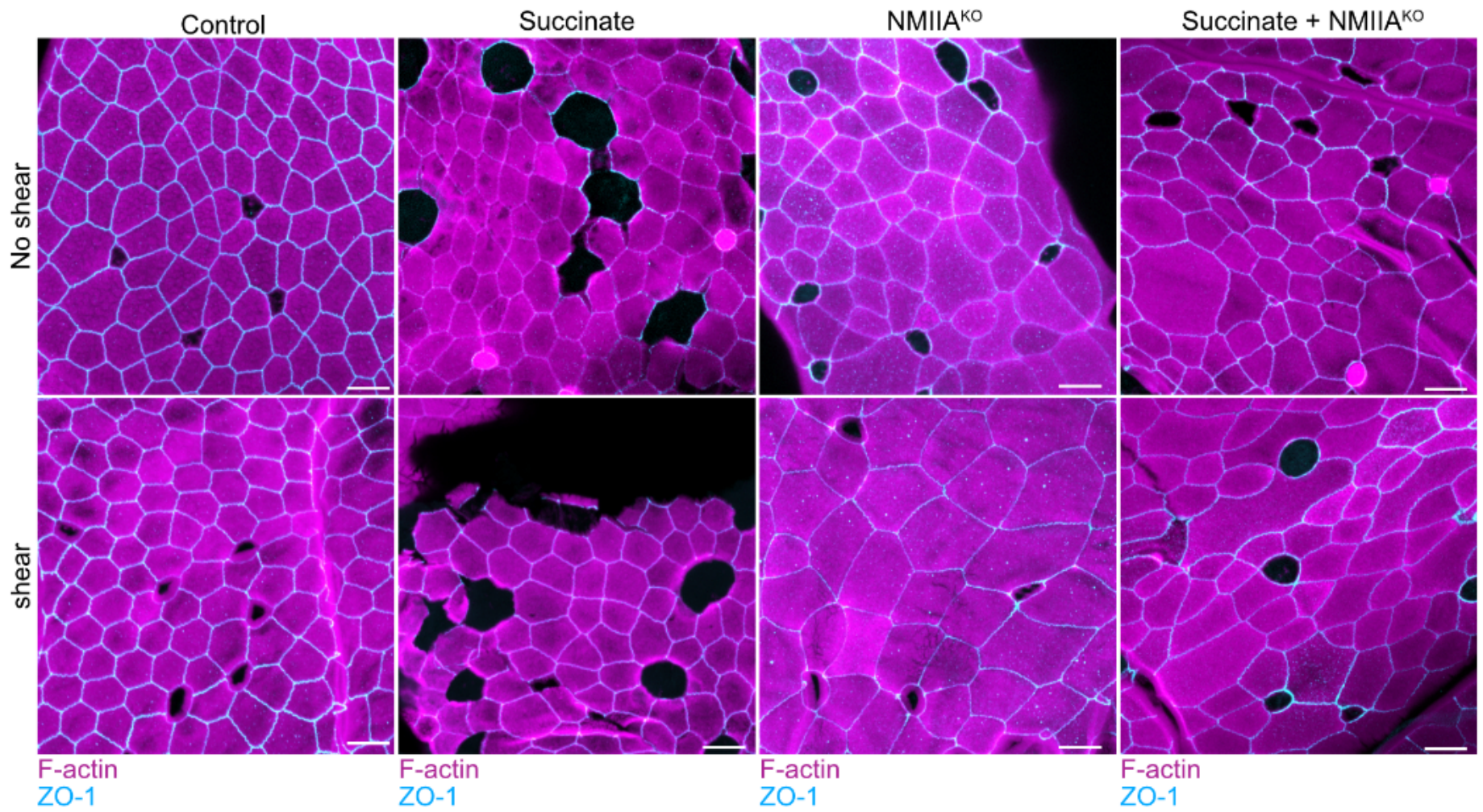

Fig. R7. **Shear stress response in NMIIA-KO tissue.**
Representative *en face* images from control and NMIIA-KO intestinal tissues with and without succinate treatment subjected to luminal shear stress; shown as maximum intensity projections (Z-range: 1.8–6.4 µm). Scale bars, 10 µm.

Minor Comments:

1. Fig. 2A zoomed region is slightly different from the box

We apologize if we missed anything, but would like to clarify that there is no zoomed region in Fig. 2A. Nevertheless, we have carefully re-examined all figures containing zoomed regions and did not identify any discrepancies between the boxed areas and their corresponding zooms.

2. "unrecognized mechanical impact of goblet cells in destabilizing epithelial cohesion and compromising barrier function, highlighting the importance of cellular heterogeneity." Line 33 Slightly Clunky writing, makes it sound like compromised barrier function is desirable

We thank the reviewer for the comment. The sentence has been revised for clarity to avoid any unintended implication that barrier compromise is desirable. It now reads: "Our findings reveal a previously unrecognized mechanical role of goblet cells in weakening epithelial cohesion and compromising the intestinal barrier, highlighting the importance of cell heterogeneity in maintaining tissue integrity."

3. Typo: Extended Data Figure 1D labelled as 1C. As above, it would also be helpful to see some images of fractures at AJs and Desmosomes.

We thank the reviewer for noticing this error. The labeling of Supplementary Fig. 1D has been corrected. In addition, representative images of fractures at adherens junctions (AJs) and desmosomes have been added to Supplementary Fig. 1D and 1E, respectively, as requested.

4. The Large intestine has a denser proportion of goblet cells. What do we expect to see in terms of TJ fractures? Can the authors speculate on how the large intestine deals with this mechanical challenge?

We thank the reviewer for this interesting question. Indeed, the large intestine contains a higher density of goblet cells compared to the small intestine. To assess whether goblet cells also induce fractures in this region, we examined colonic tissue and we did not observe any GAFs in the colon.

Upon further analysis, we noticed distinct morphological differences between the colonocytes and small intestinal enterocytes **(Fig. R8)**. Colonocytes appeared more irregular in shape and less tightly packed than enterocytes. To quantify this, we measured the cell shape index and found that it is significantly higher in the colon than in the small intestine, suggesting increased epithelial deformability.

These observations support our hypothesis that enhanced epithelial deformability may buffer mechanical stress and prevent fracture formation, despite the high density of goblet cells.

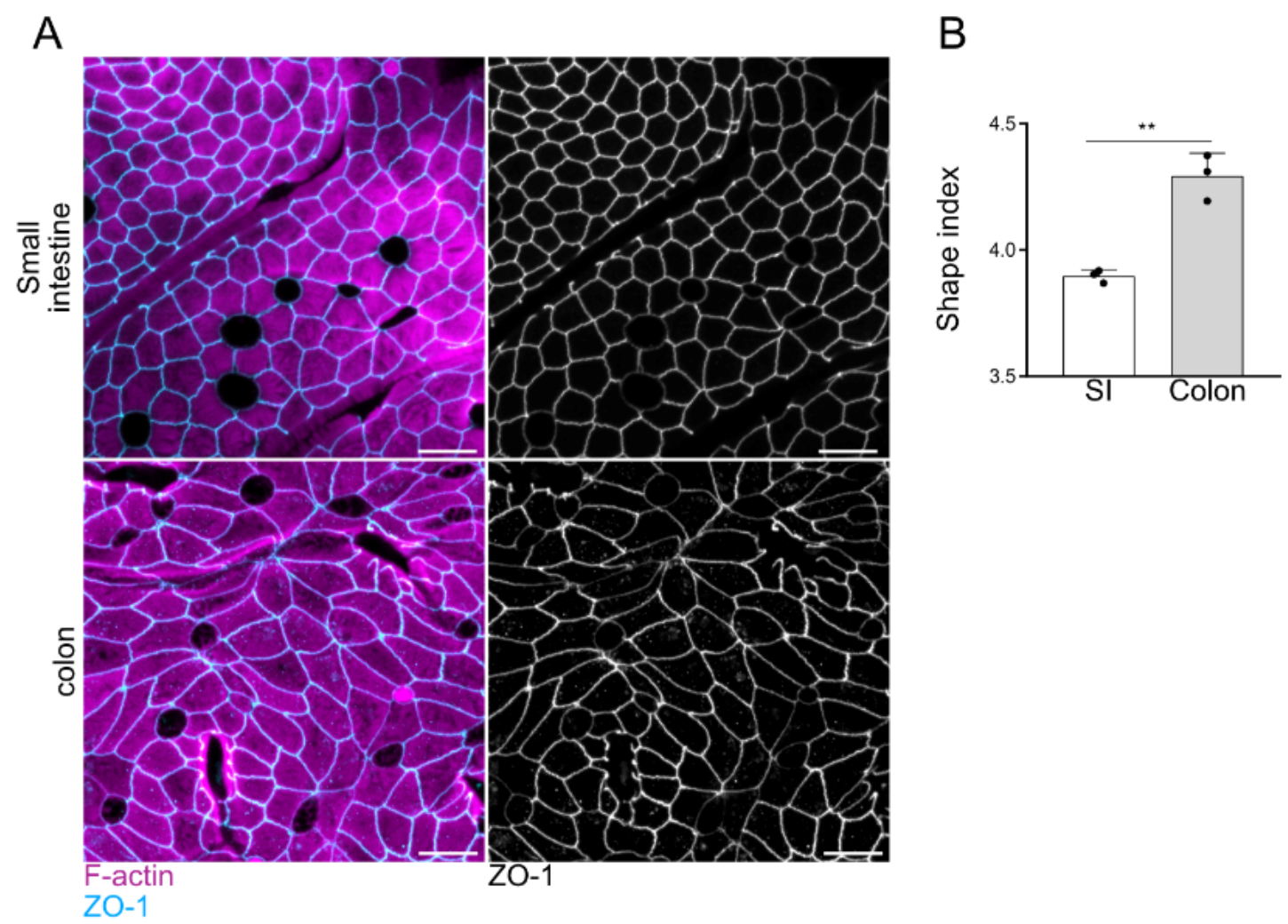

Fig. R8. Enhanced epithelial deformability in the colon prevents GAF formation despite high goblet cell density. (A) Representative *en face* images of small intestinal and colonic epithelia showing the irregular, less tightly packed morphology of colonocytes compared to enterocytes. Scale bars, 10 µm. (B) Bar chart showing average cell shape index of enterocytes in the small intestine (SI) and colonocytes in the colon. N=3.

5. It would be helpful for the general audience if the terms in Extended Fig 3. D-F are briefly explained/defined in the legends (e.g. curvilinear coordinates s and θ, as defined in the supplementary methods)

We thank the reviewer for this helpful suggestion. The legend for Supplementary Fig. 3D–F has been modified to include brief definitions of the terms s and θ, as described in the Supplementary Methods.

6. The authors have rigorously quantified their data, which is commendable. While we appreciate potential space constraints, in some cases such as Extended Fig 1D, Extended Fig 3I, Fig 2F, it will be helpful for the reader to see the qualitative data as well.

We thank the reviewer for this positive feedback and constructive suggestion.

- Supplementary Fig. 1d: Representative images of adherens junctions (AJs) and desmosomes have been added
- Supplementary Fig. 3i: The corresponding qualitative images are now presented in Fig. 2d
- Fig. 2f: Unfortunately, no qualitative images are available for this assay. We apologize for not having clearly described the experimental principle. The intestinal permeability assay (gavage with 4kDa FITC-dextran) is a standard *in vivo* assay for gut paracellular permeability. As detailed in the Methods, mice were gavaged 4 kDa FITC-dextran and blood was collected two hours later for spectrofluometric quantification of FITC-dextran concentration in the serum. Therefore, this assay yields quantitative but not image-based data.

7. For Fig 4L-N, statistical comparisons between NMIIAKO and NMIIAKO succinate should also be performed, and p values should be given; this should similarly be performed for Extended Fig 11.

We thank the reviewer for this helpful suggestion. The requested statistical comparisons have been added, and p-values for the statistical comparisons between NMIIA-KO and NMIIA-KO succinate are now reported for Fig. 4L–N (**Fig. 5e-g** in the revised version) and previous Extended Data Fig. 11 (**Supplementary Fig. 8** in the revised version).

8. Typo in line 616 (should be perimeter/area instead of perimeter/)

We apologize for this error and have corrected it (line 746 in the revised version).

9. Typo in Extended Figure 9A legends (Right and Left swapped)

We apologize for this oversight and have corrected the labeling in the legend of previous Extended Data Fig. 9A (**Supplementary Fig. a** in the revised version).

Reviewer #2 (Remarks to the Author):

I co-reviewed this manuscript with one of the reviewers who provided the listed reports. This is part of the Nature Communications initiative to facilitate training in peer review and to provide appropriate recognition for Early Career Researchers who co-review manuscripts.

Reviewer #3 (Remarks to the Author):

I have reviewed the paper by Creff et al. The paper studies the impact of goblet cells in the stability of the intestinal barrier. They combined in vivo and ex vivo experimental data together with modeling approaches to understand the mechanical role of goblet cells in the intestine. They found that the mechanical pressure induced by goblet cells leads to the local fracture of tight junctions at the interface with the neighboring enterocytes. I found the paper of interest for the readers of Nature Communications. However, there are a few points that the authors need to address before publication.

1. An easy one. The paper includes only four main figures and 12 extended (supplementary) figures. To my knowledge, Nature Communications does not impose strict limits on the number of main figures. I therefore recommend that the authors reorganize the figures to incorporate some of the key data currently placed in the supplementary section into the main figures. This will reduce the need for readers to constantly switch between the main text and the supplementary material. Important elements, such as data on the model, laser ablation, and others, should be highlighted more prominently in the main text.

We thank the reviewer for this helpful suggestion. We agree that some important elements were previously placed in the extended figures and that the supplementary section was quite extensive. To address this, we have reorganized the figures by increasing the number of main figures from 4 to 5 and reducing the number of supplementary figures from 12 to 8.

2. The maintenance of epithelial integrity is crucial. So, under physiological conditions, the functional role of the fractures induced by goblet cells remains unclear. I believe the authors should further discuss this aspect to clarify its biological significance.

We thank the reviewer for raising this important point. We agree that the physiological role of goblet cell–associated fractures under homeostatic conditions remains unclear. We have now expanded the Discussion to explicitly address this issue, acknowledging this limitation and discussing potential biological interpretations. Specifically, we now emphasize that, under homeostatic conditions, GC-associated fractures are not sufficient to trigger overt pathology and may represent transient, repairable mechanical failures that locally dissipate stress arising from cellular heterogeneity, rather than pathological barrier breakdown. We also discuss that this compensatory mechanism appears to have limited capacity, becoming insufficient under conditions of goblet cell hypertrophy or increased mechanical challenge. Further work will be required to determine whether these events are adaptive or represent unavoidable mechanical trade-offs of goblet cell function.

3. In Figure 2D, the rupture or discontinuities in ZO-1 staining (as described in Figure 1) are not clearly visible. The ZO-1 signal appears continuous, including at the tricellular junctions, under

both control and succinate-treated conditions. The authors should clarify this apparent discrepancy and explain how it aligns with their earlier observations.

We thank the reviewer for this important observation. We agree that the ZO-1 signal in the original Fig. 2D could be misleading and have therefore replaced this panel with a more representative image.

To clarify, we define goblet-associated fractures (GAFs) by the separation of the two enterocyte–enterocyte (E–E) bicellular junctions at the G–E–E tricellular junction, which necessarily disrupts TCJ integrity. In the original image, although the E–E junctions were clearly separated, residual ZO-1 signal remained associated with the goblet-cell cortex in the TCJ region (**Fig. R9)**. This residual signal does not indicate an intact TCJ, as the defining geometric feature of a TCJ – the convergence of three cell membranes at a single vertex – is lost once the E–E junctions separate.

Importantly, this criterion is consistent with our analysis in Fig. 3E, showing GAF association with G-E-E TCJ fractures, which are marked by disorganization and loss of the characteristic tricellulin enrichment at tricellular vertices. Thus, when E–E junctions become fully separated (**Fig. R9** white arrows), the TCJ is necessarily breached, even if some ZO-1 signal persists locally at the goblet-cell membrane. Moreover, the gap between the separated E–E junctions is negative for brush-border (F-actin) staining (**Fig. R9**), directly indicating an open paracellular space and a barrier breach.

We hope that this clarification and the revised image resolve the confusion and make the junctional features in Fig. 2D consistent with those described in Fig. 1.

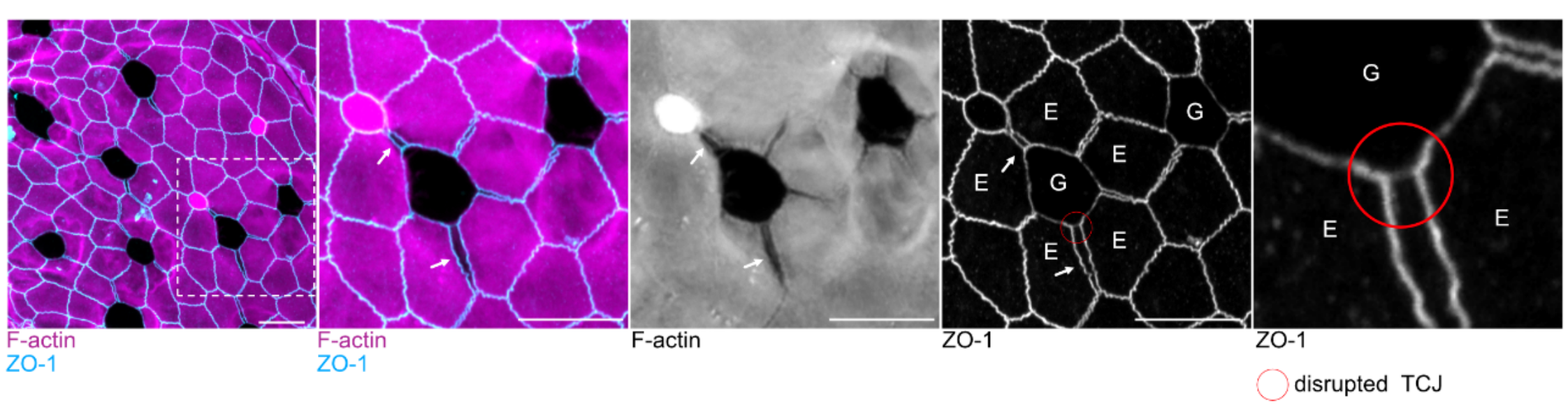

Fig. R9. Fractures at tricellular junctions under succinate treatment.
En face images of villus epithelium showing fractures in E–E tight junctions (TJs) adjacent to goblet cells, visualized by ZO-1 and F-actin staining. White arrows indicate fractures in E–E1 TJs. The zoomed view highlights the G–E–E tricellular junction (TCJ), showing residual ZO-1 signal along the GC membrane and the disruption of the TCJ into multiple vertices. Scale bars, 10 µm.

4. Figure 2F (dextran incorporation) appears to be the only experiment directly assessing barrier function. However, it is not well described in the text. The authors should provide a clearer explanation of the experimental setup and include both quantitative measurements and representative images to support their conclusions.

As noted in our response to the first reviewer comment, the intestinal permeability assay (oral gavage with 4 kDa FITC–dextran) is a well-established and widely used method to assess *in vivo* paracellular barrier function. To our knowledge, it is the only assay that allows direct functional evaluation of epithelial permeability *in vivo*.

We apologize for not having clearly described the experimental principle in the initial version. As detailed in the Methods, mice were gavaged with 4 kDa FITC–dextran, and blood was collected two hours later. FITC–dextran concentration in the serum was then quantified by spectrofluorometry (**Fig. R10**). This assay thus provides a quantitative measure of gut barrier permeability, but it does not generate representative images, as it relies on systemic fluorescence measurements rather than microscopy.

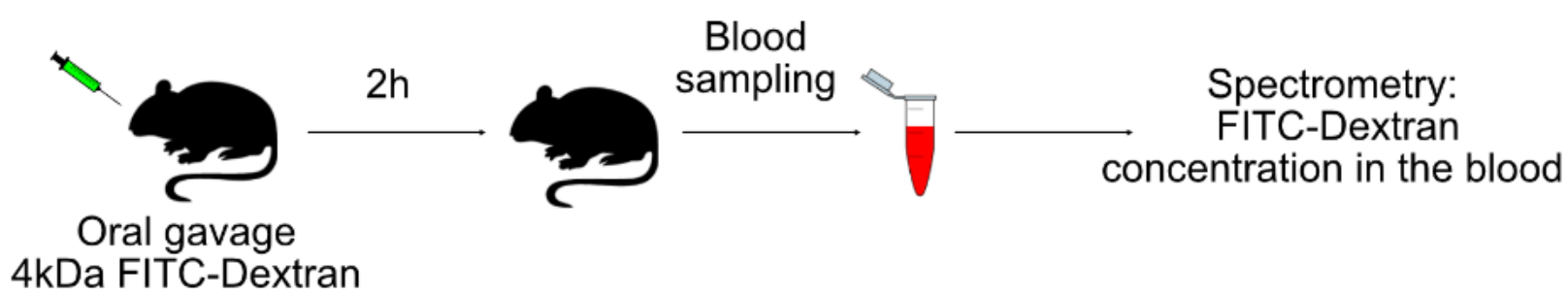

Fig. R10. Schematic representation of the intestinal permeability assay

5. The authors use a peristaltic pump to increase the pressure inside the tissue and report an increase in shear stress of 1 Pa. However, there may also be a contribution from normal stress (i.e., luminal pressure). It appears that the projected area of the cells increases following flow application. Could this change be driven by normal stress rather than shear stress? Would it be possible for the authors to estimate the magnitude of the normal stress in their system?

We thank the reviewer for this important point. We agree that luminal (normal) pressure could contribute to the mechanical challenge in addition to shear stress. To estimate the possible magnitude of this contribution, we performed a simple calculation based on the pressure generated by our peristaltic pump setup.

Parameters and assumptions

- Temperature = 20°C: viscosity μ = 1.0 mPa.s, density ρ = 1000 kg/m^3.
- Total flow rate Q = 23 mL/min = 3.833×10^{-7} m^3/s
- Tube dimensions (intestine tube) : length L = 0.05 m, inner diameter D = 1.5 mm
- We assume steady laminar flow and use Hagen–Poiseuille flow in a cylindrical tube. The pressure drop along the Tube length can be described as follows:
-

$$\Delta P = \frac{128\mu L Q}{\pi D^4}$$

- Key intermediate values

- Mean flow velocity: $v = \frac{Q}{\pi(\frac{D}{2})^2} \approx 0.217 m.s^{-1}$
- Reynolds number: $Re = \frac{\rho v D}{\mu} \approx 365$, consistent with laminar flow.

- The pressure drop along the tube gives an estimation of the normal pressure using the Hagen-Poiseuille formula above yields:

$$\Delta P \approx 154\, Pa\ (1.54 mbar)$$

As the hydraulic resistance of the system is dominated by the cell-covered tube, this pressure drop provides an upper-bound estimate of the luminal pressure – and thus of the normal stress – experienced by the epithelium along the tissue. In our system, this value is estimated to be approximately 154 Pa, which is relatively modest (for example, in blood vessels normal pressure varies from ≈1.3 kPa in the vein to ≈16 kPa in the aorta[12]). Moreover, considering the three-dimensional geometry of the intestinal epithelium – with villi protruding into the lumen and moving with the flow – the tissue is expected to be more sensitive to shear forces than to normal pressure.

To further assess the mechanical response, we examined E-cadherin localization at adherens junctions, as E-cadherin is known to undergo junctional reinforcement in response to tensile strain[13]. We did not observe detectable E-cadherin reinforcement following flow application (**Fig. R11A, B**), which is consistent with the notion that pressure-induced epithelial stretch is limited in our system, although we do not exclude minor contributions of normal stress.

We also acknowledge that in the original Fig. 2H, the cell area in the displayed image appeared increased following shear stress. In response to the reviewer's concern, we performed a

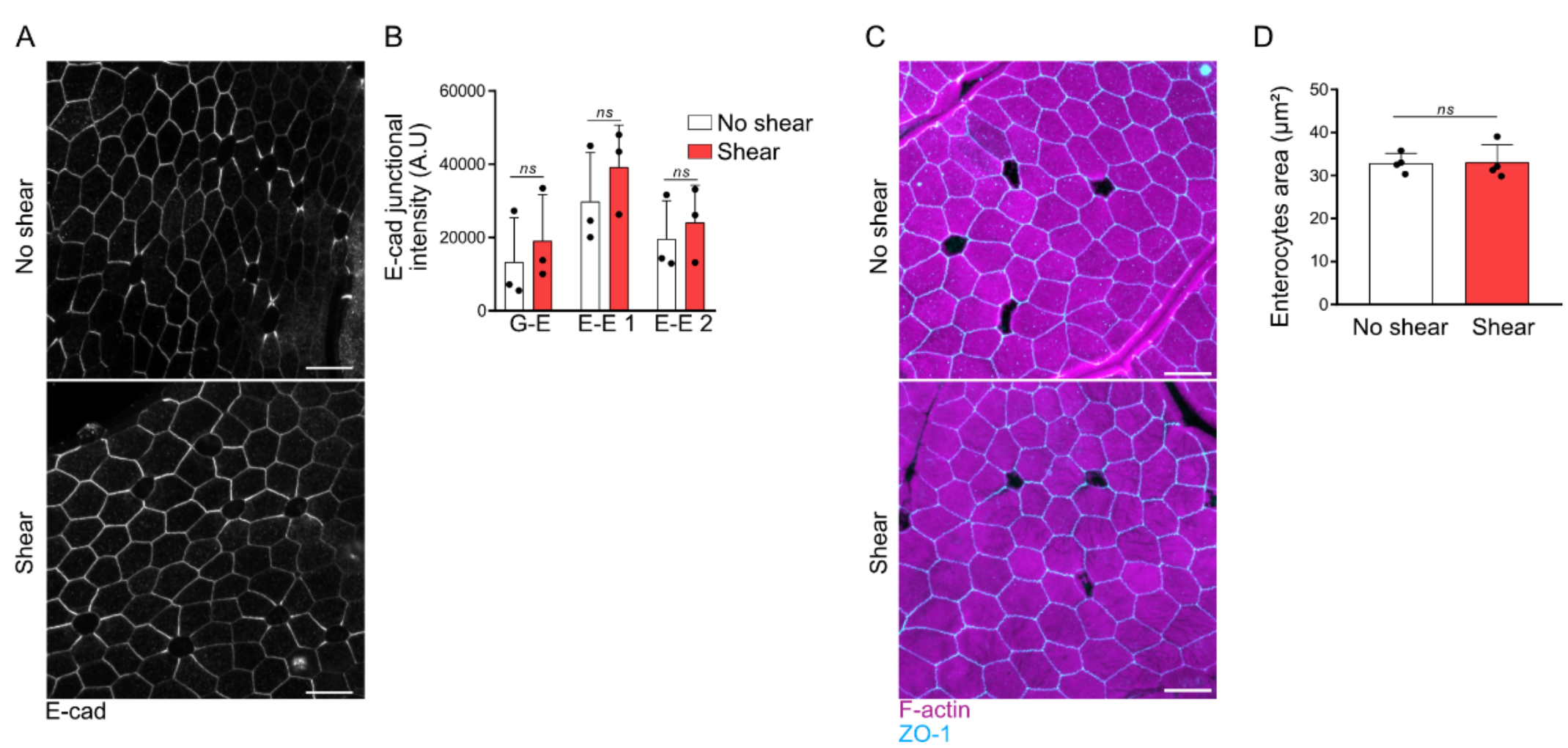

Fig. R11. (A) Representative *en face* images of control tissues submitted to luminal shear stress; maximum intensity projection (Z range: 2.4-3.2 µm). (B) Bar charts displaying average E-cadherin junctional intensity. Multiple t-test; ns: non-significant. (C) Representative *en face* images of control tissues submitted to luminal shear stress, maximum intensity projection (Z range: 3.2-4.2 µm). (D) Bar chart showing the average enterocyte area. t-test, ns: non-significant.

quantitative analysis across multiple samples and found that enterocyte apical area does not significantly change upon shear stress (**Fig. R11D**), indicating that the original image was not representative of the full dataset. Epithelial cell size exhibits substantial natural variability, which can give a misleading impression when single fields of view are compared. These quantitative data have now been added as **Supplementary Data Fig. 4d**. To avoid confusion, the images have been replaced with examples that are more representative of the overall dataset (**Fig. 2f; Fig. R11C**).

Taken together, although we cannot fully exclude a contribution from luminal pressure, both our quantitative estimate and experimental analyses indicate that the principal mechanical challenge in our assay arises from fluid flow–induced shear stress rather than pressure-driven stretching of the epithelium.

6. In the laser ablation experiments comparing tension at G–E and E–E junctions, it would be valuable to also include E–E junctions that are in contact with goblet cells. It is currently unclear which E–E junctions are being probed. Are they located near goblet cells or not? One would expect G–E junctions to be under tension, but the E–E junctions shown to fracture exhibit a wavy morphology (see ZO1 staining fig 1), which may suggest compression rather than tension. Additionally, the example shown in Extended Fig. 8 for a G–E junction does not appear to feature a clearly bulging goblet cell, which could influence the measurements. These points require clarification.

As discussed above, Reviewer #1 raised closely related concerns (see comment #5). Because both comments address the interpretation of our laser-ablation measurements and the spatial context of the probed junctions, we provide a unified explanation in our response to comment #5. We kindly refer the reviewer to that section for a detailed clarification of which E–E junctions were ablated, how junctional tension varies with distance from goblet cells, and how junction morphology relates to local mechanical forces.

7. The authors frequently refer to tissue fluidity (e.g., line 238), but it remains unclear whether actual cell–cell rearrangements were observed. This point needs to be clarified and, ideally, directly measured. Under the current culture conditions, I do not believe that the shape index alone is sufficient to infer fluid-like behavior in the tissue.

We thank the reviewer for this important comment. As noted in our response to Reviewer #1 (comment 3), we used the cell shape index to infer tissue fluidity, following a previously established and experimentally validated framework in which a critical threshold in this parameter marks a transition between solid-like and fluid-like tissue states – the so-called jamming–unjamming transition[4–8].

We agree, however, that the term *tissue fluidity* may have been misleading in our context. In this study, we do not aim to demonstrate tissue flow or active cell–cell rearrangements. Rather, we focus on epithelial mechanical stiffness (or deformability). Our data indicate that enterocyte

mechanical properties are critical for junctional fracturing. Under physiological conditions, enterocytes display regular hexagonal packing. Upon myosin II depletion, this regularity is markedly disrupted, with increased cell area and shape irregularity. These alterations are consistent with reduced cortical tension and decreased epithelial stiffness, rendering cells more deformable.

Importantly, in our system, variations in fracturing are explained by changes in tissue stiffness: softening of enterocytes upon myosin II knockout correlates with reduced fracturing area. Thus, in our interpretation, the cell shape index serves as a proxy for epithelial mechanical state (stiffness/deformability), rather than for dynamic tissue flow.

We acknowledge that we did not directly observe cell–cell rearrangements, as this would require long-term intravital or extended *ex vivo* live imaging, which is currently not technically feasible under our experimental conditions.

To avoid confusion, we have revised the manuscript and replaced the term "tissue fluidity" with "tissue deformability" or "tissue softness" throughout the text (see lines 240, 243, and 257 in the revised manuscript).

To nevertheless strengthen our mechanical interpretation beyond the shape index alone, we complemented this analysis with direct rheological measurements using optical tweezers (**Fig. 4f, g**). This approach allows quantification of the viscoelastic properties of individual enterocytes *in situ*. We used a home-built setup combining a fluorescence microscope with an active optical trapping system[9] (see Materials and Methods and Reviewer #1, comment 3 for technical details).

Using this method, we measured cellular rheological properties in control and NMIIA-KO tissues and found that NMIIA-KO enterocytes exhibit more fluid-like mechanical behavior than control cells, in agreement with the predictions from the shape index analysis (**Fig. 4e)**.

Because junctional tension, cytoskeletal contractility, adhesion strength, and cytoplasmic rheology together determine whether a tissue behaves in a more fluid- or solid-like manner[4], we believe that the combination of shape index analysis and optical-tweezers microrheology provides a more robust and quantitative assessment of tissue mechanical state than the shape index alone, even in the absence of direct observation of long-term cell rearrangements.

8. In the model, it is unclear how the distinct properties of enterocytes and goblet cells are incorporated. Are both cell types assumed to have the same mechanical properties? How is their spatial distribution determined or imposed? It is also not evident how these differences are reflected in the evolution equations, for example, in equation (S7). Further clarification on how cell-type-specific features are modeled would be helpful.

Goblet cells and enterocytes don't have the same mechanical properties in simulations: goblet cells have a fixed shape index while enterocyte's shape index varies. Goblet cells are endured with a small shape index ($p_0 = 2$) to ensure the shape roundness. Furthermore, to ensure that the goblet

cell swells isotropically under the compression from surrounding enterocytes, we set both its area rigidity K_A and extensional rigidity K_L at ten times those of the enterocytes.

The simulation box contains 36 enterocytes and 1 (or 2) goblet cell. We randomly select the initial position inside the bulk for single goblet cells (Supplementary Fig. 7B), and for the case with two goblet cells (Supplementary Fig. 7C), these two goblet cells have an interval distance of one (or two) enterocyte cells.

The movement of discrete vertices is driven by the changes in the cellular mechanical energy. In the evolution equation, U_m stands for the mechanical energy of cell m, which can be either enterocyte or goblet cell. The definitions of the mechanical energy of enterocytes and goblet cells are respectively given in Eqs. (S4) and (S6).

For clarification purposes, we have provided more modeling details (especially the cell-type-specific features) on the in the revised “Supplementary Method - Theoretical Modeling”.

Reviewer #4 (Remarks to the Author):

The study presented by Creff et al provides novel and relevant information about how the heterogeneous cell population within the intestinal epithelium contributes to maintenance or breaching of the intestinal barrier function. In fact, the demonstrated that the presence of goblet cells, with their specific cell shape, alters the physical properties and cohesion within the epithelial layer. I believe these findings are sound and important for the scientific community, including biophysics aspects, in order to better understand epithelial barrier function. The methodology, the data, figures and the draft meet the quality requirements for publication. In addition, drawn conclusions are supported by the collected data.

Nevertheless, there are some aspects which are not entirely clear to me, and I think might require revision from the authors.

1. The authors took advantage of succinate to induce goblet cell hyperplasia/hypertrophy, but succinate mediates other effects in the intestinal epithelium, such as the activation of tuft cells and the secretion of IL-25. It would be important to demonstrated that the succinate-mediated effect uniquely depend on the impact on goblet cells, while the effect on other IECs is not relevant.

We thank the reviewer for this important comment. Indeed, succinate acts directly on tuft cells, which are currently the only intestinal epithelial cell type known to express the succinate receptor Sucnr1[14,15]. Upon activation, tuft cells secrete IL-25, which stimulates group 2 innate lymphoid cells (ILC2) to secrete IL-13. IL-13 in turn induces tuft and goblet cell hyperplasia/hypertrophy, leading to the characteristic “weep-and-sweep” response[15,16]. To our knowledge, succinate has not been reported to directly affect other IEC types, and published transcriptomic analyses did not identify changes in gene expression markers for enterocytes, enteroendocrine cells, or Paneth cells following succinate treatment[14].

To further assess whether succinate induces epithelial alterations that could independently influence goblet-associated fracture (GAF) formation, we systematically examined several relevant parameters. We observed no change in enterocyte shape index (**Fig. R12A**), indicating preserved epithelial packing geometry. In addition, junctional protein levels (ZO-1, E-cadherin, and desmocollin) were unchanged (**Fig. R12B–D**), supporting the conclusion that fracture formation does not arise from junctional weakening or loss of adhesion. We also detected no alteration in crypt–villus morphology (**Supplementary Fig. 2g**), indicating that stem cell and Paneth cell compartments remain unaffected.

At the organismal level, succinate-treated mice displayed normal water intake, body weight (**Supplementary Fig. 4b**), intestinal length (**Fig. R12E**), and behavior, and showed no increase in inflammatory cytokines (IL-6, IFN-γ, TNF-α; **Supplementary Fig. 4a**), arguing against systemic inflammation or generalized epithelial stress.

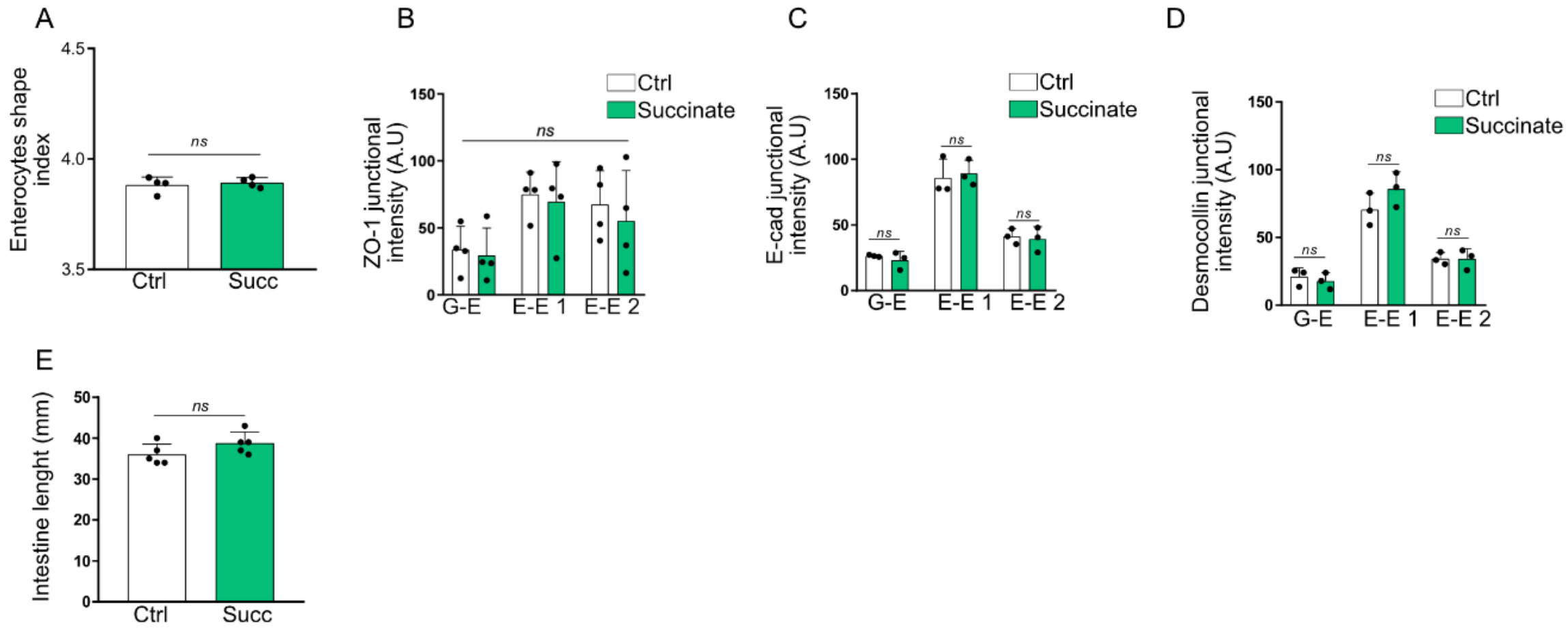

Fig. R12. Succinate treatment does not alter epithelial architecture or junctional integrity. (A) Bar charts showing average enterocyte shape index; t-test ns: non-significant. (B-D) Bar charts displaying average ZO-1 (B), E-cadherin (C) and desmocollin (D) junctional intensity; multiple t-test ns: non-significant. (E) Bar chart showing average intestine length; t-test ns: non-significant.

Taken together, these data indicate that succinate treatment does not trigger a broad epithelial or inflammatory response. Instead, the observed increase in junctional fracturing is specifically associated with goblet cell hypertrophy and the resulting increase in mechanical compression exerted on neighboring enterocytes, rather than indirect effects on other intestinal epithelial cell types.

2. It is also intriguing the fact that the authors used two different strategies to promote the goblet cell hyperplasia in vivo (succinate) and in vitro (DAPT; IWPT2). How comparable are these two situations? actually, succinate did not increase the number of goblet cells, but only the amount of mucus production, while the in vitro conditions affected both number and function of goblet cells.

We thank the reviewer for this insightful comment. We agree that the *in vivo* and *in vitro* approaches used to manipulate goblet cells rely on distinct mechanisms and are therefore not directly equivalent, and we have clarified this point.

Succinate treatment, used to induce goblet cell (GC) hypertrophy *in vivo*, acts indirectly through activation of the ILC2–IL-13 axis, leading to increased mucus production and enlargement of existing goblet cells. Importantly, under our experimental conditions, succinate did not increase goblet cell number, but rather enhanced intracellular mucin granules volume.

In contrast, treatment with DAPT (a Notch pathway inhibitor) and IWP2 (a Wnt pathway inhibitor) *in vitro* acts directly on epithelial cells, altering cell fate decisions and inducing both goblet cell hyperplasia and hypertrophy, thereby affecting goblet cell number as well as function.

The use of these two distinct strategies was motivated by experimental constraints and complementarity. Succinate cannot be used in 2D organoid systems, which lack immune cells and therefore the ILC2–IL-13 signaling axis required for succinate-mediated effects. Conversely, *in*

vivo manipulation of the Notch pathway would result in profound and systemic alterations of epithelial renewal and cell fate: Notch inhibition (genetic or pharmacological) converts proliferative progenitors into goblet cells at the expense of epithelial proliferation, impairing nutrient absorption and leading to secretory diarrhea, dehydration, weight loss and often death within days[17], whereas constitutive Notch activation eliminates goblet cells leading to crypt hyperplasia and altered villus architecture[18].

To minimize confounding effects from goblet cell hyperplasia *in vitro* and focus specifically on the mechanical consequences of goblet cell hypertrophy, we quantified goblet-associated fractures only around goblet cells that were surrounded by several rows of enterocytes. Thus, while our two approaches (*in vivo* vs *in vitro* organoids) are mechanistically distinct and not directly comparable, each provides a controlled context to probe how increased goblet cell size via increased mucin granule load mechanically impact epithelial integrity.

3. Another important aspect is the description of biological/technical replicates. In most figure legends, it is mentioned n = x number of experiments, but there is no explanation about the biological replicate number within each experiment. In contrast, in the methods section, it is mentioned that the n describes the number of mice (in vivo) or the number if experiments (in vitro). I think the information is not entirely clear, and it is therefore hard to judge the signifcance of the presented data, in some cases.

We apologize for not clearly explaining biological and technical replicates. In all figures, "n" refers to the number of biological replicates, defined as individual mice for *in vivo* experiments and independent culture preparations for *in vitro* experiments. For each biological replicate and condition, at least three independent technical replicates were performed. We have now clarified this information in the Method section.

Minor comments:

1. it would be helpful revising the order of the figures and adapt them to their appearance in the text. Sometimes, the reader has to go back and forth through the figures, because they do not appear in order.

We have reorganized the figures, making the manuscript easier to follow.

2. ED Fig. 1: it contains twice the subfigure C.

We apologize for this oversight and have corrected the figure accordingly.

3. ED Fig. 8a: pictures not really representative of the quantification in ED Fig. 8B

We apologize for any confusion caused by the still images shown in previous Extended Data Fig. 8a. In these images, junctional recoil is difficult to appreciate because it is partially obscured by the junctional bleach associated with laser ablation and because the recoil amplitude is relatively

small. To more clearly convey the recoil dynamics quantified in Extended Data Fig. 8b, we now provide representative time-lapse movies that better capture junctional recoil following ablation (**Supplementary Movies 4–6**).

4. ED Fig. 1B: why the mucus is going beyond the GC?

We understand the reviewer's confusion. In Supplementary Fig. 1b, optical planes slightly above the goblet cell body, within the apical region, were projected to capture tight junctions of goblet cells and their neighbours. As a result, mucus that has been released apically into the lumen in this particular example is included in the projection and can appear to extend beyond the goblet cell outline in the x–y view. Orthogonal projections (**Fig. R13**) confirm that this mucus signal is located above the goblet cell, within the lumen, and not within the lateral epithelial compartment.

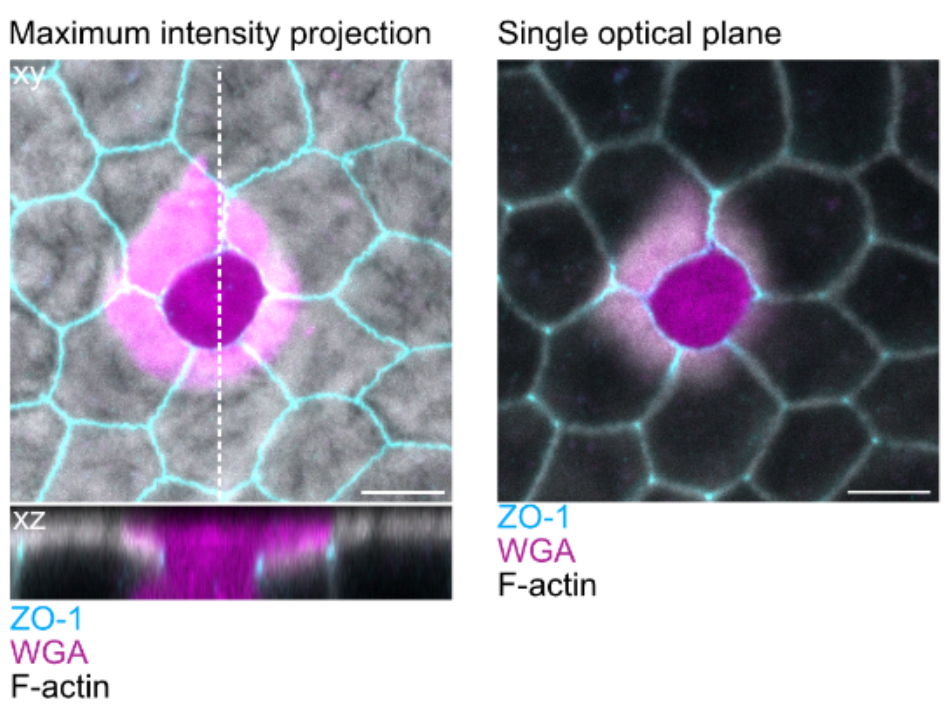

Fig. R13. Left: Representative orthogonal (xy and xz) confocal images of murine intestinal tissue; maximum intensity projection (Z range, 3.2 µm), orthogonal projection. Right: *en face* single optical section of murine intestinal tissue. Scale bars, 5 µm.

References

1. Nikitas, G. *et al.* Transcytosis of Listeria monocytogenes across the intestinal barrier upon specific targeting of goblet cell accessible E-cadherin. *Journal of Experimental Medicine* **208**, 2263–2277 (2011).
2. Tsukita, S., Furuse, M. & Itoh, M. Multifunctional strands in tight junctions. *Nat Rev Mol Cell Biol* **2**, 285–293 (2001).
3. Reth, M. Matching cellular dimensions with molecular sizes. *Nat Immunol* **14**, 765–767 (2013).
4. Hannezo, E. & Heisenberg, C.-P. Rigidity transitions in development and disease. *Trends Cell Biol* **32**, 433–444 (2022).
5. Atia, L., Fredberg, J. J., Gov, N. S. & Pegoraro, A. F. Are cell jamming and unjamming essential in tissue development? *Cells & Development* **168**, 203727 (2021).
6. Mao, Y. & Wickström, S. A. Mechanical state transitions in the regulation of tissue form and function. *Nat Rev Mol Cell Biol* **25**, 654–670 (2024).
7. Park, J.-A. *et al.* Unjamming and cell shape in the asthmatic airway epithelium. *Nature Mater* **14**, 1040–1048 (2015).

8. Bocanegra-Moreno, L., Singh, A., Hannezo, E., Zagorski, M. & Kicheva, A. Cell cycle dynamics control fluidity of the developing mouse neuroepithelium. *Nat. Phys.* **19**, 1050–1058 (2023).
9. Merle, T. *et al.* Deep-tissue absolute force spectroscopy with sub-piconewton precision. 2026.03.23.712846 Preprint at https://doi.org/10.64898/2026.03.23.712846 (2026).
10. Hendricks, A. G., Holzbaur, E. L. F. & Goldman, Y. E. Force measurements on cargoes in living cells reveal collective dynamics of microtubule motors. *Proc Natl Acad Sci U S A* **109**, 18447–18452 (2012).
11. Bas, J., Jay, P. & Gerbe, F. Intestinal tuft cells: Sentinels, what else? *Seminars in Cell & Developmental Biology* **150–151**, 35–42 (2023).
12. Cheng, C. K., Wang, N., Wang, L. & Huang, Y. Biophysical and Biochemical Roles of Shear Stress on Endothelium: A Revisit and New Insights. *Circulation Research* **136**, 752–772 (2025).
13. Lecuit, T. & Yap, A. S. E-cadherin junctions as active mechanical integrators in tissue dynamics. *Nat Cell Biol* **17**, 533–539 (2015).
14. Lei, W. *et al.* Activation of intestinal tuft cell-expressed Sucnr1 triggers type 2 immunity in the mouse small intestine. *Proceedings of the National Academy of Sciences* **115**, 5552–5557 (2018).
15. Nadjsombati, M. S. *et al.* Detection of Succinate by Intestinal Tuft Cells Triggers a Type 2 Innate Immune Circuit. *Immunity* **49**, 33-41.e7 (2018).
16. Schneider, C. *et al.* A Metabolite-Triggered Tuft Cell-ILC2 Circuit Drives Small Intestinal Remodeling. *Cell* **174**, 271-284.e14 (2018).
17. van Es, J. H. *et al.* Notch/gamma-secretase inhibition turns proliferative cells in intestinal crypts and adenomas into goblet cells. *Nature* **435**, 959–963 (2005).
18. Fre, S. *et al.* Notch signals control the fate of immature progenitor cells in the intestine. *Nature* **435**, 964–968 (2005).

Response to Reviewers

Reviewer #3

We thank the reviewer for their positive assessment of the revised manuscript and for recommending publication.

Comment:
I would like to thank the authors for the revised version of the manuscript. They have clearly addressed my questions, and I can recommend publication of the article.

I have only one final comment, but I do not need to re-review the paper. It should be made clear that the rheological measurements with optical tweezers were performed intracellularly. Indeed, probing cell rheology from the outside or from the inside can lead to different, and sometimes unexpected, results. For example, disruption of the actin cytoskeleton does not necessarily appear to affect intracellular mechanical properties, as reported by Muenker et al. in eLife 2024.

I would therefore be slightly more cautious with conclusions stating that the cells have lower compressive resistance, since this interpretation is more directly related to cortical activity than to intracellular mechanical properties. However, it would make sense that NMIIA-KO cells may also exhibit reduced cortical stiffness. This distinction may also explain why the authors obtain relatively low values for the storage modulus.

Response:
We thank the reviewer for this important comment. We agree that it is essential to distinguish between intracellular and cortical mechanical properties when interpreting rheological measurements. To clarify this point, we have revised the manuscript to explicitly state that the optical tweezers measurements were performed intracellularly, by tracking endogenous lipid droplets within the cytoplasm (line 252).

We also agree that intracellular microrheology does not directly probe cortical mechanics, and that perturbations of the actin cytoskeleton may differentially affect intracellular and cortical mechanical properties. In light of this, we have revised the text to emphasize that the measured storage and loss moduli reflect cytoplasmic viscoelasticity, rather than direct measurements of cortical stiffness (lines 250, 254 and 258).

These modifications clarify our mechanical measurements and avoid overinterpreting intracellular rheological measurements as direct readouts of cortical stiffness.
