# Supplementary material for: Goblet cells mechanically breach the epithelial barrier in gut homeostasis
Source: Nat Commun. 2026 Jul 25;17:9078. doi: 10.1038/s41467-026-76034-0 (PMC13518819; doi:10.1038/s41467-026-76034-0)
Supplement: Supplementary file 2 [file 41467_2026_76034_MOESM1_ESM.pdf]

Supplementary information

Goblet cells mechanically breach the epithelial barrier in gut homeostasis

Justine Creff[1], Yuan He[2], Sandra Bernat-Fabre[1#], Etienne Buscail[3#], Salomé Neuvendel[1#], Vishnu Krishnakumar[1], Dhriti Saumya[1], Laurent Malaquin[4], Thomas Mangeat[5], Shi-Lei Xue[2*], Denis Krndija[1*]

[1]Centre de Biologie Intégrative (CBI), University of Toulouse, CNRS, Toulouse, France

[2]Department of Materials Science and Engineering, School of Engineering, Westlake University, Hangzhou, Zhejiang, PR China

[3]Department of Digestive Surgery, Colorectal Surgery Unit, Toulouse University Hospital, Toulouse, France

[4]LAAS-CNRS, University of Toulouse, CNRS, Toulouse, France

[5]LITC Core Facility, Centre de Biologie Intégrative (CBI), University of Toulouse, CNRS, Toulouse, France

[#]These authors contributed equally to this work.

[*]Corresponding authors: xueshilei@westlake.edu.cn; denis.krndija@utoulouse.fr

The file includes:

Supplementary Figures 1 to 8
Supplementary Method - Theoretical Modeling

Supplementary Figures

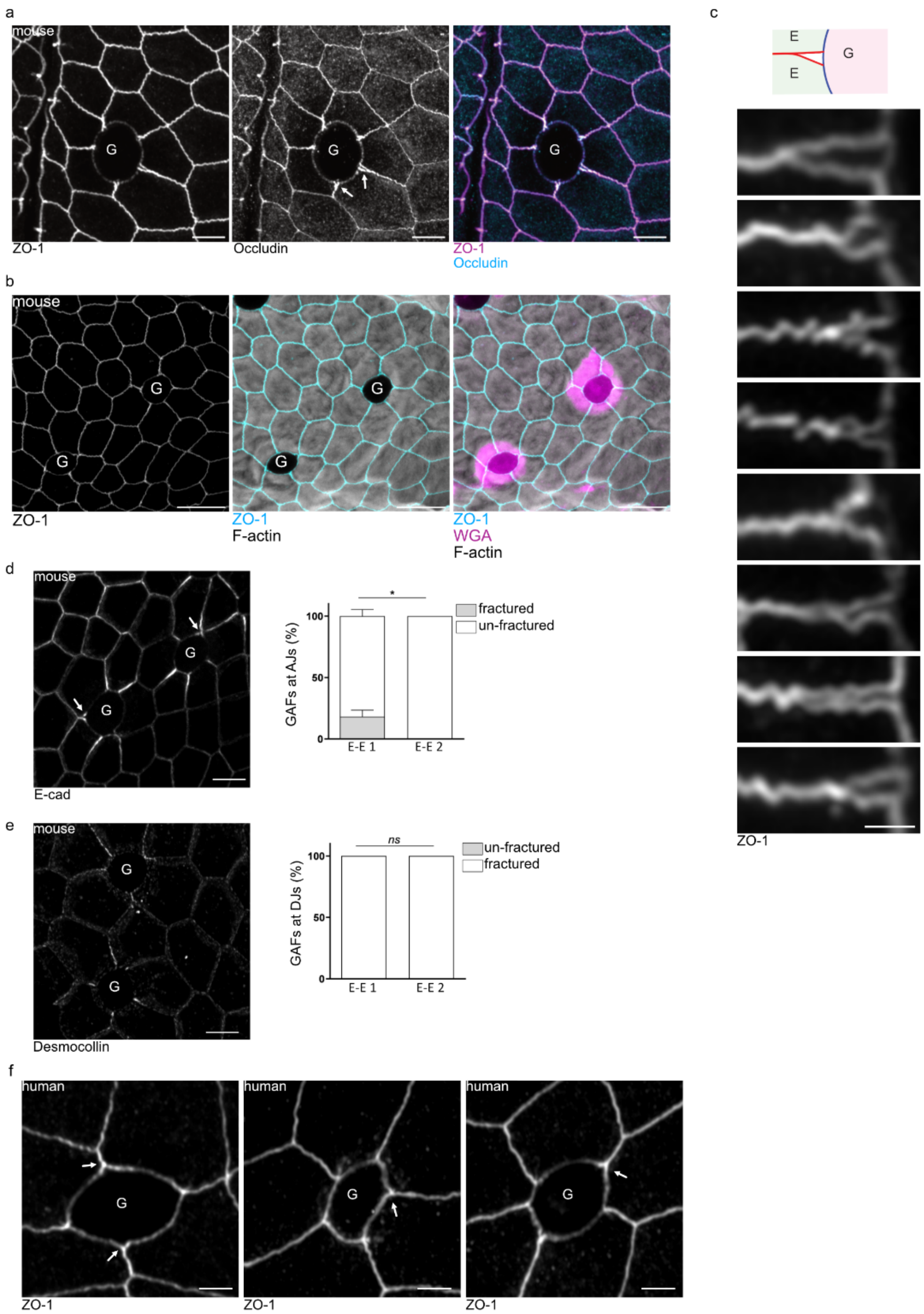

Supplementary Fig. 1. a Representative *en face* images of murine tissue; maximum intensity projection (Z range, 3.5 µm). White arrows are showing fractures in 1st neighbor E-E junctions, Scale bars, 5 µm. Similar results were observed in at least three independent biological replicates. **b** Representative *en face* images of murine intestinal tissue; maximum intensity projection (Z range, 3.2 µm). Scale bars, 10 µm, n=3 biological replicates Similar results were observed in at least three independent biological replicates **c** Montage of high-resolution images showing fractures at the goblet-enterocyte-enterocyte tricellular junction. Scale bar, 2 µm. **d** Representative

en face image of murine intestinal tissue; MIP (Z range, 0.9µm) (right), stacked bar graph showing average percentage of enterocytes+/- SD associated with fractures at AJs (left); Two tailed Multiple t-test with FDR correction, *: p = 0,01026, n=3 biological replicates. **e** Representative *en face* image of murine intestinal tissue; maximum intensity projection (Z range, 1.8µm) (right), stacked bar graph showing average percentage +/- SD of enterocytes associated with fractures at desmosome level (left) Multiple t-test, ns: non-significant; p > 0,9999, n=3 biological replicates. **f** Representative *en face* images of human ileum; maximum intensity projection (Z range, 1.4 – 1.8 µm). White arrows are showing fractures in 1st neighbor E-E junctions. Scale bars, 2 µm.

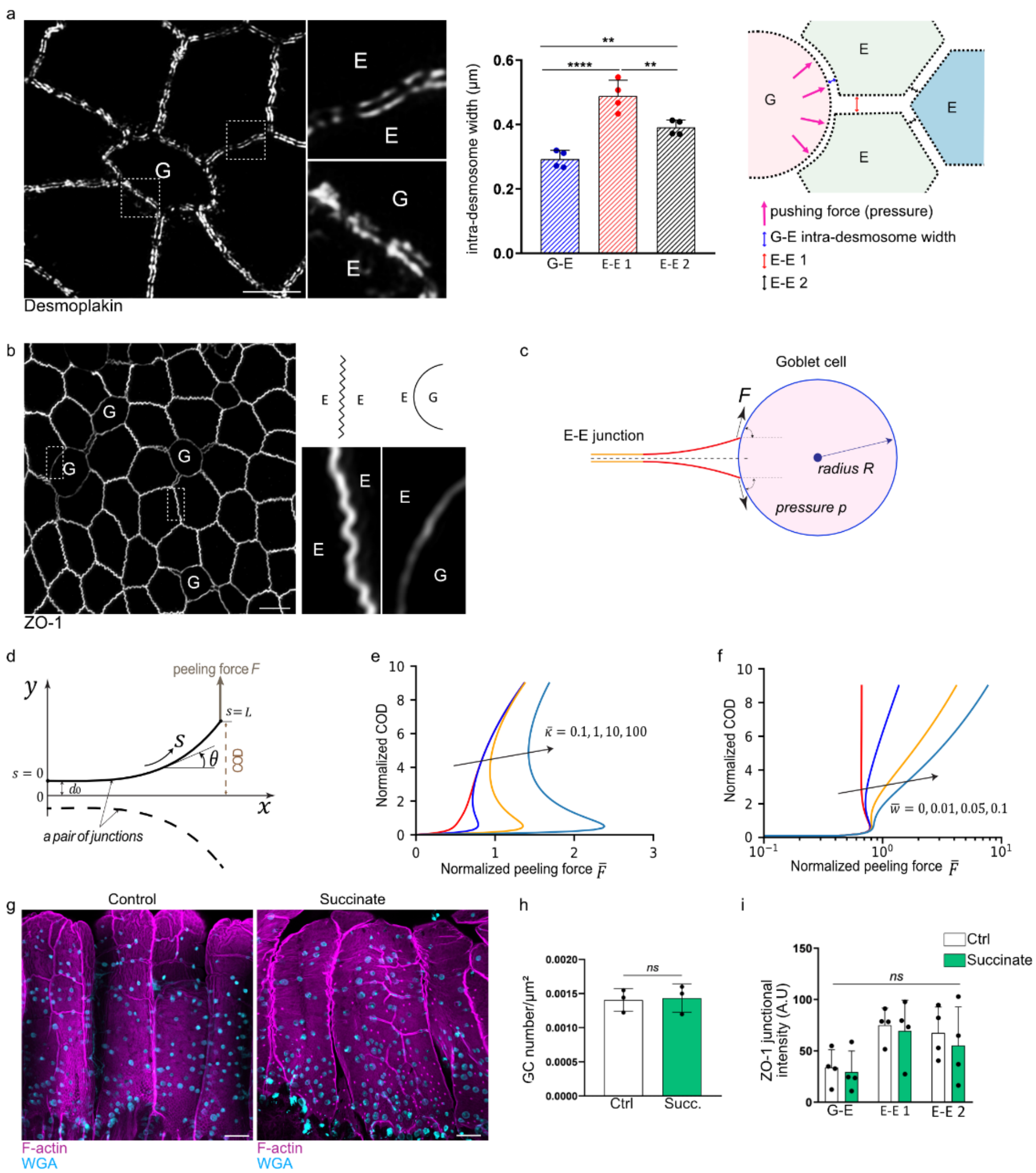

Supplementary Fig. 2. a Left: super-resolution (RIM) *en face* images of intestinal tissue; boxed regions are shown in higher magnification; maximum intensity projection (Z range, 1.2 µm). Middle: Bar chart displaying average intra-desmosomal width +/- SD. Right: schematic representation displaying intra-desmosomal width analysis. Two-tailed Multiple t-test with FDR correction, **: p =0,0083 , ****: p < 0.0001; n=4 biological replicates. Scale bar, 2 µm. **b** Representative images showing E-E and G-E TJs; boxed regions are shown in higher magnification; maximum intensity projection (Z range, 3.1 µm). Scale bar, 5 µm. **c** Schematic to show the fracture mechanics of an E-E junction driven by forces exerted by the GC. **d** Schematic and parameter definitions of the crack mechanics model of cell-cell interface (s denotes the arclength along the deformed cell boundary (i.e., the elastic beam), and θ is the angle between the initial boundary profile). **e-f** Sensitivity analysis of mechanical parameters to understand how junctional cracks develop due to the peeling force arising from the swelling of goblet cells (see also panel C). The crack growth is characterized as the normalized "crack opening displacement" (COD). Specifically, panels E and F analyze the influence of **e** the effective bending rigidity of the cell boundary ($\bar{\kappa}$, with $\bar{w} = 0.01$) and **f** the bridging strength of external components ($\bar{w}$, with $\bar{\kappa} =$

1), respectively. **g** Representative low magnification images showing whole villi of control and succinate treated tissue stained; maximum intensity projection (Z range, 13.95 µm and 11.2 µm). Scale bars, 50 µm. **h** Bar chart displaying average GC number per μm^2. Mann-Whitney test, ns: non-significant; p >0,9999; n=3 biological replicates. **i** Bar charts showing average ZO-1 junctional intensity. Two-tailed Multiple t-test with FDR correction, ns: non-significant; p = 0,9996; n=4 biological replicates. Ctrl, control; Succ., succinate.

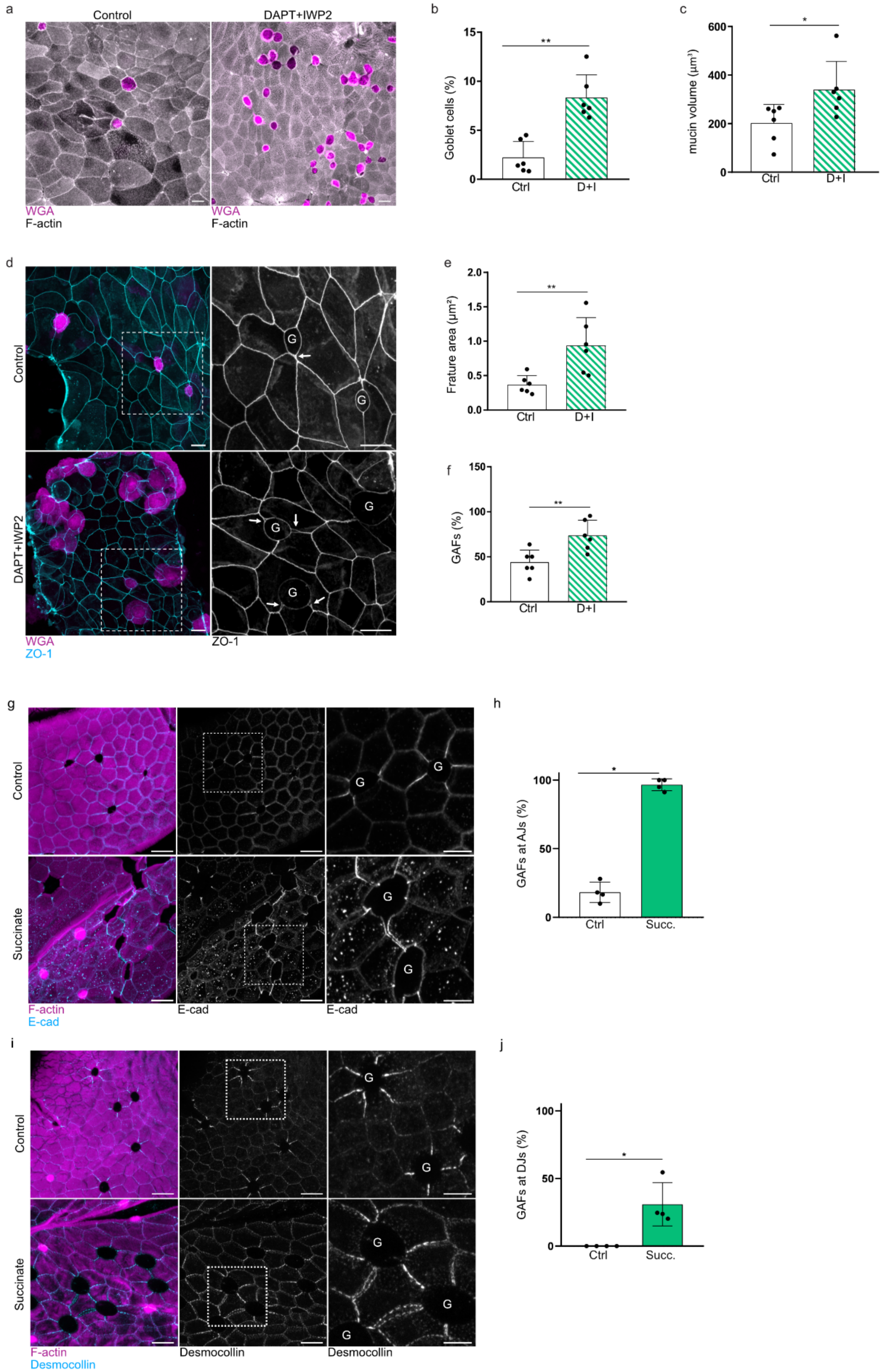
a
Control
DAPT+IWP2
WGA
F-actin
WGA
F-actin
b
Goblet cells (%)
15
10
5
0
**
Ctrl
D+I
c
mucin volume (µm³)
600
400
200
0
*
Ctrl
D+I
d
Control
DAPT+IWP2
G
WGA
ZO-1
ZO-1
e
Frature area (µm²)
2.0
1.5
1.0
0.5
0.0
**
Ctrl
D+I
f
GAFs (%)
150
100
50
0
**
Ctrl
D+I
g
Control
Succinate
G
F-actin
E-cad
E-cad
E-cad
h
GAFs at AJs (%)
100
50
0
*
Ctrl
Succ.
i
Control
Succinate
G
F-actin
Desmocollin
Desmocollin
Desmocollin
j
GAFs at DJs (%)
100
50
0
*
Ctrl
Succ.

Supplementary Fig. 3. a Representative images of 2D organoids treated or not with DAPT+IWP2, showing GC hyperplasia; maximum intensity projection (Z range, 3.3 µm and 8.4 µm). Scale bars, 10 µm. Similar results were observed in at least three independent biological replicates. **b** Bar chart showing average percentage +/- SD of goblet cell in 2D organoids according the conditions. Two-sided Mann-Whitney test, **: p = 0.0022; n=6 biological replicates. **c** Bar chart displaying average intracellular mucin volume +/- SD. Two-tailed Mann-Whitney test, *: p= 0,0152 ; n=6 experiments. **d** Representative images of control and DAPT+IWP2-treated 2D organoids. Boxed regions are shown in higher magnification. White arrows are showing GAFs. Maximum intensity projection (Z range, 3.3 µm and 8.5 µm). Scale bars, 10 µm. **e-f** Bar charts showing average fracture area +/- SD Two-tailed Mann-Whitney test, **: p = 0,0087; n=6 biological replicates **e** and average percentage of GAFs +/- SD at TJ level Two-tailed Mann-Whitney test, **: p = 0,0065; n=6 biological replicates **f**. Ctrl, Control; D, DAPT; I, IWP2. **g** Representative images from control and succinate-treated mice; maximum intensity projection (Z range, 1.8 µm, 2.1 µm); scale bars, 10 µm. Boxed regions are shown in higher magnification. Scale bars, 5µm. **h** Bar chart displaying average percentage +/- SD of GAFs at AJs level. Two-tailed Mann-Whitney test *: p= 0,0286 ; n=4 biological replicates. **i** Representative images from control and succinate-treated mice.; maximum intensity projection (Z range, 4.2 µm and 3.3 µm); scale bars, 10 µm. Boxed regions are shown in higher magnification; scale bars, 5 µm. **j** Bar chart displaying average percentage of GAFs +/- SD at desmosome level. Two-tailed Mann-Whitney test, *: p = 0,0286 ; n=4 biological replicates. Ctrl, control; Succ., succinate.

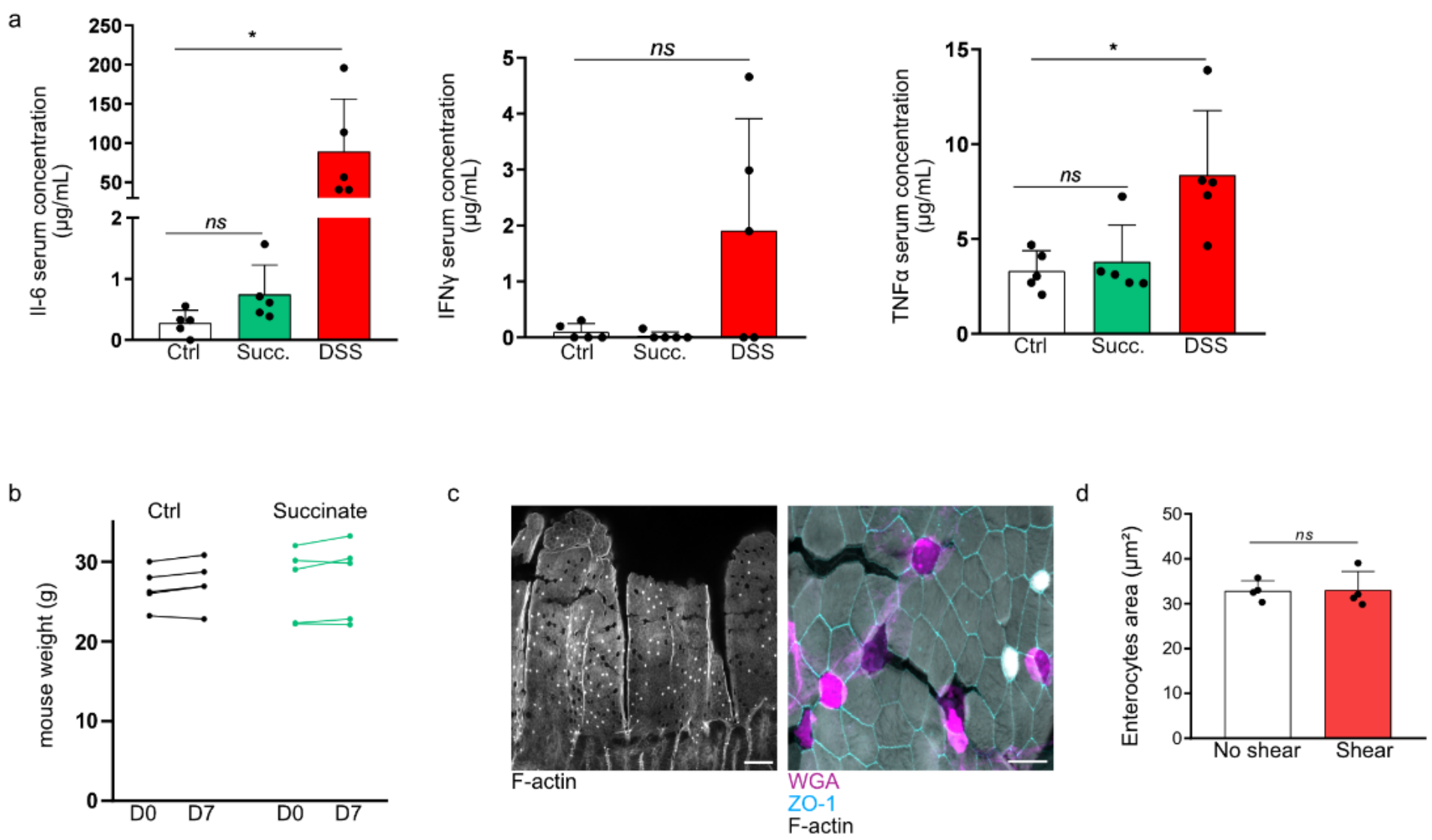

Supplementary Fig. 4. a Bar charts showing IL-6, IFNγ and TNF-α average serum concentration +/- SD. Two-tailed Multiple t-test with FDR correction: ns: non-significant, p = 0.9428; *: p 0.0159 ; n=5 biological replicates. **b** Control and succinate mice weight plot. Multiple t-test; n=5 biological replicates. **c** Representative images of tissue from succinate-treated mice, treated with luminal shear stress *ex vivo.* Left: low magnification image, showing damage at the tip of the villi. Scale bar, 50 µm. Right: High-resolution image showing long-range fracturing emanating from GCs. Maximum intensity projection (Z range, 6.6 µm) Similar results were observed in at least three independent biological replicates. **d** Bar chart showing the average enterocytes area +/-SD. Two-tailed Mann-Whitney test, ns: non-significant, p = 0.9496. n=4 biological replicates. Scale bar, 10 µm. Ctrl, Control; Succ., succinate.

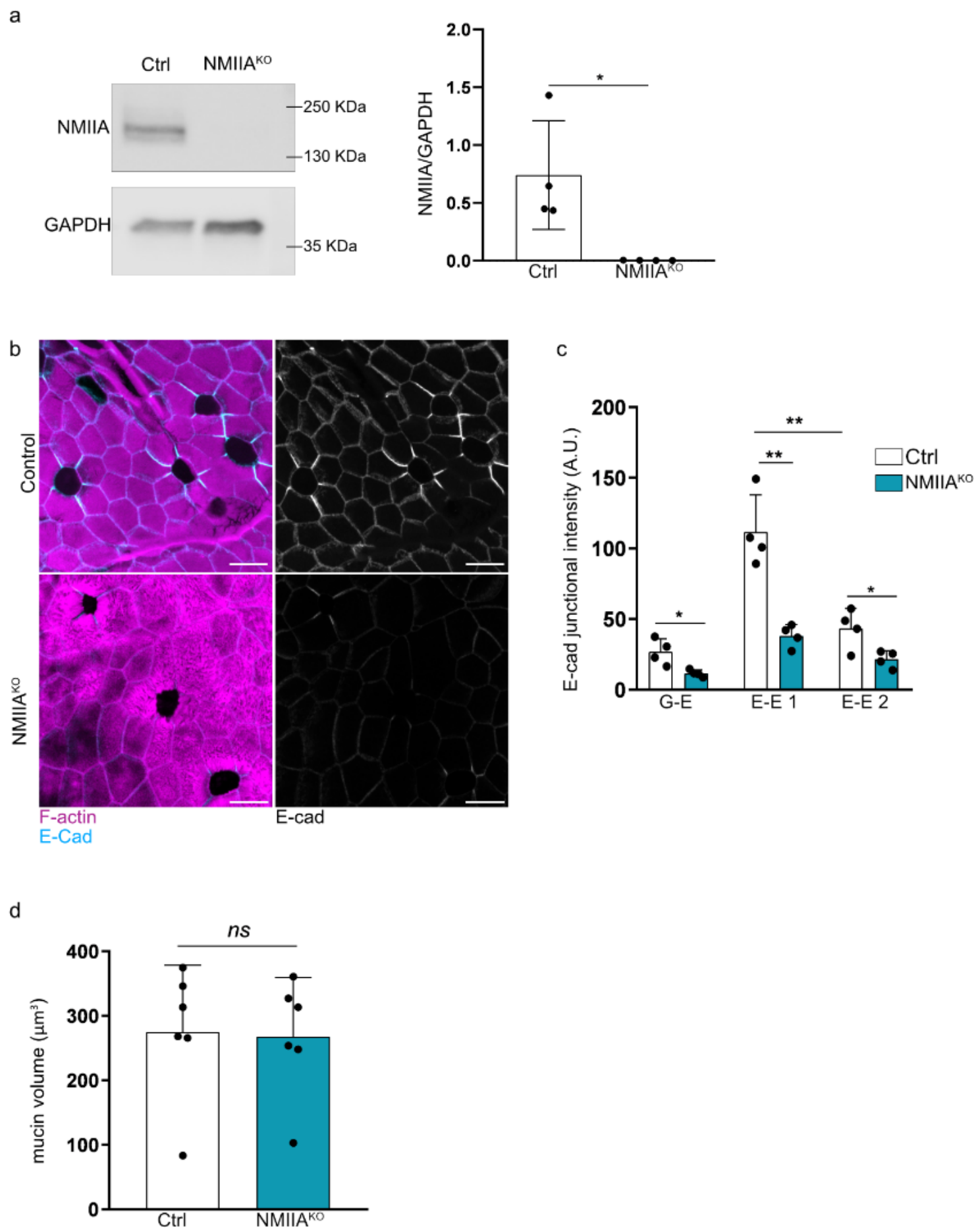

Supplementary Fig. 5. **a** Left: Representative western blot for NMIIA from control and NMIIA^{KO} small intestinal epithelium; GAPDH was used as loading control. Right: Bar chart showing NMIIA signal intensity normalized to GAPDH. Two-tailed Mann-Whitney test, *: p= 0.0286; n= 4 biological replicates. **b** Representative *en face* images of control and NMIIA^{KO} tissue; maximum intensity projection (Z range, 2.4 µm and 1.8 µm). Scale bars, 10 µm. **c** Bar chart showing average E-cadherin junctional intensity +/- SD in control or NMIIA^{KO} tissue. Two-tailed Multiple t-test, *: p= 0.0340, **: p= 0.0017; n=4 biological replicates. **d** Bar graph displaying average intracellular mucin volume +/- SD. Two-sided Mann-Whitney test, ns=non-significant, p= 0.8182; n=5 biological replicates.

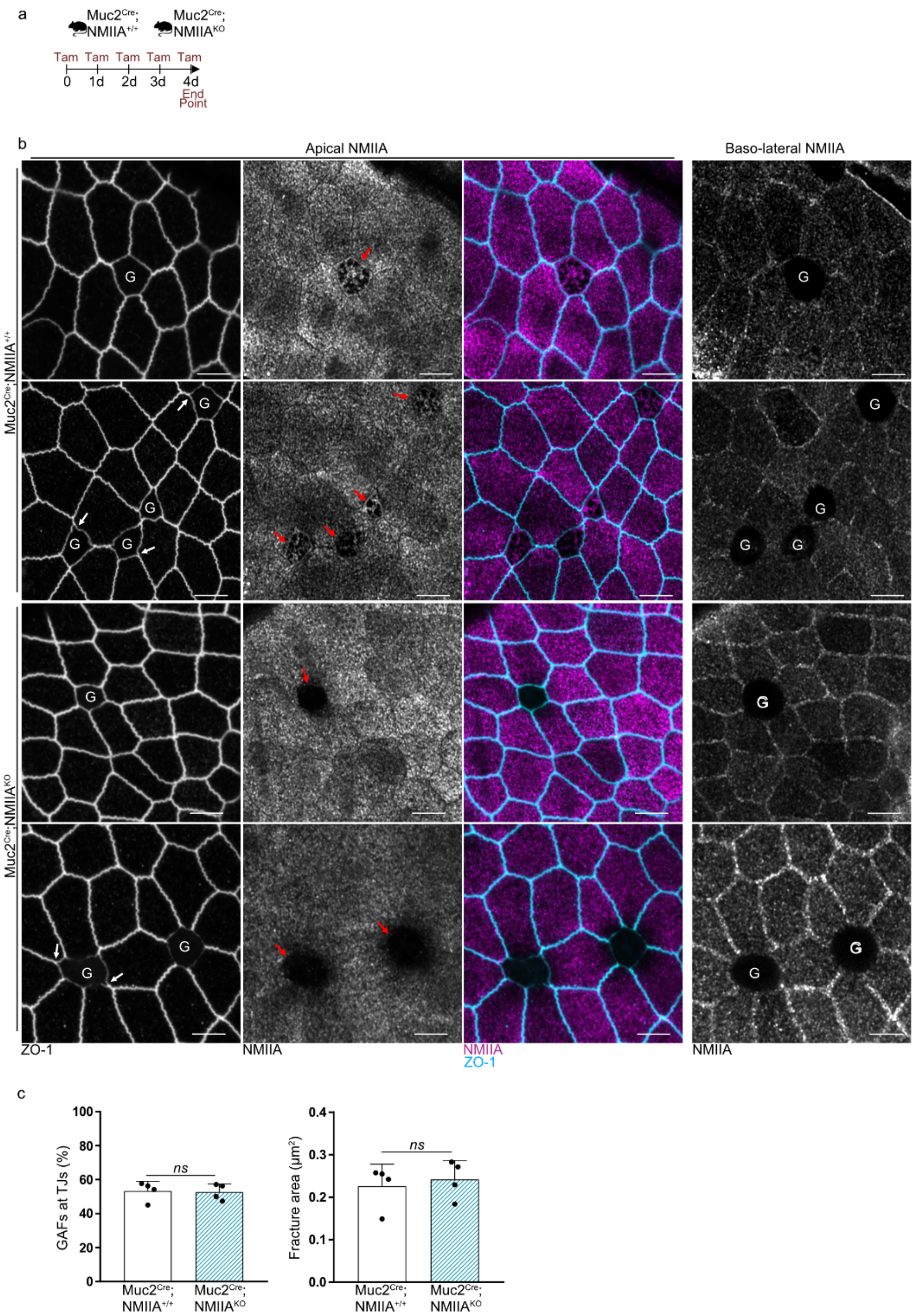

Supplementary Fig. 6. a Schematic representation of experimental procedure for Muc2$^{CRE\text{-}ERT2}$;NMIIA^{KO}. **b** Representative high resolution *en face* images from control and Muc2$^{CRE\text{-}ERT2}$;NMIIA^{KO} mice. White arrows show GAFs and red arrows show NMIIA ring in goblet cells. Scale bars, 5 µm. Similar results were observed in at least three independent biological replicates. **c** Left: Bar charts displaying the average percentage of GAFs +/- SD at TJs. Two-tailed Student t-test, ns: non-significant, p= 0.8797; n=4 biological replicates. Right: Bar graph showing average fracture area +/- SD. Two-tailed Student t-test, ns: non-significant, p= 0.6588; n=4 biological replicates.

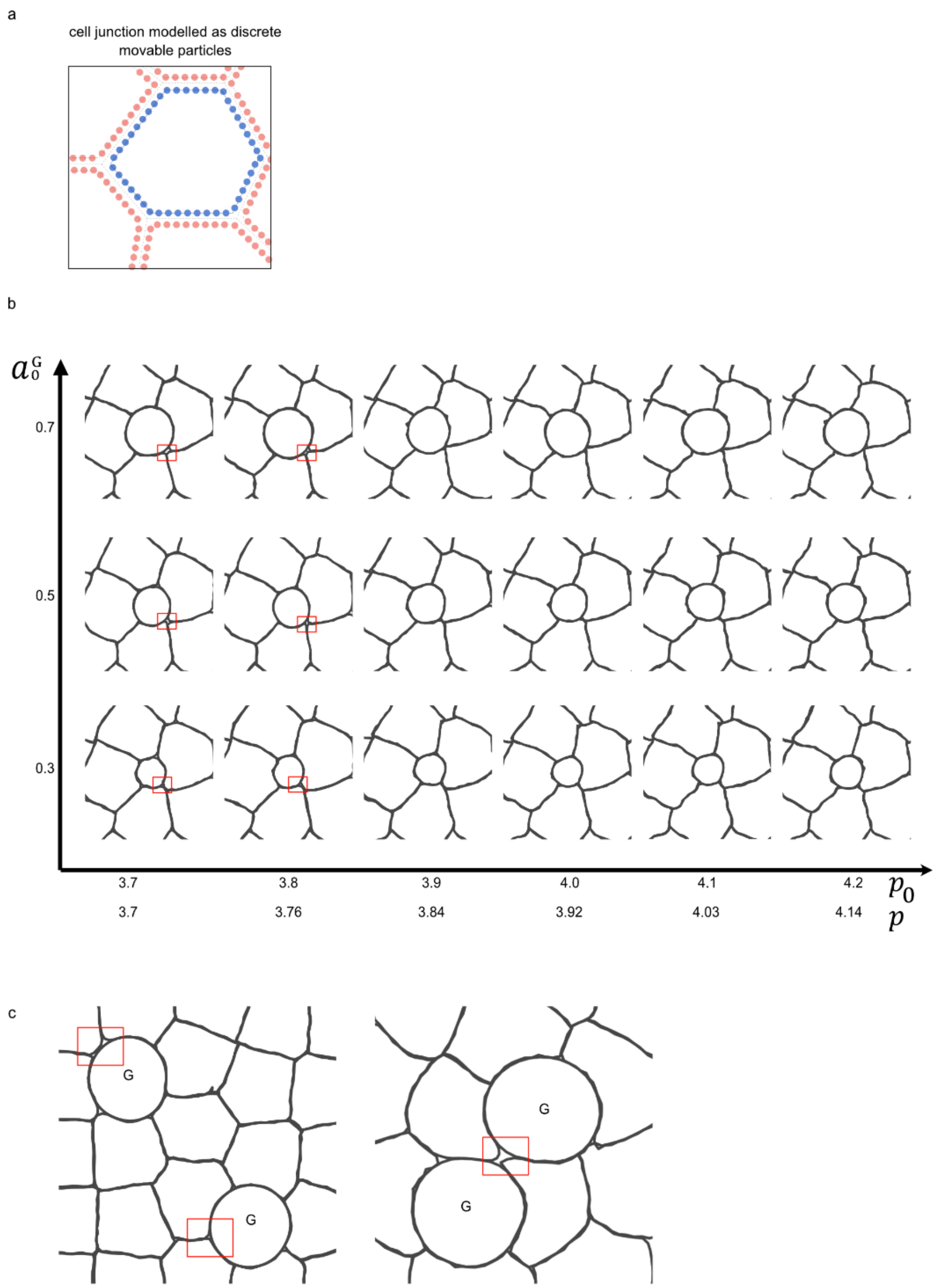

Supplementary Fig. 7. a Cartoon representation of the tissue mechanics model, which treats the cell boundaries as discrete movable particles to track the cellular shape change and crack formation at cell-cell junctions. **b** Simulations of the intestinal epithelial junctions based on the tissue mechanics model (see Supplementary Method - Theoretical Modeling for details). The two key parameters are the preferred area (a_0^G, see *y*-axis) of the goblet cell (which has otherwise identical properties compared to the other surrounding cells), and the preferred shape index of all cells in

the tissue (p_0, see x-axis). **c** Model simulation showing cumulative positional effect of GCs on fracture area. Goblet cells shown are either separated by 2 rows (left) of 1 row of enterocytes (right); a_0^{G} = 0.7, p_0= 3.8.

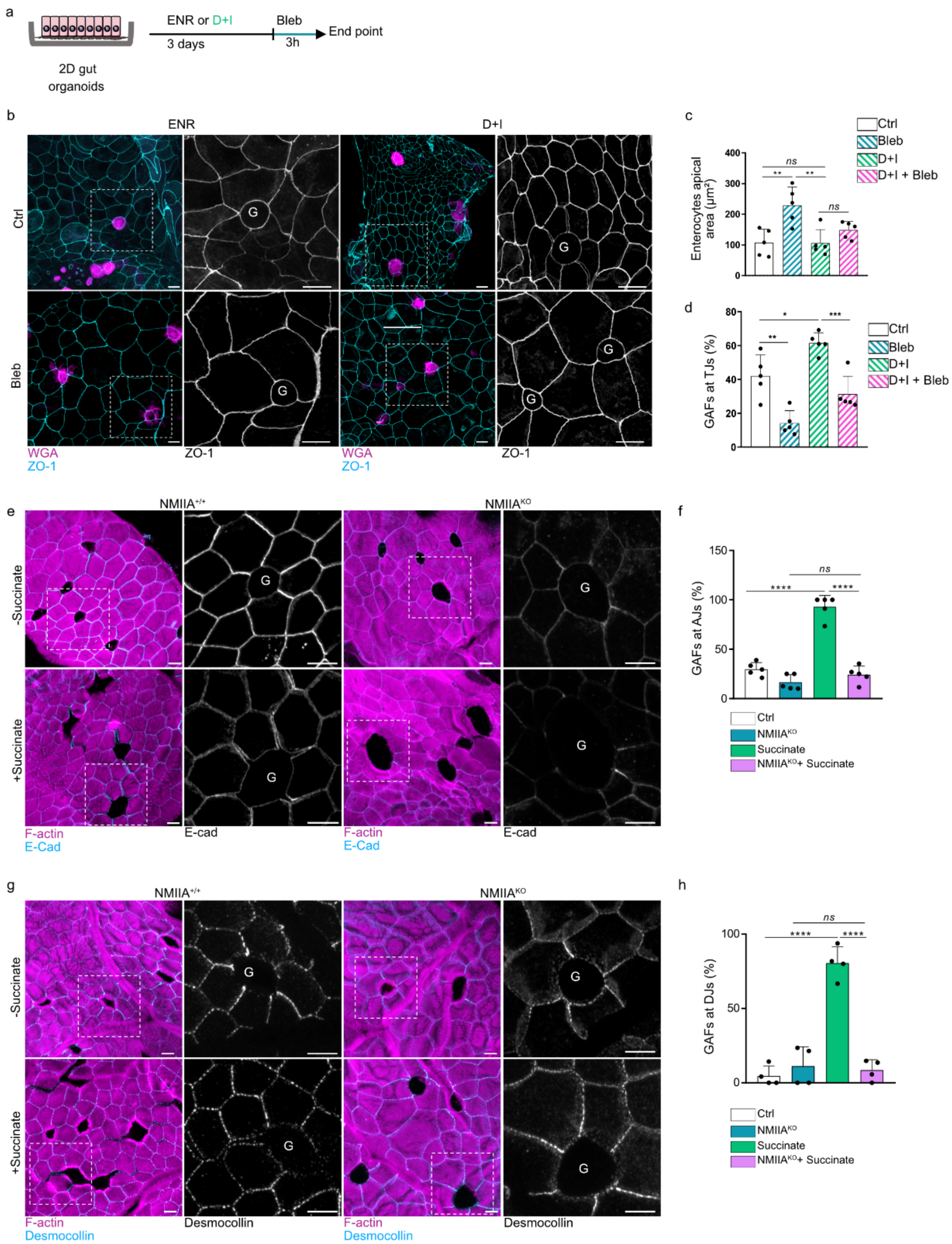

Supplementary Fig. 8. **a** Schematic representation of blebbistatin treatment in 2D organoids pre-treated with DAPT (D) and IWP2 (I). **b** Representative images of 2D organoids treated with blebbistatin (Bleb) or vehicle control (DMSO). Scale bars, 10 µm. **c** Bar chart displaying enterocytes' average apical cell area +/- SD according to the culture condition. Two-tailed Multiple t-test with FDR correction, ns: non-significant, p = 0,4635; **: p = 0,0026. n = 5 biological replicates. **d** Bar graph showing the percentage of GAFs +/- SD at the TJ level. Ctrl, vehicle control (DMSO); Two tailed Multiple t-test with FDR correction, *: p = 0.0218, **: p = 0. 0012; ***: p =

0.0006. n = 5 biological replicates. D, DAPT; I, IWP2. **e** Representative images of tissues from NMIIA$^{+/+}$ and NMIIA^{KO} mice with and without succinate treatment; maximum intensity projection (Z range, 2.4 – 4.2 µm). Boxed regions are shown in higher magnification. Scale bars, 10 µm. **f** Bar chart displaying average percentage +/- SD of GAFs at AJ level. Two-tailed Multiple t-test with FDR correction, ****: $p < 0.0001$; n=5 biological replicates. **g** Representative images from NMIIA$^{+/+}$ and NMIIA^{KO} mice with and without succinate treatment; maximum intensity projection (Z range, 1.4 – 2.8 µm). Boxed regions are shown in higher magnification. Scale bars, 10 µm. **h** Bar chart showing average percentage +/- SD of GAFs at the desmosome level. Two-tailed Multiple t-test with FDR correction, ****: $p < 0.0001$; n=4 biological replicates.

Supplementary Method - Theoretical Modeling

In this Theoretical Modeling chapter, we provide details on the fracture mechanics of intestinal epithelia. In an intestinal epithelium, goblet cells swell and push the adjacent enterocytes. Such mechanical challenge strains the junctions between enterocytes that surround the goblet cell and cause small cracks. We first assume the crack formation can be completely determined by the mechanical interaction between the goblet cell and a single junction between neighboring enterocytes, and evaluate the detachment of cell-cell junction (Section 1). This model can well explain the fact that the swelling of goblet cells promotes crack formation, but fails to explain why a tissue with more compliant cells shows less cracks. Such inconsistency indicates that the crack formation might not solely be a result of localized mechanical interaction, but also be dependent on the mechanical property of the whole tissue. We then consider a multiscale tissue model that involves the swelling of goblet cells and formation of cracks at cell-cell junctions as a function of surrounding tissue rheology (Section 2).

1. Crack mechanics of cell-cell interface

A cell-cell interface mainly contains intercellular junctions, plasma membrane and cell cortices – thin layers of actomyosin meshwork beneath the plasma membrane. Cell–cell junctions are composed of transmembrane proteins that mediate adhesion between adjacent cell surfaces, which include both the plasma membrane and the underlying cortex. These surfaces form an elastic structure capable of withstanding mechanical forces from the environment (e.g., swollen goblet cells). As mucin granules accumulate, the internal pressure within goblet cells may increase and exceed that of neighboring enterocytes. This pressure imbalance generates a tensile force, F, along the goblet–enterocyte interface (see **Supplementary Fig. 3c** for schematic). According to the Young–Laplace equation, this force F is positively correlated with both the pressure difference and the size of the goblet cell.

Previous studies indicate that the tensile force F could act as a "peeling force" and cause interface debonding[1–3]. To check if goblet cells induce junctional cracks in a similar way, here we propose a mechanical model that considers the deformation and debonding of cell-cell interface. Given that most junctional fractures happen to tight junctions, which are located near the apical surface of the epithelium, it is reasonable to assume that the deformation and debonding of the cell-cell interface mainly happen in a two-dimensional plane (i.e., the tissue surface). At the tissue surface, the cell-cell interface can be depicted as a pair of cell boundaries that is initially linked with tight junctions and withstands peeling force F at one end. Given the mechanical properties of relevant cellular components, the cell-cell interface can be modelled as two initially parallel elastic beams (i.e., the cell boundaries) with bridging forces from tight junctions (**Supplementary Fig. 3d**). The cellular components aside from the cell interface (e.g., the apical or lateral surface) also hinder the formation and extension of junctional cracks, thus also contribute to the bridging forces in our model.

We introduce curvilinear coordinates (s, θ), where s denotes the arclength along the deformed cell boundary (i.e., the elastic beam), and θ is the angle between the initial boundary profile (parallel to x-axis) and the tangent to its current profile (see **Supplementary Fig. 3d** for schematic). The bending energy of the elastic beam is given as $\int_0^L \frac{\kappa}{2}\left(\frac{\mathrm{d}\theta}{\mathrm{d}s}\right)^2 \mathrm{d}s$, with κ the bending stiffness of cell

boundary and L the total length (the cell boundary is considered to be inextensible). The binding potential per unit length V is dependent on the deflection distance (y) vertical to the initial profile of cell boundary, that is $V = V(y)$, and the total binding potential should be $\int_0^L V(y)\,\mathrm{d}s$. Besides, geometric variables are mutually dependent and yield the relation $\frac{\mathrm{d}y}{\mathrm{d}s} = \sin\theta$, which works as a geometric constraint in the model. In the end, the total energy of one cell-cell interface can be written as

$$W = \int_0^L \frac{\kappa}{2}\left(\frac{\mathrm{d}\theta}{\mathrm{d}s}\right)^2 \mathrm{d}s + \int_0^L V(y)\,\mathrm{d}s + \int_0^L \gamma(s)\left(\frac{\mathrm{d}y}{\mathrm{d}s} - \sin\theta\right)\mathrm{d}s, \tag{1}$$

where $\gamma(s)$ serves as a Lagrange multiplier.

The local minimum of the total energy W in Eq. (1) corresponds to the equilibrium state of the cell-cell interface. The minimization of energy W results in the following set of nonlinear equations that describe the current profile of cell-cell interface:

$$\begin{aligned} \kappa\frac{\mathrm{d}^2\theta}{\mathrm{d}s^2} + \gamma\cos\theta &= 0, \\ \frac{\mathrm{d}\gamma}{\mathrm{d}s} &= \frac{\partial V(y)}{\partial y}, \\ \frac{\mathrm{d}y}{\mathrm{d}s} &= \sin\theta. \end{aligned} \tag{2}$$

Specifically, the binding potential $V(y)$ has two sources: (i) the tight junction that tends to bind cell boundaries together, (ii) the cellular structures beneath the tissue surface that hinder the deflection of cell boundaries. The binding potential of the tight junction can be modeled as a van der Waals-like potential[4] $V_{\mathrm{TJ}}(y) = w_{\mathrm{TJ}}\left[\left(\frac{d}{y}\right)^9 - \left(\frac{d}{y}\right)^3\right]$, with w_{TJ} a pre-factor that characterizes the adhesion strength, and d an intrinsic interaction distance between the cell boundaries. Even without external mechanical perturbations (e.g., the swelling of goblet cells nearby), the pair of cell boundaries still has a small gap that separate two cells. The width of this gap (i.e., the homeostatic distance between cell boundaries) is $2d_0$ (see **Supplementary Fig. 3d** for schematic, and we can easily obtain $d_0 = \sqrt[6]{3}d$ by considering the local minimum of V_{TJ}). The binding potential that arises from external cell components is assumed to be $V_{\mathrm{ext}}(y) = \frac{w_{\mathrm{ext}}}{2}\left(\frac{y}{d_0} - 1\right)^2$, with w_{ext} a pre-factor that characterize the bridging strength. The external cell components would not affect the mechanical state of cell-cell interface in the homeostatic state of the tissue, but will impose a bridging force that gradually increase with the extension of junctional cracks. Overall, we have the binding potential $V(y) = V_{\mathrm{TJ}}(y) + V_{\mathrm{ext}}(y)$.

To solve the equation system in Eq. (2), we still need to specify the conditions at two endpoints of the cell-cell interface (i.e., the elastic beams). One can notice the parameter $\gamma(s)$ acts as the shear force in the elastic beam. On the side away from the goblet cell (i.e., $s = 0$), there is neither bending moment (which is proportional to $\frac{d\theta}{ds}$) nor shear force (γ): $\frac{d\theta}{ds} = 0$ and $\gamma = 0$ at $s = 0$. At

the side linked to the goblet cell (i.e., $s = L$), the goblet cell generates a peeling force F vertical to the cell-cell interface. This external force acts as a shear force. Thus, we have $\frac{d\theta}{ds} = 0$ and $\gamma = F$ at $s = L$. To simplify the analysis, we non-dimensionalize the parameters. The length-related parameters are normalized by the homeostatic distance d_0, such that $\bar{y} = y/d_0$, $\bar{s} = s/d_0$, and $\bar{L} = L/d_0$. Forces are normalized by the adhesion strength w_{TJ}, yielding $\bar{\gamma} = \gamma/w_{\mathrm{TJ}}$ and $\bar{F} = F/w_{\mathrm{TJ}}$. Under this formulation, the equilibrium configuration of the cell–cell interface depends only on two dimensionless parameters: (i) the effective bending rigidity $\bar{\kappa} = \kappa/(u_0^2 w_{\mathrm{TJ}})$, and (ii) the relative bridging strength $\bar{w} = w_{\mathrm{ext}}/w_{\mathrm{TJ}}$. Now Eq. (2) becomes

$$\bar{\kappa}\frac{\mathrm{d}^2\theta}{\mathrm{d}\bar{s}^2} + \bar{\gamma}\cos\theta = 0,$$
$$\frac{\mathrm{d}\bar{\gamma}}{\mathrm{d}\bar{s}} = \sqrt{3}(\bar{y}^{-4} - \bar{y}^{-10}) + \bar{w}(\bar{y} - 1),$$
$$\frac{\mathrm{d}\bar{y}}{\mathrm{d}\bar{s}} = \sin\theta. \quad (3)$$

To evaluate the extent of junctional cracking, we introduce the "crack opening displacement" (COD) as the deflection distance of the enterocyte boundary at its endpoint adjacent to the goblet cell, i.e., $y(s = L)$. In the initial regime, COD remains small and varies minimally with increasing peeling force F (or the volume expansion of the goblet cell), indicating that the cell-cell junction still holds together. However, once the peeling force F exceeds a threshold, COD rises sharply – or even discontinuously – indicating the onset and progression of a junctional crack, and the failure of the tight junction (**Supplementary Fig. 3e, f**). These theoretical predictions are consistent with classical models[1,2,5,6] and explain well the experimental observation that the swelling of goblet cells promotes junctional fractures (**Fig. 2**). Sensitivity analysis of key mechanical parameters further shows that junctional cracks can form more easily when the cell-cell interface is mechanically weakened (**Supplementary Fig. 3e, f**). Specifically: (i) a decrease in the effective bending rigidity $\bar{\kappa}$ would lower the threshold for the crack nucleation, making the cell-cell interface more prone to breach (**Supplementary Fig. 3e**); (ii) when external cell structures are impaired (characterized as smaller bridging strength $\bar{w}$), crack nucleation is only slightly affected, but subsequent crack propagation becomes easier (**Supplementary Fig. 3f**). However, these predictions are inconsistent with the experimental results obtained upon depletion of myosin IIA (NMIIA): with fewer myosin molecules linking cortical actin filaments, the cell cortex and associated elastic structures become softer. In this scenario, junctional fractures are inhibited in experiments (**Fig. 4b, h**), rather than facilitated as predicted by the model (**Supplementary Fig. 3e, f**).

In this model, the cell-cell interface is treated as an elastic structure. However, epithelial cells can actively remodel cell junctions and adjust their shapes and positions during homeostasis or in response to abnormal mechanical cues [7,8]. To account for these, we next extend the model to include mechanical interaction at the tissue level, aiming to explore how the goblet cell-induced mechanical perturbation impact neighboring enterocytes and their junctions.

2. Crack mechanics of the whole tissue

Epithelial cells interact mechanically through adhesive and elastic forces at the cell-cell interface (see also Section 1 for discussion). Such mechanical interactions between cells can be captured by multiscale tissue models such as vertex models. In classical vertex models[7,9–14], each cell is described as a deformable polygon with multiple vertices, where each vertex represents a tricellular junction where cell boundaries meet, and on which the force balance is written. Although such models can well reproduce cell packing geometry[7], tissue rheology[10,15] and related properties, they inherently assume that cell-cell interfaces remain straight and tightly connected – each cell interface being modeled as a straight line linking two vertices – and thus cannot capture junctional fractures.

Here, to investigate the formation mechanism of junctional cracks, we extend the classical vertex model by considering the cell-cell interface to be bendable and detachable[5,16]. To this end, we still model each cell-cell interface as two cell boundaries initially bonded together (as in Section 1), and discretize each cell boundary as a set of particles linked with elastic springs (**Supplementary 8a**): the mechanical interactions among these particles endow the cell boundary with bending rigidity and extensibility, and the positions of these particles depict the profile of the cell boundary. Importantly, to mimic the adhesive effects of tight junctions, we endow the directly opposite particles (at the paired cell boundaries) with attractive forces to each other. In this way, this model can not only take the changes in cell shape and positions (as in classic vertex models) into account, but also allow for fractures of cell junctions. In the scenario without junctional fracture, this model gives similar predictions to the classic vertex models.

Analogous to the classical vertex models and the interface model proposed in Section 1, the mechanical energy of an enterocyte is mainly generated by changes in cell size (i.e., cell area in two-dimensional modeling) and elastic deformations of the cell surface/boundary (i.e., plasma membrane and cell cortex), the latter of which can be further divided into the boundary extension/contraction and the bending deformation. Thus, the cellular mechanical energy can be formulated as

$$U_E = \frac{K_A}{2}(A - A_0)^2 + \sum_{i=1}^{N} \frac{NK_L}{2}(L_i - L_0)^2 + \sum_{i=1}^{N} \frac{NK_B}{2}(\cos\theta_i - 1)^2 . \quad (4)$$

The first term of the energy U_E comes from the change of the cell area, where K_A is the area rigidity and A (or A_0) is the current (or preferred) cell area. The second term is about the elastic extension/contraction of the cell boundary: let K_L be the extension rigidity, and divide the whole boundary of one cell into N pieces (with an equal length initially), whose current (or preferred) length is L_i (or L_0). Correspondingly, the cell perimeter is $P = \sum_{i=1}^{N} L_i$ and the preferred perimeter is $P_0 = NL_0$. The last term represents the bending resistance of cell boundary, where K_B is the bending rigidity and θ_i is the angle between neighboring pieces of the cell boundary. In numerical simulations, we set $N = 50$. Besides, as the single cells are initially in their round shapes (before being in contact with each other), we set a preferred cell radius $R = 20\mu\text{m}$ and thus calculate the preferred cell area as $A_0 = NR^2 \sin(2\pi/N)/2$.

The mechanical energy of a single enterocyte can be non-dimensionalized as

$$u_E = \frac{U_E}{K_A A_0^2} = \frac{1}{2}(a-1)^2 + \sum_{i=1}^{N} \frac{k_l}{2}\left(l_i - \frac{p_0}{N}\right)^2 + \sum_{i=1}^{N} \frac{k_b}{2}(\cos\theta_i - 1)^2\,, \qquad (5)$$

with $a = A/A_0$ and $l_i = L_i/\sqrt{A_0}$ respectively the dimensionless area and length, and $k_l = NK_L/(K_A A_0)$ and $k_b = NK_B/(K_A A_0^2)$ respectively the dimensionless extensional rigidity and bending rigidity of the cell boundary. The key parameter in Eq. (5) is the perimeter-to-area ratio $p_0 = P_0/\sqrt{A_0}$ (with $P_0 = NL_0$), which also referred to as “target shape index” in some references [10]. This parameter determines the solid-fluid transition of epithelial tissues[10]: the tissue is more of a solid when this shape index is below a threshold, and become fluid after the shape index exceeds the threshold. This is because cells with small perimeters are hard to change shape and position, whereas cells with large perimeters are floppy and tend to move under mechanical loading[15]. Inspired by this, we also quantified the shape index of enterocytes to infer the mechanical properties of the tissue and individual cells. After depletion of myosin IIA (NMIIA), we found that the “shape index” increases (**Fig. 4e**), indicating that some enterocytes become floppy and can more easily change their shape and position, while the tissue in general becomes more fluid.

In addition to the enterocytes discussed above, the intestinal epithelium also contains goblet cells, which are assumed to have mechanical/geometric parameters different from those of enterocytes. For each goblet cell, its free energy of goblet cells U_G still yields the form in Eq. (4):

$$U_G = \frac{K_A^G}{2}(A - A_0^G)^2 + \sum_{i=1}^{N} \frac{NK_L^G}{2}(L_i - L_0^G)^2 + \sum_{i=1}^{N} \frac{NK_B}{2}(\cos\theta_i - 1)^2\,. \qquad (6)$$

Following the same normalization procedure in Eq. (5), one can obtain the dimensionless mechanical energy $u_G = U_G/K_A A_0^2$ as

$$u_{\mathrm{G}} = \frac{1}{2}k_a^G\left(a - a_0^{\mathrm{G}}\right)^2 + \sum_{i=1}^{N} \frac{k_l^G}{2}\left(l_i - \frac{p_0^G}{N}\right)^2 + \sum_{i=1}^{N} \frac{k_b}{2}(\cos\theta_i - 1)^2\,. \qquad (7)$$

To ensure the shape roundness of goblet cells, we endure goblet cells with a small shape index (we set $p_0^G = 2$ in simulations). Besides, given that the goblet cells act as drivers of the deformation and fracturing of enterocytes, it is reasonable to assume goblet cells themselves have larger stiffnesses (we set $k_a^G = 10$ and $k_l^G = 10k_l$ in simulations). Importantly, Eq. (7) includes an additional geometric parameter, that is the normalized preferred area of goblet cells $a_0^{\mathrm{G}} = A_0^{\mathrm{G}}/A_0$. This parameter controls the swelling degree of goblet cells. In our simulations, we first set $a_{\mathrm{G}0} = 0.2$ in the initial step, then increase its value to mimic the swelling process of goblet cells.

The total energy of the intestinal tissue can be written as

$$u_{\mathrm{t}} = \sum_{p=1}^{N_E} u_E^p + \sum_{q=1}^{N_G} u_G^q\,. \qquad (8)$$

Our simulation box contains $N_E = 36$ enterocytes and $N_G = 1$ or 2 goblet cells. We randomly select the initial position inside the bulk for single goblet cells (**Supplementary Fig. 7b),** and for the case with two goblet cells ($N_G = 2$), these two goblet cells have an interval distance of one (or two) enterocyte cells (**Supplementary Fig. 7c**).

The movement of discrete particles at cell boundaries is driven by the changes in the cell energy and cell-cell interactions. In our simulations, the spatio-temporal evolution of particle $\boldsymbol{r}_i$ of cell m yields

$$\gamma \frac{\mathrm{d}\boldsymbol{r}_i}{\mathrm{d}t} = -\frac{\partial U_m}{\partial \boldsymbol{r}_i} + F_{m,i}^{\mathrm{int}}, \tag{S9}$$

where γ is the damping coefficient and U_m is the mechanical energy of cell m (which can be either enterocyte or goblet cell, see Eqs. (4) and (6) for definitions). To avoid overlapping between cells, we also introduce the cell–cell interaction force $F_{m,i}^{\mathrm{int}}$, which is the repulsive force between discrete particles that belonging to different cells. In simulation, we set this repulsive force as $f_0\left(-\frac{1}{|\boldsymbol{r}_{m,i}-\boldsymbol{r}_{n,j}|^2}+\frac{1}{r_{\mathrm{e}}^2}\right)\frac{\boldsymbol{r}_{m,i}-\boldsymbol{r}_{n,j}}{|\boldsymbol{r}_{m,i}-\boldsymbol{r}_{n,j}|}$, where $\boldsymbol{r}_{m,i}$ denotes the vertex i of cell m, f_0 represents the intensity of repulsion, and r_{e} is an effective distance.

Supplementary Table 1. Key parameters used in the model

Physical meaning	Symbol	Value	Reference
area rigidity	K_A	1×10^7 N/m^3	12
extension rigidity	K_L	1×10^{-4} N/m	12
bending rigidity	K_B	6.25×10^{-125} J	16
damping coefficient	γ	0.1 Ns/m	12
repulsive strength	f_0	5×10^{-4} N/m	17
effective distance	r_{e}	1.25 μm	18

This model predicts that the nucleation and extension of junctional cracks depend not only on the size of goblet cells (characterized as a_0^{G}), whose propagation would push the adjacent enterocytes and peel apart their cell junctions (in agreement with the experimental observation in **Fig. 2** and the theoretical prediction in Section 1), but also depends on the general rheological property of the epithelia (implied by the cell shape index p_0): solid-like tissues are hard to deform under mechanical perturbations, and thus more likely to accumulate local stresses that can induce crack nucleation; in contrast, in fluid-like tissues, their enterocytes are floppy and can easily rearrange their positions and adjust their shape/size to adapt to abnormal mechanical forces. Such mechanical self-adjustment prevents stress accumulation and thus effectively protects the tissue from physical damage even if individual cells are mechanically vulnerable. These predictions can well explain the perturbation experiments on cell mechanics (**Fig. 5a-c, h**): after depletion of myosin IIA, the epithelium becomes more deformable (as the cellular shape index increases, see quantification in **Fig. 4e**) and the cracks become fewer (**Fig. 4h**).

Supplementary References

1. Oyharcabal, X. & Frisch, T. Peeling off an elastica from a smooth attractive substrate. *Phys Rev E Stat Nonlin Soft Matter Phys* **71**, 036611 (2005).
2. Peng, Z. & Chen, S. Effect of bending stiffness on the peeling behavior of an elastic thin film on a rigid substrate. *Phys Rev E Stat Nonlin Soft Matter Phys* **91**, 042401 (2015).
3. Kendall, K. Thin-film peeling-the elastic term. *J. Phys. D: Appl. Phys.* **8**, 1449 (1975).
4. J. N. Israelachvili, (1992) Intermolecular and surface forces. Academic Press Ltd., London, 2nd ed.. - References - Scientific Research Publishing. https://www.scirp.org/reference/referencespapers?referenceid=16445.
5. Chen, H., Feng, X., Huang, Y., Huang, Y. & Rogers, J. A. Experiments and viscoelastic analysis of peel test with patterned strips for applications to transfer printing. *Journal of the Mechanics and Physics of Solids* **61**, 1737–1752 (2013).
6. Qian, J., Lin, J., Xu, G.-K., Lin, Y. & Gao, H. Thermally assisted peeling of an elastic strip in adhesion with a substrate via molecular bonds. *Journal of the Mechanics and Physics of Solids* **101**, 197–208 (2017).
7. Farhadifar, R., Röper, J.-C., Aigouy, B., Eaton, S. & Jülicher, F. The influence of cell mechanics, cell-cell interactions, and proliferation on epithelial packing. *Curr Biol* **17**, 2095–2104 (2007).
8. Guillot, C. & Lecuit, T. Mechanics of epithelial tissue homeostasis and morphogenesis. *Science* **340**, 1185–1189 (2013).
9. Chen, Y. *et al.* Activation of Topological Defects Induces a Brittle-to-Ductile Transition in Epithelial Monolayers. *Phys. Rev. Lett.* **128**, 018101 (2022).
10. Bi, D., Lopez, J. H., Schwarz, J. M. & Manning, M. L. A density-independent rigidity transition in biological tissues. *Nature Phys* **11**, 1074–1079 (2015).
11. Boocock, D., Hirashima, T. & Hannezo, E. Interplay between Mechanochemical Patterning and Glassy Dynamics in Cellular Monolayers. *PRX Life* **1**, 013001 (2023).
12. Lin, S.-Z., Li, B., Lan, G. & Feng, X.-Q. Activation and synchronization of the oscillatory morphodynamics in multicellular monolayer. *Proc Natl Acad Sci U S A* **114**, 8157–8162 (2017).
13. Fletcher, A. G., Osterfield, M., Baker, R. E. & Shvartsman, S. Y. Vertex models of epithelial morphogenesis. *Biophys J* **106**, 2291–2304 (2014).
14. Alt, S., Ganguly, P. & Salbreux, G. Vertex models: from cell mechanics to tissue morphogenesis. *Philos Trans R Soc Lond B Biol Sci* **372**, 20150520 (2017).
15. Park, J.-A. *et al.* Unjamming and cell shape in the asthmatic airway epithelium. *Nature Mater* **14**, 1040–1048 (2015).
16. Lv, J.-Q. *et al.* Active hole formation in epithelioid tissues. *Nat. Phys.* **20**, 1313–1323 (2024).
17. Boromand, A., Signoriello, A., Ye, F., O'Hern, C. S. & Shattuck, M. D. Jamming of Deformable Polygons. *Phys. Rev. Lett.* **121**, 248003 (2018).
18. Garcia, S. *et al.* Physics of active jamming during collective cellular motion in a monolayer. *Proceedings of the National Academy of Sciences* **112**, 15314–15319 (2015).
