# Supplementary material for: Goblet cells mechanically breach the epithelial barrier in gut homeostasis
Source: Nat Commun. 2026 Jul 25;17:9078. doi: 10.1038/s41467-026-76034-0 (PMC13518819; doi:10.1038/s41467-026-76034-0)
Supplement: Supplementary file 3 [file 41467_2026_76034_MOESM2_ESM.pdf]

Description of Additional supplementary files

Supplementary Movie 1: Representative z-stack of murine small intestinal tissue stained for mucus granules (WGA, cyan), basolateral junctions (Na^+/K^+-ATPase, magenta), F-actin (phalloidin, grey) and nuclei (DAPI, grey) (z-range, 11,6 µm; z-step, 0.3 µm). Scale bar, 10 µm.

Supplementary Movie 2: Representative z-stack of thick section of control small intestinal tissue stained for mucus granules (WGA, cyan), Tight junctions (ZO-1, magenta), F-actin (phalloidin, grey) and nuclei (DAPI, grey) (z-range, 9.8 µm; z-step, 0.3 µm). Scale bar, 10 µm.

Supplementary Movie 3: Representative z-stack of thick section of succinate treated small intestinal stained for mucus granules (WGA, cyan), Tight junctions (ZO-1, magenta), F-actin (phalloidin, grey) and nuclei (DAPI, grey) (z-range, 11,2 µm; z-step, 0.3 µm). Scale bar, 10 µm.

Supplementary Movie 4: Time-lapse imaging of laser ablation at G-E junction in NMIIA-GFP explants. Laser ablation was performed 5 s after the start of the movie. Total imaging duration was 1 min (1 image/s). Scale bar, 10 µm.

Supplementary Movie 5: Time-lapse imaging of laser ablation at E-E 1 junction in NMIIA-GFP explants. Laser ablation was performed 5 s after the start of the movie. Total imaging duration was 1 min (1 image/s). Scale bar, 10 µm.

Supplementary Movie 6: Time-lapse imaging of laser ablation at E-E 2 junction in NMIIA-GFP explants. Laser ablation was performed 5 s after the start of the movie. Total imaging duration was 1 min (1 image/s). Scale bar, 10 µm.
