# Supplementary material for: Goblet cells mechanically breach the epithelial barrier in gut homeostasis
Source: Nat Commun. 2026 Jul 25;17:9078. doi: 10.1038/s41467-026-76034-0 (PMC13518819; doi:10.1038/s41467-026-76034-0)
Supplement: Supplementary file 4 [file 41467_2026_76034_MOESM9_ESM.pdf]

Corresponding author(s): denis.krndija@utoulouse.fr
xueshilei@westlake.edu.cn

Last updated by author(s): 16/03/2026

Reporting Summary

Nature Portfolio wishes to improve the reproducibility of the work that we publish. This form provides structure for consistency and transparency in reporting. For further information on Nature Portfolio policies, see our Editorial Policies and the Editorial Policy Checklist.

Statistics

For all statistical analyses, confirm that the following items are present in the figure legend, table legend, main text, or Methods section.

n/a	Confirmed	
☐	☒	The exact sample size (*n*) for each experimental group/condition, given as a discrete number and unit of measurement
☐	☒	A statement on whether measurements were taken from distinct samples or whether the same sample was measured repeatedly
☐	☒	The statistical test(s) used AND whether they are one- or two-sided<br>*Only common tests should be described solely by name; describe more complex techniques in the Methods section.*
☐	☒	A description of all covariates tested
☐	☒	A description of any assumptions or corrections, such as tests of normality and adjustment for multiple comparisons
☐	☒	A full description of the statistical parameters including central tendency (e.g. means) or other basic estimates (e.g. regression coefficient) AND variation (e.g. standard deviation) or associated estimates of uncertainty (e.g. confidence intervals)
☐	☒	For null hypothesis testing, the test statistic (e.g. *F*, *t*, *r*) with confidence intervals, effect sizes, degrees of freedom and *P* value noted<br>*Give P values as exact values whenever suitable.*
☒	☐	For Bayesian analysis, information on the choice of priors and Markov chain Monte Carlo settings
☒	☐	For hierarchical and complex designs, identification of the appropriate level for tests and full reporting of outcomes
☒	☐	Estimates of effect sizes (e.g. Cohen's *d*, Pearson's *r*), indicating how they were calculated

Our web collection on statistics for biologists contains articles on many of the points above.

Software and code

Policy information about availability of computer code

Data collection	Zen Black (11.0.0.190). Custom codes are deposited on Code Ocean (https://codeocean.com/capsule/7420253/tree; https://doi.org/10.24433/CO.7420253.v1).
Data analysis	Fiji ImageJ (2.16.0/1.54p), GraphPad Prism 8 (8.0.1), Affinity Designer (1.10.8). Custom codes are deposited on Code Ocean (https://codeocean.com/capsule/7420253/tree; https://doi.org/10.24433/CO.7420253.v1).

For manuscripts utilizing custom algorithms or software that are central to the research but not yet described in published literature, software must be made available to editors and reviewers. We strongly encourage code deposition in a community repository (e.g. GitHub). See the Nature Portfolio guidelines for submitting code & software for further information.

Data

Policy information about availability of data

All manuscripts must include a data availability statement. This statement should provide the following information, where applicable:

- Accession codes, unique identifiers, or web links for publicly available datasets
- A description of any restrictions on data availability
- For clinical datasets or third party data, please ensure that the statement adheres to our policy

Data supporting the findings of this study are available within the paper and its Supplementary Information. Microscopy data reported in this paper will be shared

by the lead contact on request, due to large file sizes. Source data are provided with this paper.

Research involving human participants, their data, or biological material

Policy information about studies with human participants or human data. See also policy information about sex, gender (identity/presentation), and sexual orientation and race, ethnicity and racism.

Reporting on sex and gender	No phenotypic differences were observed between males and females, mice of both sexes were used in all experiments
Reporting on race, ethnicity, or other socially relevant groupings	N/A
Population characteristics	N/A
Recruitment	Human intestinal biopsies were obtained from the CHU Rangueil in Toulouse with informed consent from each patient (MTA 24 134C). Patients were diagnosed with inflammatory pathologies, and biopsies of ileum were taken from non-inflamed regions.
Ethics oversight	All mouse husbandry and handling procedures were approved and conducted in accordance with European and national regulation (Protocol Authorization APAFIS #38994-2023062718345638)

Note that full information on the approval of the study protocol must also be provided in the manuscript.

Field-specific reporting

Please select the one below that is the best fit for your research. If you are not sure, read the appropriate sections before making your selection.

☒ Life sciences ☐ Behavioural & social sciences ☐ Ecological, evolutionary & environmental sciences

For a reference copy of the document with all sections, see nature.com/documents/nr-reporting-summary-flat.pdf

Life sciences study design

All studies must disclose on these points even when the disclosure is negative.

Sample size	No formal sample size calculation was performed. Sample sizes were based on prior experience with intestinal tissue imaging, organoid experiments and mouse studies, while minimizing animal use. In vivo experiments included at least three independent biological replicates (individual mice) per condition unless otherwise stated. For organoid experiments, biological replicates were independent cultures. Multiple fields, cells, junctions or organoids were analysed per biological replicate as technical replicates. Exact n values are provided in the figure legends. Sample sizes were sufficient to detect reproducible differences using the statistical methods described.
Data exclusions	No data were excluded from the analysis.
Replication	For each condition, at least three independent technical and three biological replicates were performed, and reproducibility was confirmed by statistical analysis described in the Methods section.
Randomization	For each treatment (succinate, DSS, permeability), mice were assigned and treated in a randomized order.
Blinding	Blinding was not possible, as the same investigator genotyped, treated, and performed the experiment on the mice.

Reporting for specific materials, systems and methods

We require information from authors about some types of materials, experimental systems and methods used in many studies. Here, indicate whether each material, system or method listed is relevant to your study. If you are not sure if a list item applies to your research, read the appropriate section before selecting a response.

Materials & experimental systems

n/a	Involved in the study	
☐	☒	Antibodies
☒	☐	Eukaryotic cell lines
☒	☐	Palaeontology and archaeology
☐	☒	Animals and other organisms
☒	☐	Clinical data
☒	☐	Dual use research of concern
☒	☐	Plants

Methods

n/a	Involved in the study	
☒	☐	ChIP-seq
☒	☐	Flow cytometry
☒	☐	MRI-based neuroimaging

Antibodies

Antibodies used

rat anti-ZO-1, Millipore, MABT11
rat anti-E-cadherin, Invitrogen, 131900
rabbit anti-occludin, Proteintech, 27260-1-AP
sheep anti-desmocollin-2, Biotechne, AF7490
Rabbit anti-tricellulin, Invitrogen, 48-8400
mouse anti-desmoplakin, Progen, 651109
rabbit anti-phospho-Myosin Light Chain (P-MLC) (T18/S19), Cell Signaling, 3674S
rabbit anti-Na/K ATPase, Abcam, ab-76020
rabbit NMIIA, Biolegend, 909801
mouse anti-ZO-1, Invitrogen, 1A12
rhodamine-phalloidin, Invitrogen, R415
Alexa-647-phalloidin, Invitrogen, A30107
WGA-rhodamine, VectorLabs, RL-1022
rabbit anti-NMIIA, Biolegend, 909801
rabbit anti-GAPDH, Sigma, G9545
horseradish peroxidase-conjugated anti-rabbit, Invitrogen, G21234
All secondary antibodies were purchased from Molecular probes

Validation

Primary antibodies were commercially sourced and validated by the manufacturers for the applications used, according to the corresponding product documentation. Antibody specificity was further assessed in our samples by confirming the expected staining pattern and subcellular localization: junctional staining for ZO-1, occludin, E-cadherin, tricellulin, desmocollin and desmoplakin; apical/junctional staining for phospho-MLC and NMIIA; epithelial keratin staining for CK8; mucus-associated staining for WGA; and the expected GAPDH signal by western blotting. Antibody sources, catalogue numbers and dilutions are listed in the Methods section.

Animals and other research organisms

Policy information about studies involving animals; ARRIVE guidelines recommended for reporting animal research, and Sex and Gender in Research

Laboratory animals

All mice used in this study were Mus musculus. Mice were housed in the Specific and Opportunistic Pathogen-Free (SOPF) animal facility of the Centre de Biologie Intégrative (CBI, Toulouse, France) under standard husbandry conditions, with a 12 h light/12 h dark cycle, ambient temperature of 22 ± 2°C and relative humidity of 55 ± 10%. Animals were monitored daily in accordance with institutional animal welfare guidelines.
Comparisons were performed using age-matched littermates aged 8–12 weeks, unless otherwise stated. Both male and female mice were used in all experiments. Wild-type mice were C57BL/6N. Transgenic mouse lines were maintained on a predominantly C57BL/6N genetic background.
Villin-CreERT2 mice were kindly provided by Dr D. Matić Vignjević (Institut Curie, Paris, France). Myh9^fl/fl^ mice were obtained from the Mutant Mouse Resource and Research Center (MMRRC; stock no. 032096-UNC) and crossed with Villin-CreERT2 mice to generate intestinal epithelium-specific NMIIA knockout mice (Villin-CreERT2; Myh9^fl/fl^, referred to as NMIIA-KO). Myh9^fl/fl^ mice were also crossed with Muc2-CreERT2 mice, kindly provided by Dr C. Lengner (University of Pennsylvania, USA), to generate Muc2-CreERT2; Myh9^fl/fl^ mice. NMIIA-GFP mice were kindly provided by Dr D. Matić Vignjević (Institut Curie, Paris, France).

Wild animals

No wild animals were used.

Reporting on sex

No phenotypic differences were observed between males and females, mice of both sexes were used in all experiments

Field-collected samples

N/A

Ethics oversight

All mouse husbandry and handling procedures were approved and conducted in accordance with European and national regulation (Protocol Authorization APAFIS #38994-2023062718345638).

Note that full information on the approval of the study protocol must also be provided in the manuscript.

Plants

Seed stocks	N/A
Novel plant genotypes	N/A
Authentication	N/A
